# Physiology and Pathophysiology of Marathon Running: A narrative Review

**DOI:** 10.1186/s40798-025-00810-3

**Published:** 2025-01-27

**Authors:** Lorin Braschler, Pantelis T. Nikolaidis, Mabliny Thuany, Daniela Chlíbková, Thomas Rosemann, Katja Weiss, Matthias Wilhelm, Beat Knechtle

**Affiliations:** 1 Centre for Rehabilitation and Sports Medicine, Inselspital, University Hospital of Bern, University of Bern, Bern, Switzerland; 2 School of Health and Caring Sciences, University of West Attica, Athens, Greece; 3 Department of Physical Education, State University of Para, Pará, Brazil; 4 Brno University of Technology, Centre of Sport Activities, Brno, Czechia; 5 Institute of Primary Care, University of Zurich, Zurich, Switzerland; 6 Medbase St. Gallen Am Vadianplatz, Vadianstrasse 26, 9001 St. Gallen, Switzerland

**Keywords:** Endurance training, sports performance, Sex differences, Athletic injury, Exercise overtraining

## Abstract

**Background:**

Marathon training and running have many beneficial effects on human health and physical fitness; however, they also pose risks. To date, no comprehensive review regarding both the benefits and risks of marathon running on different organ systems has been published.

**Main Body:**

The aim of this review was to provide a comprehensive review of the benefits and risks of marathon training and racing on different organ systems. A predefined search strategy including keywords (e.g., marathon, cardiovascular system, etc.) and free text search was used. Articles covering running regardless of sex, age, performance level, and event type (e.g., road races, mountain marathons) were considered, whereas articles examining only cycling, triathlon, stress-tests or other sports were excluded. In total, we found 1021 articles in PubMed, Scopus, and Google Scholar, of which 329 studies were included in this review. Overall, marathon training offers several benefits for different organ systems and reduces all-cause mortality. As such, it improves cardiovascular risk factors, leads to favorable cardiac adaptations, enhances lung function, and improves quality of life in chronic kidney disease patients. It also enhances gastrointestinal mobility and reduces the risk of specific tumors such as colorectal cancer and hepatocellular carcinoma. Marathon training enhances bone health and skeletal muscle metabolism. It further positively affects hematopoiesis and cytotoxic abilities of natural killer cells, and may act neuroprotective on a long-term basis. After a marathon, changes in biomarkers suggesting pathological events in certain organ systems such as cardiovascular, renal, gastrointestinal, liver, hematological, immune, musculoskeletal, central nervous, and endocrine systems can often be observed. Mostly, these changes are limited to 1–3 days post-race and usually normalize within a week. Moreover, marathon running poses the risk of serious adverse events such as sudden cardiac death or acute liver failure. Concerning lung function, a decrease after a marathon race was observed. Acute kidney injury, as well as electrolyte imbalances, are relatively common amongst marathon finishers. Many runners complain of gastrointestinal symptoms during or after long-distance running. Many runners suffer from running-related musculoskeletal injuries often impairing performance. A marathon is often accompanied by an acute inflammatory response with transient immunosuppression, making runners susceptible to infections. Also, hormonal alterations such as increased cortisol levels or decreased testosterone levels immediately after a race are observed. Disturbances in sleep patterns are commonly found in marathon runners leading up to or directly after the race.

**Conclusion:**

All in all, marathon training is generally safe for human health and individual organ systems. Considering the high popularity of marathon running, these findings supply athletes, coaches, sports scientists, and sports medicine practitioners with practical applications. Further large-scale studies examining long-term effects on the cardiovascular, renal, and other system are needed.

## Key Points

- Marathon training and racing affects several organ systems in a positive and negative way.
- The most common problems during or after a marathon are acute kidney injury, gastrointestinal distress, running-related injuries, and upper respiratory tract infections.
- Generally, marathon training is safe for different organ systems and its positive effects outweigh any potential harms

## Background

The traditional marathon, defined as a running event covering 42.195 km [1], enjoys great popularity amongst athletes and the scientific community [2]. Physical activity, such as marathon training, is essential for several aspects of human health [3]. Regular moderate exercise is an essential part of the prevention of hypertension [4], hypercholesterolemia [4], obesity [5], diabetes [4], coronary artery disease (CAD) [3], and cancer [6], positively affects sleeping pattern [7] together with mental health [8], and prolongs life expectancy [7]. Nonetheless, intense bouts of exercise, such as a marathon, may harm and alter certain organ systems and their function, leading to serious adverse effects, such as sudden cardiac death (SCD) [9], or exertional heat stroke (EHS) [10].

So far, there has been a substantial amount of research focused on marathon-induced adaptations of the cardiovascular [3], renal [11], respiratory [12], gastrointestinal (GI) [13], hepatobiliary [14], musculoskeletal [15], hematological [16], immune [17], endocrine [18], central nervous system [19], along with psychological changes [20], thermoregulation [21], and the skin [22]. Another focus of the research was performance aspects such as aerobic capacity [23–25], pacing [26–29], atypical running biomechanics [30], training [31], and tapering strategies [32], alongside anthropometric characteristics [33–36] as well as environmental factors such as ambient temperature [2, 29, 37, 38], air pollution [38–40], weather conditions including humidity [29], wind speed [38] as well as different gradients and surfaces [41].

Many articles covering adverse events in the context of marathon running have been published regarding the cardiovascular system and cardiac function [42–44], acute kidney injury (AKI) [45–47], electrolyte imbalances [48, 49], lung function [50], GI distress [51, 52], liver failure [10], acute inflammatory responses [16], infections, mainly of the upper respiratory tract [53], musculoskeletal injuries [54], central effects alongside sleep disturbances [55, 56], hormonal imbalances [57], thermoregulation [58, 59], and overtraining syndrome [60, 61].

However, to our knowledge, there is no comprehensive review summarizing both the benefits and risks of marathon training and running on organ systems such as the cardiovascular, renal, respiratory, GI, hepatobiliary, hematological, immune, musculoskeletal, endocrine, and central nervous systems so far. Because of its immense popularity, understanding the extent and prevalence of both positive and negative effects alongside their associated factors is of great interest and importance to athletes, coaches, event organizers, sports scientists, and sports medicine practitioners.

Therefore, the aim of this narrative review is to comprehensively analyze the most important physiological and pathophysiological adaptations of marathon training and the race itself on the cardiovascular, renal, respiratory, GI, hepatobiliary, hematological, immune, musculoskeletal, endocrine, and central nervous systems with a focus on a practical relevance for athletes, coaches, event organizers, sports scientists, and sports medicine practitioners.

## Methods

This present review was designed as a narrative review. The current and relevant literature for this study was searched using a predefined search strategy. We searched the following three most common databases for relevant literature to our search question: Scopus, MEDLINE Ovid (PubMed), and Google Scholar [62]. A combination of medical subject headings and free-text words was used to create an accurate and specific literature search. The following keywords were applied in our literature search: “marathon*”, “marathon run*”, “marathon-run*”, “cardiovascular system*”, “digestive system*”, “gastrointestinal system*”, “endocrine system*”, “immune system*”, “muscular system*”, “skeletal system*”, “nervous system*”, “renal system*”, “reproductive system*”, “respiratory system*”, and “hematological system*”. This general search was expanded by an extended specific search with the following keywords: “liver*”, “hematologic diseas*”, “blood diseas*”, “blood coagulation disord*”, “hypercoagulabilit*”, “thrombophil*”, “blood clot*”, thrombos*”, “muscle*”, “bone*”, “musculoskeletal system*”, “musculoskeletal diseas*”, “orthopedic disord*”, “osteoarthrit*”, “central nervous system*”, “cerebrospinal axi*”, “brain*”, “spinal cord*”, “cognition*”, “cognitive function*”, “sleep*”, “sleep habit*”, and “sleeping habit*”. The full search was performed in November 2023, and all found records were imported into the Zotero 6.0.30 Software (Roy Rosenzweig Center for History and New Media at George Mason University). Original articles, reviews, and case reports covering running, which were published in English or German, as well as articles exclusively with complete available full text, were considered for inclusion. This incorporates all athletes regardless of age, both female and male athletes, athletes of all performance levels (i.e., elite and recreational runners), as well as the different event types (e.g., mountain marathons, road races, off-road races, etc.). Other articles investigating solely cycling, triathlon, stress tests, or other sports were excluded. Particularly, articles of relevant significance for both athletes and coaches as well as physicians and event organizers, were selected for this review. Altogether, we found 1,021 articles across the three databases. After an initial screening process (title and abstract) of the retrieved literature, 729 were excluded since they did not meet the inclusion criteria. An additional citation search was performed from the reference list of those articles already selected to be included in the review. In total, 329 articles were considered for this narrative review.

The included articles were all screened for biomarker changes, and data were manually retrieved. Accordingly, graphs for the most relevant biomarkers were crafted with corresponding reference values. Results are depicted as mean and standard deviation unless stated otherwise.

### Changes in Metabolites and Hormones

Several different organ systems are affected by intense bouts of exercise, such as running a marathon, in both positive and negative ways. These shifts are predominantly observable by biomarker changes (e.g., metabolites or hormones) after the completion of a marathon. Table 1 depicts a comprehensive summary of changes in laboratory biomarkers of different organ systems affected by marathon running.

**Table 1:** Alterations in hormones and metabolite biomarkers during a marathon [16, 36, 42, 45, 46, 48, 57, 59, 84–86, 88, 89, 91, 93, 103, 106–109, 137, 148, 156, 157, 195, 199, 243, 246, 252, 254, 271–273, 286–288, 290, 294, 295, 306, 308, 311, 314, 316, 325, 326, 330–332, 358]

*Markers of skeletal and heart muscle damage*	
Creatine kinase	↑/↑↑
Creatine kinase-MB	↑
Myoglobin	↑
Cardiac troponins	↑
Lactate dehydrogenase	↑
*Hepatobiliary biomarkers*	
Alkaline phosphatase	↓/=/↑
Gamma glutamyltransferase	↑
Alanine aminotransferase	↑
Aspartate aminotransferase	↑
Bilirubin	↑
*Kidney markers and electrolytes*	
Creatinine	↑/↑↑
Cystatin C	↑
Blood urea nitrogen	↑
Uric acid	↑
Sodium	↓/=/↑
Potassium	↓/=/↑
Chloride	=
Calcium	↑/=
Phosphate	↑
Magnesium	↓
Protein	↑
Albumin	↑
*Blood count and metabolism*	
Plasma volume	↑/↓
Erythrocytes	↓/=/↑
Leukocytes	↑/↑↑
Thrombocytes	↑
Haptoglobin	↑↓
Iron	↑
Ferritin	↑
Transferrin	↑
D-dimer	↑/↑↑
Fibrinogen	↓
*Inflammatory biomarkers*	
C-reactive protein	↑/↑↑
Interleukin-6	↑
Interleukin-8	↑/↑↑
Interleukin-10	↑/↑↑
Tumor necrosis factor α	↑
*Hormonal alterations*	
Testosterone	↓
Cortisol	↑/↑↑
Adrenocorticotropic hormone	↑
Noradrenaline	↑
Adrenaline	↑
Dopamine	↑
Growth hormone	↑
Prolactin	↑
Thyroid-stimulating hormone	↑/=
Triiodothyronine	↑/=
Thyroxine	↑/=
Vasopressin	↑
Aldosterone	↑
Renin	↑
Atrial natriuretic peptide	↑
N-terminal prohormone of brain natriuretic peptide	↑/↑↑
Erythropoietin	↑
*Energy metabolism*	
Glucose	↑
Free fatty acids	↑/↑↑
Cholesterol	↓
Low-density lipoprotein	↓
High-density lipoprotein	↑

### Cardiovascular System

Endurance exercise is well known for its many beneficial effects on cardiovascular health, such as lower resting arterial blood pressure, improved endothelial function, and higher tolerance of myocardial ischemia [3]. Nevertheless, a marathon puts immense stress on the cardiovascular system, which can upset the balance of this delicate system. We identified 54 studies examining the beneficial effects as well as pathological changes of marathon training and running on the cardiovascular system. Table 2 lists the main findings and results of the said studies.

**Table 2:** Overview of key findings from studies investigating effects of marathon training and running on the cardiovascular system

Subjects	Intervention/Observation	Outcome	References
N = 138 Female = 70 Age 37 (IQR 21–69) years	Differences in exercise-induced cardiovascular remodeling in young and middle aged healthy sedentary individuals	Predominant cardiac remodeling such as increased LV mass and increased ventricular cavity size in the younger group Predominant vascular remodeling such as reduced aortic compliance and reduced systemic vascular resistance and lower resting arterial blood pressure in the older group	[72]
N = 18 Female = 1 Age 52.3 ± 8.0 years	Regular exercise may improve sympathovagal balance and QT variability and counteract the ageing process	Regular exercise leads to a decrease in QT variability Decrease in QT variability is cardioprotective and seems to counteract some effects of ageing on heart control	[81]
N = 108 Female = 0 Age 57.2 ± 5.7 years	Risk of coronary events in marathon runners and associated risk factors	Marathon runners had a 42% higher HDL cholesterol, an 18% lower LDL cholesterol, a 19% lower rate of ever smoking and a 15% lower BMI than age-matched controls resulting in a 51% lower mean 10 years FRS A CAC Score ≥ 100 was found in 36% of runners, generally suggesting an underestimation of cardiovascular risk in asymptomatic runners	[69]
N = 15 Female = 1 Age 43 (IQR 39–48) years	Impact of ultra-marathon and marathon on biomarkers of myocyte necrosis and cardiac congestion	Immediately after the race, elevations of cardiac Troponin I with median value at 0.028 ng/l (IQR 0.022–0.049), CK levels up to 425 U/l (327–681) and NT-proBNP at 132 ng/l (64–198) was observed Significantly higher biomarkers elevation after ultra-marathon compared to marathon	[84]
N = 35 Female = 0 Age 39 ± 8 years	Changes and dynamics of cardiac biomarkers during and after a marathon race	After marathon, elevation in CK up to 411 U/L (± 170) and hs-cTnI up to 0.06 (± 0.09) hs-cTnI returned to baseline levels after 72 h	[42]
N = 102 Female = 0 Age 42.0 ± 9.5 years	72-h kinetics of hs-cTnT and inflammatory markers after marathon running	Directly after the marathon race, hs-cTnT increased 10.8-fold with median values at 31.07 ng/l (IQR 19.3–46.9) and NT-proBNP increased 3.3-fold with median values at 92.6 ng/l (56.9–149.7) compared to values one week pre-race Inflammatory biomarker IL-6 increased 15.5-fold with median values at 31.9 ng/l (20.7–41.5), IL-10 increased 5.6-fold with median values at 28.5 (10.8–49.7) and TNF-α increased 1.1-fold with median values at (9.3–12.3) compared to values one week pre-race	[85]
N = 129 Female = 45 Age 42 ± 14 years	Dynamics of cardiac injury markers after a half-marathon and marathon race in non-elite marathon runners	Directly after the race in the marathon group, increase in myoglobin by 16.0-fold with median values at 626 mg/l (IQR 135–880), CK by 3.3-fold with median values at 432 U/L (112–705) and cTnT elevation with median values at 0.036 (0.011–0.225) compared to baseline values	[86]
N = 19 Female = 2 Age 44.7 ± 9.2 years	Influence of marathon running on plasma levels of ANP	After the marathon race, a 1.8-fold increase in proANP could be observed with median at 3.9 (IQR 2.6–4.8) compared to baseline values	[109]
N = 183 Female = 0 Age 39 ± 9 years	Long-term endurance sport practice increases the incidence of lone atrial fibrillation	Incidence of lone AF was found to be higher in male marathon runners compared to a sedentary control group with reported hazard ratio of 8.8 (95% CI 1.3–61.3) Annual incidence of AF in marathon runners was 0.43/100 and 0.11/100 in a sedentary control group	[96]
N = 121 Female = 61 Age 42 ± 7 years	Sex differences of atrial and ventricular remodeling in non-elite marathon runners	Male athletes have a higher risk of developing AF Male marathon runners were found to have longer-signal-averaged P-wave duration and greater absolute LA volumes compared to the female control groups	[95]
N = 50 Female = 0 Age 56 (IQR 53–65)	Non-ischemic fibrosis in veteran endurance athletes using cardiac MRI and correlation with premature ventricular beats on 24-h ECG	Late gadolinium enhancement corresponding to myocardial fibrosis was seen in 48% of athletes Fibrosis was located mid-myocardial in the basal-lateral LV wall and had significantly lower rest myocardial blood flow 24-h ECG showed a greater burden of premature ventricular beats (by sixfold), additionally to a higher prevalence of ventricular couplets and triplets (by 4.1-fold) in veteran athletes with myocardial fibrosis compared to those without fibrosis	[100]
N = 60 Female = 19 Age 41 (IQR 21–65) years	Myocardial injury and ventricular dysfunction in relation to different training levels of non-elite marathon runners	After the race LV dimensions and EF remained unchanged RV dimensions increased by 6mm to 41mm (± 4) and RV percentage area change decreased from 41 ± 7% to 33 ± 7% An increased mPAP from 12 ± 3 to 25 ± 9 mmHg was measured Participants with less training were at an increased risk of cardiac dysfunction and injury	[93]
N = 44 Female = 0 Age 40 ± 8 years	RV dysfunction after a marathon race	After the marathon race a 1.1-fold increase in RV end-diastolic dimension was observed suggesting RV diastolic dysfunction All values returned to pre-race levels after two weeks	[43]
N = 40 Female = 4 Age 37 ± 8 years	RV dysfunction and structural changes after long-distance races using cardiac MRI	After the race, RV volumes increased and RVEF decreased by 9% LV volumes decreased and LVEF remained unchanged Statistically significant correlations between RVEF and levels of post-race cTnI and BNP were observed The change in RVEF was negatively correlated with race duration On cardiac MRI, 12.8% of athletes showed delayed gadolinium enhancement in the interventricular septum which correlated with higher RV volumes post-race and lower RVEF	[92]
N = 40 Female = 4 Age 37 ± 8 years	Association between inflammatory cytokines and cardiac dysfunction after intense exercise	A total of 25% athletes experienced exercise-induced cardiac dysfunction After the race, IL-6, IL-8, and IL-10 increased by 8.5-, 2.9-, and 7.1-fold, with higher post-race levels of pro-inflammatory cytokines IL-12p70 (by 3.2-fold) and TNFα (by 3.3-fold) in runners with cardiac dysfunction compared to those with normal cardiac function TNFα and IL-12p70 significantly correlated with the change in RVEF and BNP	[105]
N = 108 Female = 0 Age 57.2 ± 5.7 years	LV volumes and mass in experienced marathon runners aged ≥ 50 and their association with cardiovascular risk factors	A CAC score ≥ 100 in context of threshold for elevated risk in middle aged asymptomatic men was observed in 37.1% of marathon runners A LV muscle mass ≥ 150 g in athletes was associated with significantly higher CAC scores than runners with LVMM ≤ 150 g	[44]
N = 21,758 Female = 0 Age 51.7 ± 8.4 years	CAC, all-cause and cardiovascular mortality in relation to physical activity levels	Men with > 3,000 MET-min/week had a RR of 1.11 of having a CAC score ≥ 100 compared to those with less physical activity No significant difference in all-cause mortality was found, between men with > 3,000 MET-min/week compared to men with < 1500 MET-min/week, given a CAC score ≥ 100 in both groups Men with a CAC score < 100 and physical activity > 3000 MET-min/week had a HR of 0.52 for all-cause mortality compared to men with physical activity < 1500 MET-min/week	[115]
N = 10,871,000 Female = 5,140	Cardiac arrest during marathon and half-marathon races	The incidence rate of cardiac arrest was reported at 0.54 per 100,000 participants with higher rates during marathons (1.01 per 100,000) compared to half-marathons (0.27 per 100,000) Male runners had a higher incidence of cardiac arrest (0.90 per 100,000) compared to female participants (0.16 per 100,000) as well as a higher incidence of fatal cardiac arrest 0.62 vs. 0.14 per 100,000 In total, 59 cardiac arrests (mean age 42 ± 13 years; 51 men) were reported during the study period, of which 42 (71%) cases were fatal (incidence, 0.39 per 100,000). Cardiac arrests during marathon races were more often fatal (incidence 0.63 per 100,000) compared to those occurring during half-marathons (incidence 0.25 per 100,000) Among survivors, the most common cause of cardiac arrest was ischemic heart disease (16%) Most common cause of cardiac arrest among non-survivors was hypertrophic cardiomyopathy plus additional diagnoses i.e., CAD or myocarditis (16%), hypertrophic cardiomyopathy (10%), suspected hypertrophic cardiomyopathy (23%), and other causes such as hyponatremia, hyperthermia, and arrhythmogenic right ventricular cardiomyopathy Strongest predictors of survival were initiation of bystander-provided cardiopulmonary resuscitation and causes other than hypertrophic cardiomyopathy	[116]
N = 353,020 Female = 77,664	Life-threatening and major cardiac events during long-distance races	During the prospective study period 25 major cardiovascular events were recorded (2.33, 95% CI 1.58–3.44 per 100,000), all affected male participants 64% of major cardiovascular events were due to myocardial ischemia such as acute coronary syndrome or chronic coronary syndrome, 16% due to genetic cardiac anomalies such as arrhythmogenic right ventricular cardiomyopathy or Brugada syndrome No fatalities were recorded	[9]

*AF* atrial fibrillation, *BMI* body mass index, *CAC* coronary artery calcium, *CAD* coronary artery disease, *CK* creatine kinase, *ECG* electrocardiogram, *EF* ejection fraction, *FRS* Framingham Risk Score, *HDL* high density lipoprotein, *hs-cTnI* high-sensitivity cardiac troponin I, *hs-cTnT* high-sensitivity cardiac troponin T, *IQR* interquartile range, *LDL* low density lipoprotein, *LA* left atrium, *LV* left ventricle, *LVEF* left ventricular ejection fraction, *LVMM* left ventricular muscle mass, *MET* metabolic equivalent of task, *mPAP* mean pulmonary artery pressure, *MRI* magnetic resonance imaging, *NT-proBNP* N-terminal prohormone of brain natriuretic peptide, *proANP* pro atrial natriuretic peptide, *QT* QT interval, *RV* right ventricle, *RVEF* right ventricular ejection fraction, *TNF-α* tumor necrosis factor α, *95% CI* 95% confidence interval

#### Influence on Cardiovascular Risk Profile and Positive Remodeling

Several studies showed endurance athletes to have lower risk factors for CAD compared to sedentary control groups [63–66]. For this reason, regular exercise, such as marathon training, is an important component in the prevention of CAD [3]. However, a recent systematic review of the cardiovascular disease risk factor profile of experienced male amateur marathon runners found that although marathon training can produce positive health benefits, excessive training and marathon running can also decrease benefits from exercise and itself does not negate the long-term health effects of atherosclerosis and cardiovascular disease caused by past negative lifestyle [67].

In general, marathon runners have lower levels of serum low-density lipoprotein (LDL) cholesterol and triglycerides as well as higher levels of high-density lipoprotein (HDL) cholesterol than age-matched controls (see Tables 1 and 2) [3, 64, 66, 68–70]. Furthermore, endurance athletes have significantly lower values for resting blood pressure and pulse rate compared to inactive controls, which have protective effects on both the heart and the vasculature of various organs [66, 71–74]. Even cardiac function is reported to be superior in endurance athletes as stroke volume increases in trained endurance athletes [66, 75]. Intense and long-distance endurance exercise leads to physiological cardiac adaptations, which are commonly referred to as the “athlete’s heart” [3, 76]. Characteristic changes include a balanced volume increase of the left (LV) and the right ventricle (RV) [70, 77], increased LV wall thickness and a gain in myocardial mass [68, 72, 76, 78]. These changes result in enhanced diastolic function due to reduced filling times and ultimately lead to an increase in cardiac output during endurance exercise [3, 79].

Regular physical activity seems to alter the function and influence of the autonomic nervous system (ANS) as endurance athletes have significantly lower heart rates than sedentary controls [79]. Electrocardiogram (ECG) studies found sinus bradycardia in 80% of trained athletes, whereas only 19% of non-athletes exhibited a heart rate < 60 min^−1^ [80]. Enhanced vagal and decreased sympathetic tone to the heart benefits cardiovascular health and reduces the risk for cardiovascular events [81]. A recent study investigating QT interval (QT) variability in middle-aged marathon runners found an overall decrease in QT variability (see Table 2) despite the increasing age of study participants [81]. Increased QT variability is associated with SCD and could be observed in cardiac as well as non-cardiac conditions such as CAD and diabetes mellitus [82, 83].

#### Biomarkers of Cardiovascular Damage

**Fig. 1:**
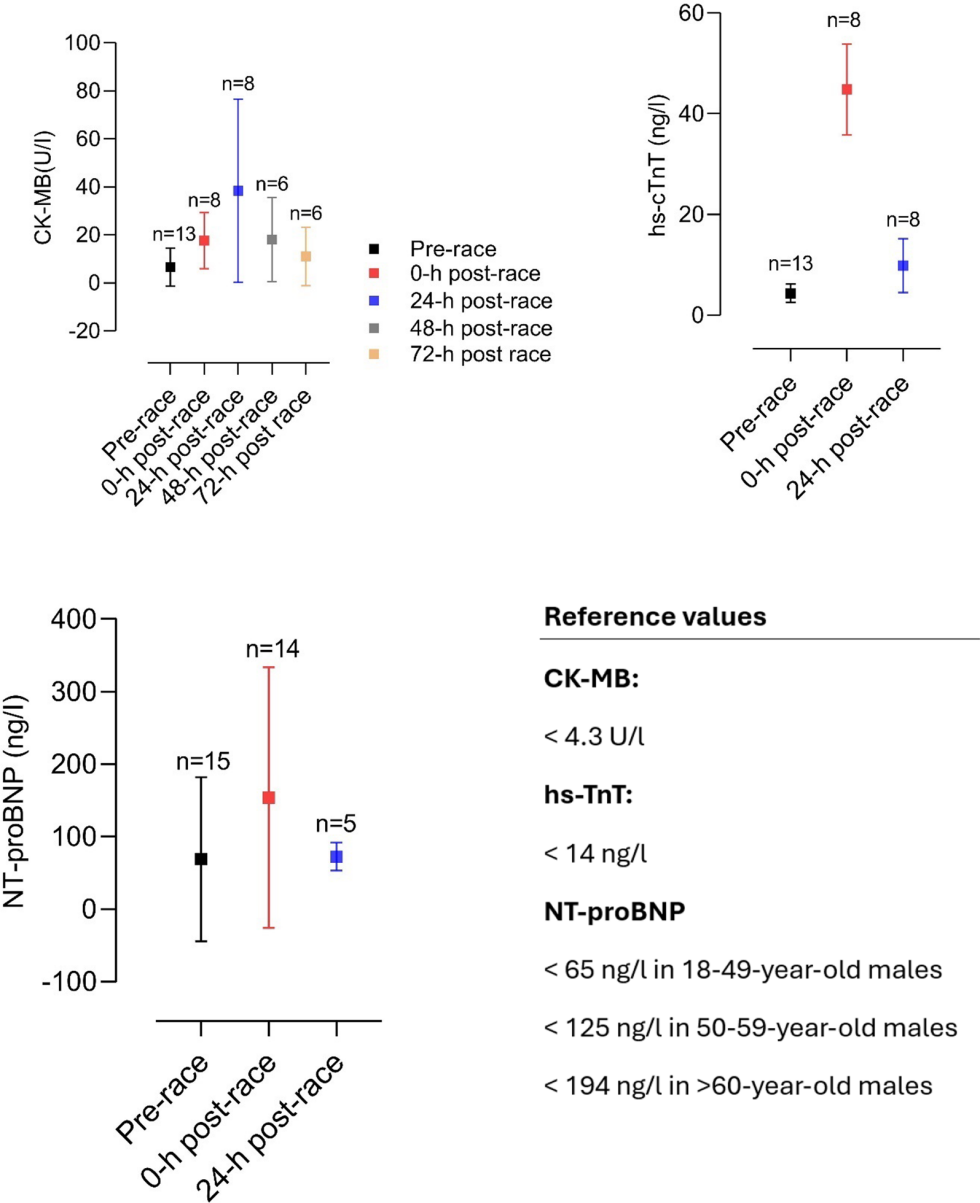
Mean changes in CK-MB, hs-cTnT, and NT-proBNP in studies investigating cardiac biomarkers before and after a marathon [42, 45, 84, 85, 87–89, 91, 93, 94, 102, 103, 196, 243–246, 252, 252, 253, 311, 311, 363]. CK-MB, creatine kinase myocardial band; hs-TnT, high-sensitive cardiac troponin T; NT-proBNP, N-terminal prohormone of brain natriuretic peptide

Multiple studies reported elevations of cardiac biomarkers during and after the completion of a marathon, suggesting myocardial damage (see Fig. 1). Elevations in cardiac troponin-I, creatine kinase (CK) and creatine kinase myocardial band (CK-MB), myoglobin, atrial natriuretic peptide (ANP) and N-terminal prohormone of brain natriuretic peptide (NT-proBNP) have been observed and are described in Tables 1 and 2 [3, 42, 68, 84–92].

Apparently, exercise duration seems to influence cardiac biomarker elevation. Significantly higher levels of biomarkers of myocyte necrosis and cardiac congestion in participants completing an ultra-marathon were found compared to marathon finishers [84]. There seems to be a correlation between running preparation and an increase in cardiac biomarkers. Recreational athletes training less than 35 miles/week before a marathon had higher proven cardiac troponins as well as NT-proBNP [93]. In these participants, elevation of cardiac troponins and NT-proBNP was significantly associated with post-race diastolic dysfunction [93, 94]. Nevertheless, in most marathon participants, cardiac biomarkers were only transiently elevated. High-sensitive cardiac troponin T (hs-cTnT) reached peak levels at 24 h post-race and returned to normal levels within 72 h post-race (see Table 2 and Fig. 1). This dynamic was also followed by other biomarkers such as NT-proBNP, interleukin (IL) 6, IL-10, tumor necrosis factor α (TNFα) and cystatin C [85]. It is as yet unclear whether these changes in cardiac biomarkers arise from cardiomyocyte necrosis, ischemia or altered cardiomyocyte metabolism [68, 85]. However, considering the dynamics of hs-cTnT elevation, the biomarker increase is most likely caused by transient strain or altered myocyte metabolism instead of irreversible cardiac myocyte necrosis or ischemia [3, 68, 85].

#### Arrhythmias in Marathon Runners

In addition to studies on elevations in cardiac biomarkers, there is evidence that marathon runners are more likely to develop atrial fibrillation (AF) [95]. Male marathon runners were found to have an 8.8-fold higher risk of developing lone AF compared to a sedentary control group (see Table 2) [96]. An animal study on rats showed myocardial fibrosis, especially involving the RV and right atria (RA) after extensive endurance training, suggesting a potential source for ventricular arrhythmias and AF [97]. Lifetime training hours seem to play a critical role in left atrial (LA) enlargement [98, 99]. In fact, higher lifetime training hours are associated with prolonged P-wave duration indicating altered cardiac conduction or LA enlargement [99]. Furthermore, male marathon runners seem to be more at risk of atrial remodeling compared to female athletes, as prolonged P-wave durations and greater LA volume in male endurance athletes compared to female endurance athletes were observed [95]. These findings are in line with current knowledge, as LA enlargement is an established risk factor for the occurrence of AF [98]. A recent cardiac magnetic resonance imaging (MRI) study (see Table 2) identified non-ischemic fibrotic areas in up to 50% of male veteran endurance athletes [100, 101]. The prevalence of myocardial fibrosis correlated with years spent training and finished competitive marathons or ultramarathons [101]. Veteran athletes with fibrosis showed a greater burden of premature ventricular beats, as well as a higher prevalence of ventricular couplets and triplets compared to veteran runners without fibrosis [100]. These fibrotic areas might be the source of ventricular arrhythmias [100]. Whether veteran runners with fibrosis have a higher risk for malignant arrhythmic events is not entirely clear, yet, and needs further investigation [100].

#### Ventricular Dysfunction

Endurance exercise leads to a physiological adaptation and remodeling of the heart to meet the demands placed on the cardiovascular system during a marathon [3, 76]. Still, the question of impaired cardiac function frequently arises, particularly considering cardiac biomarker elevation. Already in 1987, Douglas et al. introduced the concept of “exercise-induced cardiac fatigue”. This is defined as temporary systolic and diastolic ventricular dysfunction after exercise [71, 102]. However, a recent study showed negligible effects of marathon running on biventricular systolic function [71]. In contrast, impaired diastolic function, predominantly affecting the RV, has been reported (see Table 2) [3, 43, 71, 92, 93, 103]. Post-race RV enlargement as well as impaired diastolic function of the RV are revealed as prolonged relaxation times can be shown both in echo and cardiac MRI studies [43, 92, 93, 103–105]. In this context, increased pulmonary pressures have been measured [93]. Accordingly, enlargement of the RA has been noticed [43, 68]. Nevertheless, observed alterations in cardiac function normalized to pre-race levels within 24 h [3]. Interestingly, the increases in pro-inflammatory cytokines, such as TNFα and IL-12p70, were shown to correlate with the extent of RV dysfunction [105] (see Table 2). MRI studies found contradictory results. Some discovered no myocardial necrosis or other signs of structural abnormalities despite significant elevations of cardiac biomarkers and evidence of ventricular dysfunction [63] whereas others found delayed gadolinium enhancement in 12.9% of athletes localized in the interventricular septum, predominantly affecting the RV [92] (see Table 2). In this respect, exercise-induced RV dysfunction has been shown to set the stage for complex arrythmias, sometimes rendering the need for an implantable cardioverter-defibrillator (ICD) [104]. However, possible long-term effects and adverse remodeling, especially of the RV, remain equivocal and need further investigation.

#### Cardiorenal Axis

Intense physical exercise, such as marathon running, imposes significant stress on both renal and cardiovascular systems, linking to transient cardiorenal dysfunction [45]. This finding is supported by elevated levels of catecholamines [106] and neurohormones during a marathon such as aldosterone, vasopressin, and ANP [107–109]. Elevated levels of NT-proBNP, hs-cTnT, CK-MB, creatinine and cystatin C [45, 85], are commonly observed suggesting cardiac strain, ventricular dysfunction [105], and reduced kidney function or AKI [45].

While the exact mechanisms behind exercise-induced cardiorenal syndrome remain unclear, a similar pathophysiology to cardiorenal syndrome in patients suffering from congestive heart failure seems plausible [110, 111]. The increased cardiac demand due to marathon running activates the renin–angiotensin–aldosterone system (RAAS) to regulate blood pressure and fluid balance [112]. RAAS activation and the high intensity of exercise shift blood flow towards skeletal muscle, reducing renal perfusion, potentially causing hypoxia and tubular damage that can lead to AKI [46]. Responding to exercise-induced dehydration, the kidneys conserve sodium and water, amplifying stress on the cardiorenal axis, and potentially resulting in an acute cardiorenal syndrome [45, 111]. Despite initial AKI findings in marathon runners, studies have shown inconsistent associations between AKI markers and indicators of cardiac strain such as NT-proBNP and CK, thus questioning the presence of acute cardiorenal syndrome [85, 113].

Generally, these effects resolve within 24–72 h post-exercise, with both cardiac and renal markers returning to baseline as fluid balance and normal blood flow are restored [85, 113]. However, repetitive high-intensity exercise without adequate recovery may promote cumulative effects on the cardiorenal axis, potentially leading to long-term impact on renal and cardiovascular health [45].

#### Coronary Artery Disease and Sudden Cardiac Death

Regular physical activity has beneficial effects in preventing CAD [3]. However, marathon runners are not immune to CAD [114]. Möhlenkamp et al. assessed the prevalence of coronary artery calcification (CAC) among healthy male marathon runners aged ≥ 50 years with prior marathon experience (≥ 5 completed marathon races during the previous three years). Framingham risk score (FRS) was lower compared to age-matched controls; however, CAC was more frequent in marathon runners. Distribution and calcification sites were similar between both groups. In total, 36% of marathon runners showed a CAC score ≥ 100 [44, 69]. This is highly relevant since CAD is reported to be the main cause of major cardiac events during marathon competitions [9, 68]. Similarly, a different study showed the adjusted risk of a CAC score ≥ 100 in individuals with very high levels of physical activity to be 11% greater compared to those with lower levels [115]. Nevertheless, no signs for increased all-cause mortality in this group were found despite higher CAC scores [115] (see Table 2). Fortunately, major cardiac events, especially SCD, are rare events during the competition. The current incidence of major cardiac events and SCD is reported at 2.33 per 100,000 and 1.01–1.67 per 100,000, respectively [9, 116], with male runners being more affected [116] (see Table 2). Almost half of SCD occurs in the final 1.6 km of the race, representing less than 5% of the whole race distance [3, 68]. Different etiologies have been debated regarding SCD. CAD and acute myocardial infarction are the dominating causes in over 40-year-old marathon runners, whereas in young athletes, mainly genetic causes such as hypertrophic or arrhythmic cardiomyopathy are more frequent [9, 68, 116–118]. So far, few risk factors for SCD during long-endurance events have been established, some of them being male and participating in a marathon compared to a half-marathon [3, 9, 68]. Interestingly, most runners suffering from SCD during an event have already participated in several long-endurance events and did not report any symptoms [9, 68, 69, 119]. This, of course, poses great risks, so pre-race low-dose aspirin usage is debated in persons at risk. Pre-race low-dose aspirin i.e., one single 100 mg dose of aspirin [120], might be especially valuable in the context of a systemic inflammatory response and general hypercoagulable state (see Table 3) induced by long-endurance exercise itself [119]. However, life-threatening events remain rare although serious and could be avoided by improving participants’ and trainers’ education on clinical symptoms [9].

**Table 3:** Overview of key findings from studies investigating effects of marathon training and running on the hematological system

Subjects	Intervention/Observation	Outcome	References
N = 7,820 Female = 4,229	Regular sports activities decrease the risk of venous thrombosis	Regular strenuous exercise led to a risk reduction for venous thrombosis OR 0.78 (95% CI 0.67–0.90) Sport activities with a higher injury risk had a less beneficial effect on thrombotic risk reduction with OR 0.93 (95% CI 0.69–1.26)	[269]
N = 34 Female = 7 Age 38 ± 11 years	Effects of marathon running on biochemical and hematological markers	The white blood cell count after a marathon rose significantly by 2.7-fold, platelet count rose by 1.1-fold, aPTT decreased by 1.2-fold, D-dimers increased by 2.4-fold, LDH increased by 1.9-fold No changes in hemoglobin levels were observed	[48]
N = 34 Female = 10 Age 36.8 ± 7.6 years	Changes of inflammation in non-professional marathon running	After the marathon race, leukocytes increased by 2.6-fold, erythrocytes by 1.1-fold, hemoglobin by 1.1-fold Lymphocytes decreased by 2.7-fold and neutrophils increased by 1.4-fold No change in Hct was observed All biomarker alterations returned to baseline levels after 2–7 days of recovery	[243]
N = 81 Female = 0 Age 37 ± 2.1 years	Association between hematological parameters and iron metabolism response after a marathon race	After the marathon, no changes in erythrocytes, Hb, Hct, MCV and MCH was observed Iron increased by 1.1-fold, ferritin by 1.3-fold immediately after the race Erythropoietin increased 1.3-fold after 24 h post-race Erythrocyte and Hct remained low compared to baseline for 15 days after the race suggesting an incomplete hematological recovery	[254]
N = 15 Female = 1 Age 42.9 ± 8.0 years	Effect of marathon and ultra-marathon on inflammation and iron homeostasis	Immediately after the marathon, leukocytes increased by 2.4-fold, neutrophils by 3.5-fold, monocytes by twofold, lymphocytes decreased by 1.35-fold. These values returned to baseline levels within 5 days Serum iron increased by 1.4-fold, transferrin by 1.1-fold, TSAT by 1.3-fold and ferritin by 1.3-fold After 5 days, iron and TSAT deceased by 1.2-fold, whereas ferritin remained elevated No significant changes in Hb, MCV and MCH could be observed Markers of iron homeostasis showed different response patterns with regard to running distance	[16]
N = 90 Female = 0 Age 32.5 ± 8.6 years	Hematological changes associated with marathon running	Immediately after the marathon race, erythrocytes increased by 1.04-fold, leukocytes by 3.6-fold, neutrophils by 5.5-fold, lymphocytes by 1.3-fold, and platelets by 1.6-fold No significant changes in Hb, MCV, MCH or Hct could be observed	[272]
N = 170 Female = 43 Age 41 ± 9 years	Iron homeostasis in recreational marathon runners	Iron depletion was found in 1.6% of male and in 28.0% of female athletes Functional iron deficiency was present in 3.9% of male and 25.5% of female runners Body iron stores calculated by TSAT/ferritin ratio were significantly higher in male athletes compared to female runners Iron overload was found in 15.0% of male but only in 4.7% of female runners	[276]
N = 85 Female = 85	Effect of iron and folate therapy on maximal exercise performance in female marathon runners with iron and folate deficiency	Of the 85 female athletes, 16% had iron deficiency but only 2% had iron deficiency anemia Low folate level was observed in 33% and 15% had MCV greater than 95 fl One week after treatment with oral folate or iron, folate and ferritin levels normalized, but no changes in maximum oxygen uptake, maximum treadmill running time, peak blood lactate levels and the running speed at the “blood lactate turnpoint” were observed	[277]
N = 14 Female = 2	Coagulation factor changes following endurance exercise	Immediately after the race, no changes in Hct could be observed Platelet count increased by 1.3-fold Activity of FVIII increased by 3.1-fold and vWF:Ag activity increased by 3.3-fold Significant elevation of β-Thromboglobulin up to 24 h after the race	[273]
N = 13 Female = 1	Blood coagulation activation and fibrinolysis during a downhill marathon run	During and after the race, no changes in Hb, MCV or MCH could be observed Immediately after the race, PT decreased by 1.1-fold, aPTT by 1.2-fold and Protein C and S were decreased after the run by 1.2-fold and 1.4-fold, respectively and remained decreased up to 24 h post-race Platelets increased by 1.3-fold immediately after the race Significant post-race elevations of TAT by 2.3-fold, PT 1 + 2 by 1.4-fold, PAP by 5.8-fold, PAI-1 by twofold, t-PA by 4.3-fold and D-dimers by 1.3-fold	[284]
N = 41 Female = 17 Age 37 ± 8 years	Effect of air travel on exercise-induced coagulatory and fibrinolytic activation in marathon runners	TAT increased more in travel subjects (2.6-fold) compared to nontravel subjects (1.5-fold increase) T-PA increase was similar in travel (4.6-fold) and nontravel group (4.9-fold)	[283]
N = 76 Female = 24 Age 43.4 ± 10.7 years	Compression socks and the effects on coagulation and fibrinolytic activation during marathon running	Compression socks significantly reduced the post-exercise increase in D-dimer (+ 9.02 ng/mL) compared to the control group (+ 25.48 ng/mL) Activation of coagulation and fibrinolysis was observed in all participants; however compression socks reduced fibrinolytic activity	[290]
N = 1 Female = 1	Protective effect of compression socks in a marathon runner with factor V Leiden	Compression socks during a marathon appeared to lower the impact on hemostasis as well as clot formation in this athlete as t-PA (-56%), TAT (-63%) and D-dimer (-30%) were significantly lower compared to the marathon without compression socks	[293]
N = 99 Female = 22 Age 49 ± 6 years	Marathon running increases circulating endothelial- and thrombocyte-derived microparticles	Immediately after the race, leukocytes increased by 2.5-fold, monocytes by twofold, platelets by 1.2-fold and lymphocytes decreased by 21% Endothelial-derived microparticles and thrombocyte-derived microparticles increased by 23% and 38%, respectively and returned to baseline levels within two days post-race	[264]

*aPTT* activated partial thromboplastin time, *FVIII* factor VIII, *Hb* hemoglobin, *Hct* hematocrit, *LDH* lactate dehydrogenase, *MCH* mean corpuscular hemoglobin, *MCV* mean corpuscular volume, *OR* odds ratio, *PAI* plasminogen-activator-inhibitor-1 antigen, *PAP* plasmin–a_2_-antiplasmin complex, *PT* prothrombin time, *PT 1* + *2* prothrombin fragment 1 + 2, *TAT* thrombin–antithrombin complex, *t-PA* tissue-type plasminogen activator antigen, *TSAT* transferrin saturation, *vWF*:Ag von Willebrand factor Antigen, *95% CI* 95% confidence interval

#### Modulation of Autonomic Nervous System

Several studies investigated the effects of marathon training on the ANS and sympathovagal balance [32, 71, 79, 81, 121–123]. ANS function seems to be altered by endurance exercise since low resting heart rate, first-degree AV block and other ECG changes are common findings in endurance athletes [79]. Nevertheless, increased vagal tone is associated with higher occurrence of premature atrial contractions, which may act as a trigger for paroxysmal AF [99]. Furthermore, paroxysmal AF may be promoted by increased parasympathetic activity alone due to the reduction of the refractory period [124]. Heart rate variability (HRV) is a further indicator of ANS activity and is mainly dependent on vagal modulation [125]. Reduction of HRV as a sign of decreased vagal activity towards the heart can be observed in several cardiac as well as non-cardiac diseases such as myocardial infarction and diabetic neuropathy [125]. After intense training and completion of a marathon reduced HRV could be measured, suggesting a shift towards sympathetic activity or a reduced parasympathetic tone, which returned to pre-race values in a time-dependent manner [121, 122].

#### Risks and Benefits

The cardiovascular system in the context of marathon training and running has been extensively studied. Regular physical exercise has a beneficial effect on the cardiovascular system in various domains, including the improvement of cardiovascular risk factors such as improved lipid profile, lower resting arterial blood pressure, improved endothelial function and decreased QT variability. However, marathon running is also associated with massive stress on the cardiovascular system, with elevations of cardiac biomarkers, increased incidence of AF, structural remodeling of the RV, cardiorenal syndrome, and major cardiac events.

However, much remains unknown. In particular, the long-term effects of marathon running on cardiac function, in terms of volume changes, atrial and ventricular remodeling, and ejection fraction, remain unclear. The impact of marathon running on systolic and diastolic cardiac function in the acute setting also continues to be ambiguous and needs clarification through larger-scale studies. Changes and dynamics of cardiovascular biomarkers, as well as the association between marathon running and the occurrence of CAC, have been well-studied in male cohorts. Nonetheless, data on female marathon runners remain sparse and points to a need for studies comparing male and female marathon runners.

### Respiratory Tract

Alongside the cardiovascular system, the respiratory system is exposed to great stress during marathon running, e.g., a significant increase in minute ventilation (V̇_E_) [39]. However, it has not received the same scientific attention as the cardiovascular system to date [12]. Nevertheless, endurance exercise such as marathon training and running affects the respiratory system in both positive and negative ways [12]. In this context, we identified 20 studies investigating the positive and negative effects of marathon training and running on the respiratory system and its function. The key findings of said studies are summarized in Table 4.

**Table 4:** Overview of key findings from studies investigating effects of marathon training and running on the respiratory system and its function

Subjects	Intervention/Observation	Outcome	References
N = 40 Female = 7 Age (yrs) nonelite runners 43.2 ± 2.6, controls 43.8 ± 20.6	Airway responsiveness in non-asthmatic nonelite marathon runners compared to sedentary controls	Significantly higher FEV1 (by 1.1-fold), FVC (1.1-fold), and TLC (by 1.1-fold) in runners Diminished relative (by 2.3-fold) and absolute (1.9-fold) IVC decrease in runners after single-dose methacholine test Decreased FEV1 (by 1.1-fold) and FVC (1.1-fold) immediately after the race with normalization after 1 h post-race	[127]
N = 9 Female = 3 Age (yrs) 48.6 ± 9.4	Pulmonary and respiratory muscle function elite runners after 10 marathons in 10 consecutive days	Significant reduction in FVC at day 4, remaining below baseline at day 7 and 10 Decrease in FEV1 on day 1, 7, and 10 after the marathon FEV1/FVC ratio increased after day 1 and 7 compared to baseline values Reduction in MEP after day 1, 7, and 10, and no significant reduction in PEF or MIP 56% of runners reported symptoms of URTI in the 15-day post-challenge period	[50]
N = 9 Female = 0 Age (yrs) 32 ± 7	Changes in respiratory muscle and lung function after a marathon in experienced runners	Significant decrease in MIP (by 1.2-fold) and PIF (1.3-fold), normalization of values within 24 h post-race No significant changes in MEP, PEF, FEV1, and FVC	[130]
N = 11 Female = 1 Age (yrs) 32.9 ± 2.3	Changes of lung volumes during a treadmill marathon	Significant reduction of FVC after 120 and 150 min of the run Significant increase of RV after 150 min, without any significant changes in TLC during the exercise	[131]
N = 8 Female = 0 Age (yrs) 37.9 ± 2.9	Changes in lung function and DL_CO_ after a marathon	Significant increase in FEV1 (by 1.03-fold) and FEV1/FVC ratio (by 1.04-fold) after the race, with normalization a day later No changes in PEF, RV and TLC compared to controls Significant decrease in DL_CO_ by 1.1-fold post-race, with normalization within a day	[133]
N = 34 Female = 0 Age (yrs) 35.0 ± 3.4	Respiratory muscle fatigue after a marathon	No significant changes in FEV1 and FVC Significant decrease in MIP by 1.2-fold and MEP by 1.4-fold after the race	[134]
N = 13 Female = 2 Age (yrs) 33.1 ± 8.9	Changes in pulmonary function after a marathon	Significant decrease in FVC (by 1.1-fold) and FEV1/FVC ratio (by 1.1-fold) as well as significant increase in RV (by 1.2-fold), with normalization after 24 h post-race No significant changes in FEV1 and TLC	[132]
N = 2	Particulate matter deposition in the respiratory system of a recreational and elite marathon runner	Total PM10 deposition is 22% higher in recreational runners compared to elite runners during a marathon Breathing air at rest containing 50 µg/m^3^ PM10 results in a PM10 deposition rate of µg/h Running a marathon with V̇_E_ = 62 l∙min^−1^ (recreational runners) increases deposition rate by fivefold up to 45 µg/h and with V̇_E_ = 115 l∙min^−1^ (elite runners) by 9.2-fold up to 83 µg/h	[39]
N = 139 Female = 16 Age (yrs) 39 ± 9	Allergy in marathon runners	Prevalence of asthma in runners was 4.3%, allergic rhinitis 17.3%, allergic conjunctivitis 2.9%, food allergy 5.0%, atopic eczema 4.3%, and any allergy 26.6% Phatiotop® test was positive in	[136]
N = 201 Female = 36 Age (yrs) 34 (95% CI 22–49)	Allergy in elite marathon runners using Allergy Questionnaire for Athletes (AQUA©)	60.7% showed a positive AQUA score (≥ 5), 39.3% showed a negative AQUA score (< 5) AQUA© has a high specific (97.1%) but a low sensitivity (58.3%) Allergic rhinitis was reported by 58.3%	[139]
N = 208 Female = 81 Age (yrs) male runners 40.3 ± 10.9, female runners 37.4 ± 9	Allergy and upper respiratory tract symptoms in marathon runners	Prevalence of physician-diagnosed allergic diseases in runners: asthma (24.8%), eczema (8.0%), drugs allergy (7.0%), rhinitis (3.4%), food allergy (1.5%) 65.7% had a positive AQUA score (≥ 5), 49.3% had a positive serum IgE response for at least one selected allergen Allergic diseases were reported by 31% 32% reported symptoms in terms of exercise-induced bronchoconstriction	[135]

*AQUA©* Allergy Questionnaire for Athletes, *DL*_*CO*_ diffusing capacity of the lung for carbon monoxide, *FEV1* forced expiratory volume in 1 s, *FVC* forced vital capacity, *IVC* inspiratory vital capacity, *MEP* maximum expiratory mouth pressure, *MIP* maximum inspiratory mouth pressure, *PEF* peak expiratory flow, *PIF* peak inspiratory flow, *PM* particulate matter, *PM10* particles with a diameter of ≤ 10 μm, *RV* residual volume, *TLC* total lung capacity, *URTI* upper respiratory tract infection, *V̇*_*E*_ minute ventilation, *95% CI* 95% confidence interval

#### Improvement in Lung Function

Marathon running poses a great challenge to the respiratory system as adequate ventilation and blood oxygenation are crucial for athlete’s peak performance. In this way, V̇_E_ in recreational athletes increases by 7–8 times up to 62 l∙min^−1^, while in elite athletes, V̇_E_ reaches values up to 115 l∙min^−1^, corresponding to a 15-fold increase compared to rest [39, 126]. Lung function is significantly better in marathon runners compared to sedentary controls [127]. Significantly higher volumes of forced expiratory volume in 1 s (FEV1), forced vital capacity (FVC), functional residual capacity, as well as higher total lung capacity (TLC), were found in nonelite marathon runners compared to sedentary controls (see Table 4) [127]. Additionally, recreational runners could improve their lung function, i.e., FVC and FEV1 over a 2-year training period [128]. Even older runners (range 40–75 years) could inhibit or decelerate the age-related decrease in pulmonary function, thus improving general health and quality of life [128]. Furthermore, endurance training seems to mitigate the decline in lung volumes following bronchoprovocation (see Table 4), as suggested by a significantly smaller reduction in lung volumes in recreational marathon runners compared to controls during a single-dose methacholine test [127]. These results suggest a low airway responsiveness best explained by higher baseline lung volumes [127]. Patients suffering from cystic fibrosis might benefit from physical exercise [129]. There are reports of cystic fibrosis patients successfully finishing a marathon race without any adverse events, suggesting marathon running to be safe for this patient population and promising for a positive effect on the course of the disease [129].

#### Effect of Intense Exercise on Lung Volumes and Function

Acute bouts of intense exercise such as a marathon pose immense stress on the respiratory system and may harmfully alter lung function [12]. Several studies measured lung function and volumes using spirometry or plethysmography after a marathon [12]. There are contradictory results regarding changes in lung function after a marathon race (see Table 4). However, most studies found diminished values of FVC immediately after a race [12, 50, 127, 130–132]. Some studies, on the other hand, reported no changes in FVC after completion of a marathon [130, 133, 134]. Nevertheless, FVC reduction usually resolves within 24 h after a race [12, 50, 127, 130–132]. Similarly, ambiguous results have been observed for FEV1. Some studies reported no changes in FEV1 [130, 132, 134], whereas others documented increases [50, 133] or even decreases in FEV1 [127, 131]. Therefore, different results in changes in the FEV1/FVC ratio have been observed. Some studies reported an increased FEV1/FVC ratio with a return to pre-race values within 24 h after the race [132, 133], whereas others observed no changes in the FEV1/FVC ratio [50, 134]. Hypotheses suggest small airway obstruction as a cause of restricted lung function after marathon racing, as field studies have demonstrated reduced FEV within 1–2 s and decreased MEF at 50% of FVC [12, 132]. However, these hypotheses are still equivocally debated because of mixed results. Studies investigating static lung volumes i.e., TLC and residual volume (RV), found varying results. Most studies found no significant changes in TLV and RV [131–133]. However, one reported a significant increase in RV and a concomitant increase in RV/TLC ratio immediately after a marathon with a return to baseline after 24 h post-race [132]. Interestingly, a recent study conducted on marathon runners completing 10 marathons in 10 consecutive days found similar results as described, but no cumulative or chronic changes in respiratory function or dyspnea were reported, highlighting the exceptional adaptive abilities of the respiratory system to enormous physical strain [50].

Additionally, some studies assessed the diffusing capacity of the lung for carbon monoxide (DL_CO_) with different results (see Table 4). Some studies found decreased values for DL_CO_ after a marathon race suggestive of subclinical pulmonary edema [12, 133], whereas other studies found no significant changes in DL_CO_ [132]. In any case, DL_CO_ values returned to pre-race levels within a day after the race [12, 133].

#### Respiratory Muscle Fatigue

Another common problem apart from changes in lung volumes is respiratory muscle fatigue [12]. Similar to post-marathon fatigue of leg muscles (see Table 5), both inspiratory and expiratory muscles are impaired in their function to generate force compared to baseline capacity [12]. Usually, respiratory muscle strength is adequately measured using mouth-pressure maneuvers [12]. Studies found contradictory results regarding respiratory fatigue (see Table 4). A reduction of maximum expiratory mouth pressure (MEP) but no changes in peak expiratory flow (PEF) was found by some studies [50], in contrast to an isolated decrease in PEF [134], while others found no changes in expiratory function [127, 130]. Similar results regarding inspiratory respiratory muscle fatigue have been observed. Some studies observed a significant decrease in maximum inspiratory mouth pressure (MIP) and peak inspiratory flow (PIF) [130, 134], whereas others reported no changes in MIP [50]. All in all, ambiguous results regarding inspiratory and expiratory muscle fatigue are present. Nonetheless, current literature suggests a tendency to inspiratory muscle fatigue after a marathon race and expiratory muscle fatigue after an ultra-marathon race [12]. Taken together, changes in pulmonary function after a marathon race are most likely explained by a combination of airway obstruction, restriction, as well as respiratory muscle fatigue [12].

**Table 5:** Overview of key findings from studies investigating effects of marathon training and running on the musculoskeletal system and its function

Subjects	Intervention/Observation	Outcome	References
N = 42 Female = 42 Age 37.7 ± 0.8 (range 30–49) years	Role of exercise in prevention of involutional bone loss in marathon running pre-menopausal women	In runners, BMD was significantly greater by 1.05-fold and 1.1-fold at midshaft radius and the middle phalanx of the fifth digit, usual sites of osteoporotic fractures BMD at os calcis was higher by 1.07-fold in the sedentary control group	[205]
N = 31 Female = 11 Age 40.5 ± 9.8 years	Effects of endurance running on plasma osteoprotegerin (OPG) and soluble receptor activator of NF-κB ligand (sRANKL)	After a marathon race, serum OPG levels increased significantly by 1.8-fold and sRANKL levels decreased significantly by 1.6-fold	[207]
N = 298 Female = 0 Age 40.5 ± 9.8 years	Comparison of calcaneus bone stiffness between endurance runners of different ages and age-matched controls using ultrasonographic measurements	With higher age calcaneus bone stiffness decreased independent from physical activity Calcaneus stiffness was significantly higher in 40–44, 45–49 and > 50-year-old marathon runners compared to their age-matched sedentary controls by 1.09-fold, 1.02-fold and 1.04-fold, respectively	[208]
N = 1,002 Female = 518 Age 44.4 ± 12.7 years	Effects of marathon training and running on bone health assessed by calcaneal broadband ultrasound attenuation	Men had significantly higher BMD compared to the women independent of physical activity Marathoners had significantly higher BMD compared to sedentary controls in both men by 1.1-fold and women by 1.2-fold A significant decline of BMD occurred with increasing age for all groups	[210]
N = 82 Female = 45 Age 44 ± 7.8 years	Impact of marathon training and running on knee damage of middle-aged adults	MRI images were obtained 6 months pre-race and half a month after the race Pre-race images showed signs of damage to several knee structures in majority of runners, yet all asymptomatic Post-race images showed a reduction in radiological damage scores in subchondral bone marrow edema in tibia condyles and femur An increase in damage scores was found in the cartilage of the lateral patella, semimembranosus tendon, iliotibial band and the prepatellar bursa	[211]
N = 27 Female = 10 Age 29.4 ± 3.5 years	Improved skeletal muscle oxygen consumption (muscle VO_2_) after endurance training using near-infrared spectroscopy measurements	After 16 weeks of endurance training in previously non-athletic adults, muscle VO_2_ increased by 48% suggesting high metabolic adaptation of skeletal muscle due to endurance training	[214]
N = 11 Female = 6 Age 16 ± 1 years	Musculotendinous changes during a 6-month endurance training assessed via ultrasound measurements	A significant increase in thickness of medial gastrocnemius muscle, tibialis anterior muscle and Achilles tendon by 1.1-fold, 1.2-fold, and 1.1-fold, respectively was found Echogenicity decreased significantly in all muscles and muscle fiber pennation angles significantly increased suggesting favorable musculotendinous adaptations for endurance running	[216]
N = 8 Female = 0 Age 37 years (mean)	Knee MRI before and after a marathon race	Runners underwent an MRI of the knee before, immediately after and 6–8 weeks after a marathon race No negative effects were experienced in 75% of runners without major pre-existing lesions In one runner with pre-existing high-grade meniscal lesions, signs of progressive osteoarthritis were experienced after 2 months post-race In all other participants increased meniscal signals and minor bone marrow signal changes were observable, yet all were transitory and asymptomatic	[221]
N = 675 Female = 283 Age 47.9 ± 11.6 years (range 18 to 79)	Prevalence of hip and knee arthritis in active marathon runners	Marathoners which participated in ≥ 5 marathons and were running a minimum of 10 miles per week were included in this study A total of 47% reported hip or knee pain and 8.8% of runners reported osteoarthritis Osteoarthritis prevalence was significantly lower in marathon runners by twofold compared to subgroups stratified by age, sex, body mass index, and physical activity level with reported osteoarthritis prevalence of 17.9%	[15]
N = 3,500	Main running-related musculoskeletal injuries	This systematic review found most common running-related injuries to be medial tibial stress syndrome (with an incidence of 13.6–20.0%), Achilles tendinopathy (incidence ranging from 9.1–10.9%) and plantar fasciitis (with an incidence of 4.5–10.0%) Other common running-related injuries were patellar tendinopathy, ankle sprain, iliotibial band syndrome, hamstring muscle injury and bone stress injuries e.g., tibial stress fracture	[226]
N = 720 Female = 500 Age 35.9 ± 9.4 years	Risk factors for injuries in first-time marathoners	In first-time marathon runners 9.5% had major injuries during training or the race 49.2% had minor injuries during training or the race Injury incidence was not significantly different based on age or sex Runners who previously completed a half-marathon were less likely to getting injured with OR 0.4 (95% CI 0.22–0.76) Runners with ≥ 4 training runs per week were at higher risk for injury compared to those which had < 4 runs per week with RR 1.36 (95% CI 1.13–1.63) Longest training run distance was inversely associated with an injury on race-day (OR 0.87, 95% CI 0.81–0.94)	[227]
N = 28 Female = 14 Age 32.4 ± 8.6 years	Impact on hip MRI of first-time marathoners	MRI was performed 16 weeks before and 2 weeks after the marathon In the pre-race images, MRI abnormalities of the hop joint were seen in 90% of runners After the race only 4.7% of hips showed new findings (mild bone marrow edema)	[233]
N = 10 Female = 5 Age 39.9 ± 3.8 years	Effect of a six-month training program and a consecutive marathon on knee joint cartilage volume and thickness assessed by MRI in asymptomatic inexperienced runners	Lateral femur cartilage volume significantly decreased by 3.2 ± 3.0% and thickness decreased by 1.7 ± 1.6% Non-significant changes were observed at medial and lateral tibia, medial femur, and the patella	[235]
N = 28 Female = 14 Age 30 (median with range 18–58) years	Effects of marathon training and a consecutive race on lower lumber spine in first-time marathoners	Lumbar spine of asymptomatic first-time marathoners was assessed using MRI 16 weeks before and 2 weeks after the marathon The participants took part in a 16-week training program In pre-race images disc degeneration was detected in 61% of participants, mostly at spinal segments L4-L5 and L5-S1 though asymptomatic After the marathon, no significant change in disc degeneration progression or intervertebral disc height was found	[238]
N = 139 Female = 45	Ultrasound evaluation of Achilles and patellar tendon and its association with future pain in marathon runners	Prior to the marathon, tendon abnormalities were found in 24.1% of the Achilles and in 23.1% of the patellar tendons Runners with ultrasound abnormalities were significantly more likely to develop pain within 12 months after the race with a HR of 2.89 (95% CI 1.05–7.93) and 2.73 (95% CI 1.11–6.78) for Achilles tendon and patellar tendon respectively	[241]
N = 35 Female = 0 Age 39 ± 8 years	Influence of marathon running on musculoskeletal biomarkers	CK values were measured 2 weeks before the marathon race, immediately after the race and 2 weeks post-marathon CK levels significantly increased immediately after marathon by 2.7-fold and returned to baseline values within 2 weeks	[245]
N = 15 Female = 1 Age 42.9 ± 8.0 years	Effect of marathon and ultra-marathon on inflammation and iron homeostasis	Immediately after the marathon, CK values increased by 3.0-fold and were still increasing by 4.2-fold 5 days post-race compared to baseline values LDH increased significantly by 1.8-fold immediately after the race and started to return to baseline values 5 days post-race The same biomarkers showed even more pronounced elevations in ultra-marathon finishers	[16]
N = 98 Female = 15 Age 38.7 ± 3.6 years	Changes in serum biomarkers of runners suffering from muscle cramps during a marathon race	In total, 24% of finishers experienced muscle cramps during or immediately after the race There were no differences in changes of body mass and post-race serum sodium and potassium levels Runners suffering from muscle cramps showed significantly greater post-race CK and LDH values by 1.2-fold and 1.2-fold, respectively compared to non-crampers After 24-h post-race CK and LDH values were also higher in crampers compared to non-crampers by 2.1-fold and 1.2-fold, respectively	[255]
N = 35 Female = 0 Age 40.8 ± 8.8 years	The effect of muscle strength on marathon race-induced muscle soreness	Isokinetic strength tests and V̇O2max levels was performed 15–30 days pre-race All participants reported more pain 24-h after the race compared to pre-race values Knee extensor strength values were significantly associated with higher pain 24-h post-race but not 48 or 72-h post-race V̇O2max levels were not associated with pain levels after the race	[258]

*BMD* bone mineral density, *CK* creatine kinase, *DXA* x-ray densitometry, *HR* hazard ratio, muscle, *V̇O2max* skeletal muscle oxygen consumption, *LDH* lactate dehydrogenase, *MRI* magnetic resonance imaging, *OPG* osteoprotegerin, *OR* odds ratio, *RR* relative risk, *sRANKL* soluble receptor activator of NF-κB ligand, *95% CI* 95% confidence interval

#### Asthma and Atopy in Marathon Runners

Despite positive effects of endurance training on lung volumes and airway responsiveness, a higher prevalence of asthma (see Table 4) in endurance athletes such as marathon runners compared to the general population has been observed [126]. Asthma prevalence in endurance runners was reported between 4.3 and 24.8% [135–137]. There are different hypotheses trying to explain this phenomenon. First of all, the airways of athletes are exposed to hyperventilation which in turn leads to airway drying and cooling inducing mild inflammation, delayed bronchial epithelial repair and stimulation of the vagal nerve, which in turn leads to airway drying and cooling, inducing mild inflammation, delayed bronchial epithelial repair, and stimulation of the vagal nerve, which in turn leads to mild bronchoconstriction [126, 138]. Additionally, hyperventilation leads to a larger amount of inhaled allergens and irritants, which can promote asthmatic symptoms [126]. Marathon races usually take place in large cities with varying air quality [39]. A recent study showed increased deposition rates of particulate matter (PM), such as particles with a diameter of ≤ 10 μm (PM10), in the respiratory tract of runners during a marathon race (see Table 4), which was especially more pronounced in recreational athletes compared to elite runners [39]. Furthermore, endurance athletes exhibit a higher prevalence of allergy and atopy, as reported by several studies. Atopy in endurance runners significantly increases the risk of asthma by up to 42-fold compared to non-atopic athletes [136]. The reported prevalence of allergy in endurance runners is significantly higher than in the general population and ranges between 10 and 60% [135, 137, 139], the prevalence of atopy was reported between 31 and 65% [126, 135, 136], the prevalence of allergic rhinitis lay between 7.1–56.6% [136, 137, 139], and 31–49% showed a positive Immunoglobulin (Ig) E response for one or more selected allergens (see Table 4) [135, 136]. In this regard, some authors suspect typical upper respiratory tract infections (URTIs) after a marathon to be of allergic or autoimmune origin rather than infectious [135, 137] (see Table 6), as in only 30% of post-race URTIs, an infectious pathogen could be detected [135]. Nevertheless, a study investigating marathon runners with mild airway obstruction showed moderate-to-high levels of performance despite pulmonary dysfunction, suggesting only a minor impact of airway obstruction on actual race performance [140].

**Table 6:** Overview of key findings from studies investigating effects of marathon training and running on the immune system and its function

Subjects	Intervention/Observation	Outcome	References
N = 12 Female = 0	Reduction of plasma concentration of CRP following nine months of endurance training	During nine months of marathon training there was a marked decrease in baseline CRP values The median fell by 1.5-fold from initially 1.19 mg/l to 0.82 mg/l after the training period	[315]
N = 15 Female = 1 Age 42.9 ± 8.0 years	Effect of marathon and ultra-marathon on inflammation	After marathon and ultra-marathon, a significant increase in CRP levels could be observed by 1.2-fold and 6.2-fold, respectively In the marathon group, CRP values were still increasing, and elevated 5-days post-race 1.5-fold compared to baseline levels Ultra-Marathon CRP values after 5-days were still elevated but in a decreasing fashion	[16]
N = 102 Female = 0 Age 42 ± 9 years	72-h kinetics of inflammatory biomarkers after marathon	Immediately after the race, IL-6, IL-10 and TNFα were significantly elevated by 15.5-fold, 5.6-fold, and 1.1-fold, respectively The values for hs-CRP were 21.4-fold increased after 24-h post-race All the measured values returned to baseline levels after 3 days post-race	[85]
N = 20 Female = 0 Age 29.6 (IQR 24.3–37.2) years	Differential time responses in inflammatory stress markers after a marathon	The plasma concentrations of TNFα, IL-6 and IL-10 were increased by 45%, 43-fold and 40-fold immediately after the race No changes in CRP concentrations were observed immediately after the race After 4 days post-race there was a CRP increase by 654% All biomarkers returned to normal values within 9 days post-race	[294]
N = 12 Female = 0	Acute phase response after a marathon race and the effects of glutamine supplementation	Immediately after the race, there was 1.1-fold increase of TNFα, but no increase in CRP was found After 16 h post-race, there was a 1.3-fold increase of IL-2 and a 4.5-fold increase in CRP Leukocytes immediately increased by threefold and lymphocytes decreased by 1.7-fold No observable changes in cytokine or leukocytes between the placebo and the glutamine supplementation group	[295]
N = 8 Female = 0 Age 32.9 ± 14.3 years	Acute changes in inflammatory biomarkers in recreational marathoners and half-marathoners	Immediately after the race, there was significant elevations in IL-6, IL-8, and IL-10 but no changes in TNFα could be observed In the marathon running group there was a significantly higher increase in IL-8 and IL-10 compared to the half-marathon group Biomarker changes returned to normal after 48-h post-race	[296]
N = 2,311 Female = 347 Age 36.9 ± 0.2 years	Infectious episodes in runners before and after a marathon	Runners with higher training mileage per week (≥ 97 km/week) compared to lower mileage (< 32 km/week) had an increased risk for infectious episodes during the 2 months prior to the marathon, namely OR 2.0 (95% CI 1.2–3.4) Comparing runners who finished the marathon with similar runners who did not participate in the race, 12.9% of the finishers reported symptoms of URTI during the first week post-race, whereas only 2.2% of the control group reported URTI symptoms There is an increased risk for URTI after completion of a marathon, namely OR 5.9 (95% CI 1.9–18.8)	[53]
N = 22 Female = 0 Age 41.4 ± 9.4 years	Cytokine kinetics in nasal mucosa and sera	Immediately after the marathon race, there was a significant decrease in salivary IgA by 1.6-fold with return to baseline levels after 72-h after the race Concentration of IL-6 and IL-10 was elevated directly after the race in nasal mucosa extract IL-6 was higher in symptomatic runners, whereas IL-10 was higher in asymptomatic runners even before the race which might be due to the anti-inflammatory effects of IL-10	[304]
N = 16 Female = 5 Age 41 ± 2.6 years	Effects of strenuous exercise on Th1/Th2 gene expression of marathon participants	After the marathon race, there was a trend of down-regulation of two Th1 related genes which persisted for 1 week after the race The findings suggest a Th1/Th2 immune imbalance which explains the increased risk for URTIs after intense strenuous exercise	[309]
N = 42	Endurance exercise diverts the balance between Th17 cells and regulatory T cells	Immediately after the race, there was 2.6-fold increase in leukocytes Lymphocytes decreased by 1.1-fold Absolute Th17 cell counts increased fourfold after the race, but the percentage of Treg decreased significantly post-race by threefold and have not yet recovered after 10 days post-race	[137]

*CRP* C-reactive protein, *hs-CRP* high-sensitivity C-reactive protein, *IgA* immunoglobulin A, *IL* interleukin, *IQR* interquartile range, *OR* odds ratio, *Th* T helper cell, *TNFα* tumor necrosis factor α, *Treg* regulatory T cell, *URTI* upper respiratory tract infections, *95% CI* 95% confidence interval

Rare cases of very serious adverse events, such as noncardiogenic pulmonary edema regarding the respiratory system, have also been described after marathon running [141, 142]. Typically, noncardiogenic pulmonary edema is observed in the context of severe exercise-induced hyponatremia, often accompanied by cerebral edema [141, 142]. Most likely, a disturbance in the blood-gas barrier, e.g., by exercise-induced increased pulmonary capillary wedge pressure, is the cause of noncardiogenic pulmonary edema, although, the pathogenesis is not entirely understood yet [142].

#### Risks and Benefits

In conclusion, marathon training and running pose both benefits and risks for the respiratory tract. Particularly, regular physical exercise such as marathon training seems to improve lung function and helps to prevent deterioration of age-related decline in lung function.

On the other hand, negative effects of marathon running on respiratory function have been observed. Especially after a marathon, lung function is temporarily reduced due to a combination of obstruction, restriction, and muscle fatigue [12]. Additionally, marathon runners seem to have an increased risk for asthma, allergy, and atopic diseases.

The respiratory system has not received the same research interest and popularity as the cardiovascular system and many aspects of how endurance exercise influences the respiratory system are not yet entirely clear, especially the mechanisms of airway obstruction, restrictive components, and respiratory muscle fatigue. The inconclusive results acquired by different studies regarding changes in lung function after a marathon race are also worth mentioning. Further studies with larger study populations are required and should investigate potential mechanisms leading to small airway obstruction as well as possible mediators of allergy and asthma to provide athletes with the best possible support.

Altogether, marathon training and running are generally safe for the respiratory system particularly considering that most changes occur only transiently.

### Renal System

Impairment of renal function during marathon races is quite often observed [45]. We identified 29 studies investigating the impacts of marathon training and running on the renal system as well as on electrolyte and fluid balance. Table 7 gives an overview of the most important findings from said studies.

**Table 7:** Overview of key findings from studies investigating effects of marathon training and running on the renal system

Subjects	Intervention/Observation	Outcome	References
N = 256 Female = 47 Age 61.0 ± 12.2 years	Physical activity and change in estimated GFR in chronic kidney disease (CKD) patients	Mean baseline eGFR was 42 ml/min/1.73 m^2^ among the whole study cohort Over the course of median 3.7 years follow-up the mean change in eGFR measured by serum cystatin C was -7.6% (IQR − 16.4 to 4.9%) per year Participants who reported > 150 min of exercise per week had the lowest rate of eGFR loss with a mean decline of 6.2% compared to inactive controls with a decline of 9.6% per year Each 60-min increment in weekly exercise duration was associated with a 0.5% (95% CI 0.02–0.98) slower eGFR decline per year	[143]
N = 33 Female = 13 Age 43.7 ± 10.3 years	Effects of acute exercise on renal function in non-dialysis CKD patients compared to healthy individuals	All CKD patients had 1.3–1.5-fold lower peak oxygen uptake and reached 1.1–1.3-fold lower maximal heart rate values compared to the control group suggesting significantly lower cardiorespiratory fitness No significant within-group differences in serum creatinine, urinary creatinine excretion rate and changes in albuminuria across time in any of the groups	[11]
N = 25 Female = 13 Age 38.7 ± 9.0 years	Dynamics of serum creatinine and cystatin after a marathon race in relation to volume status	40% of runners showed a rise of serum creatinine and cystatin c meeting criteria for AKI in the absence of volume depletion Biomarkers returned to baseline levels within 24 h	[45]
N = 22 Female = 13 Age 44.2 ± 12.9 years	Dynamics of serum creatinine as well as urine microscopy	82% of runners met criteria for AKI after the race 73% of participants showed microscopic evidence of acute tubular injury Serum creatinine levels remained increased up to 2 days post-race	[46]
N = 23 Female = 13 Age 37 (IQR 35–44) years	Role of sodium and volume balance on AKI during marathon races	55% of runners developed AKI and 74% showed signs of acute tubular injury in urine microscopy Higher sweat sodium losses in runners with AKI and correspondingly higher copeptin levels	[47]
N = 134 Female = 36	Relationship between NSAID intake during marathon race and serum biomarker alterations	Serum creatinine and potassium levels were significantly higher in marathon runners with NSAID use	[154]
N = 568	Rise in serum creatinine levels after marathon races as marker of AKI	Mean increase of serum creatinine levels by 25.7 (± 11.6) µmol/l Significantly higher increase of serum creatinine in collapsed runners or runners using NSAIDs	[112]

*AKI* acute kidney injury, *CKD* chronic kidney disease, *eGFR* estimated glomerular filtration rate, *IQR* interquartile range, *NSAID* non-steroidal anti-inflammatory drugs, *95% CI* 95% confidence interval

#### Significance of Exercise in Chronic Kidney Disease Patients

Physical exercise is an important factor in the therapeutic approach of chronic kidney disease (CKD) patients [11]. It seems that regular moderate physical activity, such as marathon training, slows disease progression in CKD patients (see Table 7) as physically active CKD patients have better renal function compared to inactive controls [143, 144]. In fact, CKD patients who exercised > 150 min/week had a 1.5-fold slower estimated glomerular filtration rate (eGFR) decline per week compared to sedentary controls [143]. Vigorous exercise may have acute adverse effects on renal function. However, moderate exercise is safe for patients with CKD, and its benefits exceed possible harms [11]. In addition to delaying disease progression, exercise improves cardiorespiratory fitness as well as many comorbidities associated with CKD, such as arterial hypertension, obesity and diabetes, which ultimately reduces all-cause mortality and significantly improves the quality of life in CKD patients [11, 143–146].

#### Acute Kidney Injury

Nevertheless, a marathon puts immense stress on the renal system as the prevalence of AKI meeting the Acute Kidney Injury Network (AKIN) definition based on a rise in serum creatinine levels is reported to be from 40% up to 82% in marathon participants [45, 46, 147]. Microscopic urine analysis allowed diagnosis of acute tubular injury in ~ 75% of runners, which most likely embodies the main cause of AKI in endurance exercise [46, 47]. A third of marathon participants develop microscopic hematuria [148]. Of marathon runners, 50% developed significant hematuria without AKI [149]. Moreover, a study on marathoners reported large increases in urinary biomarkers of cell cycle arrest and renal stress, suggesting that marathon running can be associated with kidney stress and potential injury [150].

The etiology of AKI during long-endurance events is not entirely conclusive. Several different mechanisms of AKI are currently debated. First of all, dehydration due to extensive fluid losses or inadequate fluid replacement can lead to pre-renal AKI [45, 47, 112]. In this regard, a recent study investigating the influence of volume regulation in AKI during marathon races found a significant correlation between higher sweat volume losses and runners developing AKI [47]. This effect may be amplified by an extensive shift of blood volume from internal organs towards muscles [46]. At rest, kidneys receive ~ 20% of cardiac output, which may decrease by up to 25% under strenuous exercise, suggesting causation of ischemic tubular damage and transient reduction of kidney function [46, 151]. Furthermore, exertional rhabdomyolysis leading to the release of extensive amounts of myoglobin, resulting in pigment nephropathy with concomitant AKI, has been described [112, 152, 153].

The use of NSAID during exercise has been shown to decrease blood flow to the kidneys [112]. Mean change in elevation of serum creatinine as well as urea has been shown to be increased by 9.1 and 8.9%, respectively, in marathon competitors using NSAID during the competition (see Table 7) compared to regular runners [154]. Therefore, NSAIDs might act as a risk factor for the occurrence of AKI during marathon competitions and should be avoided [112].

In addition, exercise-induced release of catecholamines, antidiuretic hormone (ADH), and activation of the RAAS may impair kidney function and reduce GFR by up to 50% [112].

**Fig. 2:**
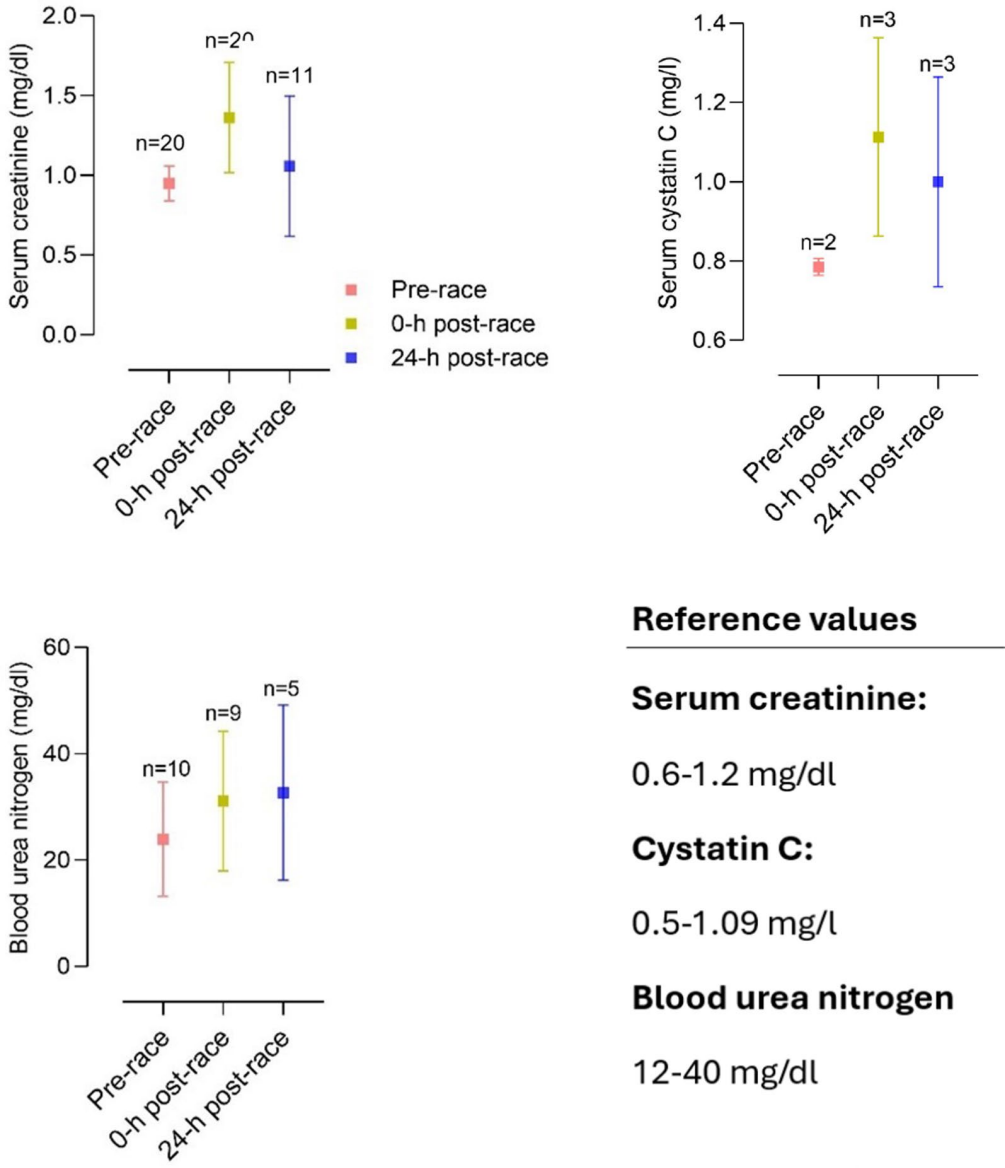
Mean changes in serum creatinine, cystatin C, and blood urea nitrogen in studies investigating renal biomarkers before and after a marathon [42, 45–47, 59, 84–86, 89, 106, 148, 155, 156, 156–158, 195, 196, 199, 243, 244, 254]

Several studies reported elevated kidney biomarkers (see Table 1, Table 7, and Fig. 2) during and after marathon races [45–47, 112, 154–157]. In general, an average rise in serum creatinine levels by 29 µmol/l or approximately by 40% with an accompanying decrease in glomerular filtration rate (GFR) can be observed [112, 155]. Usually, serum creatinine levels normalize within 24 h post-race [157]. Still, a post-race decline in GFR has been reported by several studies [46, 158].

Creatinine plays an important role in muscle metabolism especially during exercise which might distort measurement of kidney function based on serum creatinine. Nonetheless, concomitant increases up to 26% in serum levels of cystatin C, which is a muscle-independent marker of kidney function, have been observed (see Table 7 and Fig. 2), proving kidney injury to some extent [45, 46, 112, 157]. All biomarker elevations were resolved within 2 weeks and no signs of persisting impaired renal function were reported [112, 157]. Recent studies showed elevations of new blood and urine biomarkers of tubular damage, such as neutrophil gelatinase-associated lipocalin (NGAL) and kidney injury molecule-1 (KIM-1), which returned to normal values within 24 h post-race [45, 46, 112, 155, 157]. The best recovery from AKI is achieved in an active manner by light-intensity continuous running from 48 h post-race [158].

#### Exercise-Associated Hyponatremia

**Fig. 3:**
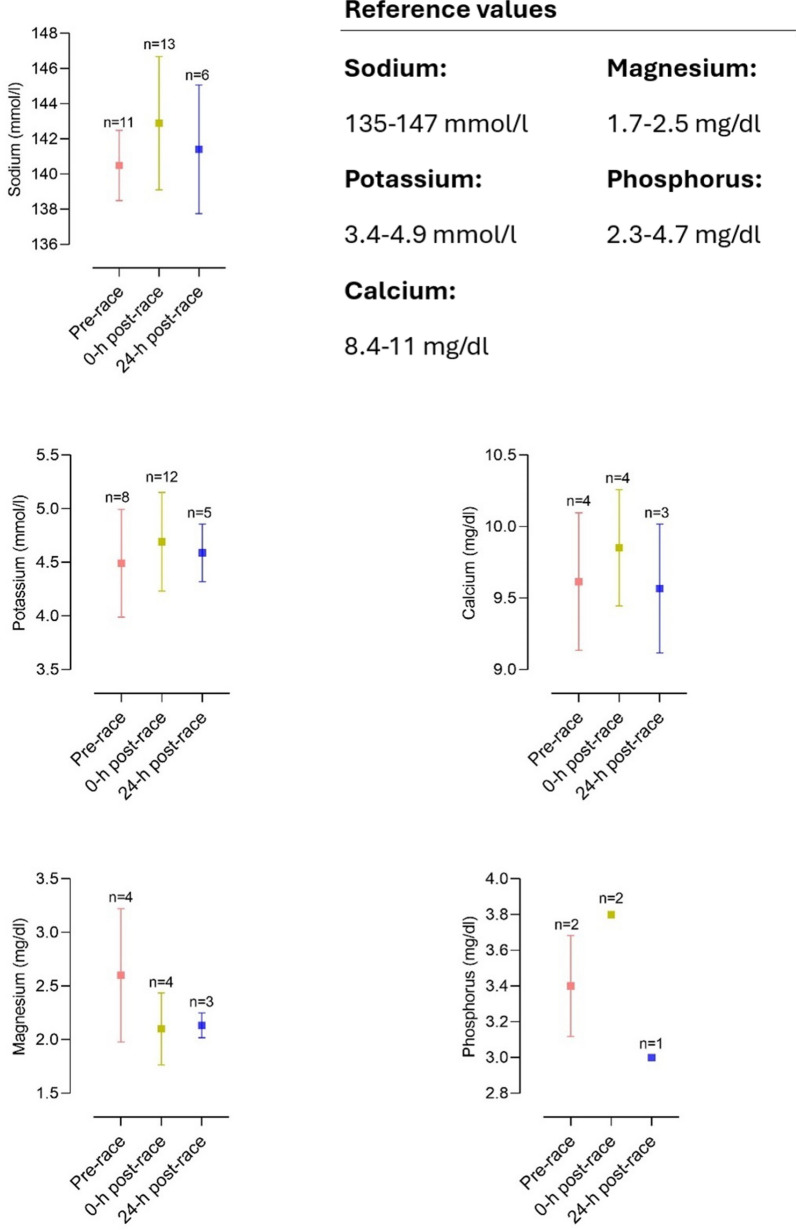
Mean changes in serum sodium, potassium, calcium, magnesium, and phosphorus in studies investigating electrolytes before and after a marathon [42, 45, 47, 59, 89, 106, 156, 243, 244, 255, 271, 271, 314]

Fluid and electrolyte imbalances are commonly seen during long-distance running competitions (see Table 1 and Fig. 3) [49, 59]. There is a wide variation in the incidence of electrolyte disturbances in the literature due to environmental factors such as weather conditions (e.g., heat). All in all, sodium seems to be the most affected electrolyte, especially with plasma volume changes [49, 156, 159]. Particularly, exercise-associated hyponatremia (EAH) is of great clinical significance as it occurs frequently in marathon athletes, with reported prevalence being 1 and 20% [49]. Typically, endurance runners suffering from EAH present either asymptomatically or with nonspecific symptoms, including nausea, emesis, headache, and confusion [49, 141, 160]. Nevertheless, in rare cases, EAH can cause severe complications such as noncardiogenic pulmonary edema, seizures, cerebral edema, coma, and death [49, 141, 160, 161]. A retrospective study found significantly elevated post-race plasma sodium levels compared to baseline in marathoners, with only 15% of readings outside the normal range in non-collapsed runners. However, no significant difference in plasma sodium changes was observed between collapsed and non-collapsed runners [147]. EAH is most likely caused by multiple different factors; in particular, excess fluid intake during the race associated with a reduced decrease in body mass index (BMI) promotes EAH [49]. Moreover, stress directly exerted by intense exercise and the release of proinflammatory cytokines in response to exertional rhabdomyolysis stimulate the release of ADH, leading to the syndrome of inappropriate antidiuretic hormone secretion (SIADH) [161]. Some risk factors have been established, particularly overdrinking, body-weight gain, female sex, use of NSAIDs, as well as event-related risk factors such as high temperatures contribute to EAH [49]. Important for the prevention of EAH is regular sodium substitution (~ 1–2 g/h) in the form of electrolyte-containing fluids as well as an adequate drinking strategy (~ 500–1000 ml/h), which must be individually adapted and implemented into each athlete’s training program [49].

#### Risk and Benefits

Kidney function during a marathon has been diligently researched. Regular strenuous exercise has several beneficial effects on the renal system such as slower disease progression as well as better quality of life in CKD patients. However, marathon running exerts an extreme stress on the renal system and AKI, acute tubular necrosis, as well as fluid and electrolyte imbalances are quite often observed. Much remains unclear, though. In particular, the long-term implications of marathon running and repeated episodes of AKI on renal function and the occurrence of CKD remain unclear and need further investigation.

### Gastrointestinal System

The digestive tract plays an essential role in long-distance running as it absorbs and provides valuable nutrients required for peak performance [13, 36]. Nevertheless, it is a rather delicate system as endurance athletes commonly report GI symptoms during or after marathon races [51]. We identified 30 studies studying the impact of marathon training and running on the GI system.

#### Improvements in Gastrointestinal Health

Moderate exercise, such as marathon training has several beneficial effects on the GI system (see Table 8). Regular physical activity improves GI motility and can accelerate gut transit times, preventing large quantities of fecal residues and excess intraluminal gas [162–164]. This has positive effects on patients suffering from inflammatory bowel disease (IBD) or irritable bowel syndrome (IBS) and reduces the risk for diverticular disease as well as constipation [162, 163]. Furthermore, the risk for cholelithiasis is reduced in physically active men and women independently from other risk factors, such as obesity, by factors of up to 3.3 and 2.3, respectively [165, 166]. Regular physical activity, such as marathon training, has proven positive effects on the incidence of colorectal cancer. Physically active individuals have a 1.2–3.6-fold reduced risk of developing colorectal cancer compared to inactive controls [167, 168]. Similarly, regular physical activity reduces cancer-specific as well as overall mortality in patients suffering from colorectal cancer [169].

**Table 8:** Overview of key findings from studies investigating effects of marathon training and running on the GI system

Subjects	Intervention/Observation	Outcome	References
N = 8 Female = 3	Effects of physical activity on intestinal gas transit and evacuation	Intestinal gas retention was lower during exercise compared to rest Gut transit of intraluminal gas is improved by moderate physical activity	[164]
N = 430,584 Female = 229,359	Sedentary behavior and risk for colorectal cancer	Risk for colorectal cancer was lower (HR = 0.84, 95% CI 0.72–0.98) in physically active individuals with metabolic equivalent hours ≥ 60 per week compared to a control group with < 10 metabolic equivalent hours	[168]
N = 573 Female = 573	Influence of physical activity on survival after colorectal cancer diagnosis	Cancer-specific mortality as well as overall mortality were reduced in physically active women with a HR of 0.48 (95% CI 0.24–0.97) and 0.51 (95% CI 0.3–0.85), respectively	[169]
N = 96 Age 43.4 ± 9.5 years	Prevalence of gastrointestinal symptoms during a marathon in non-elite athletes and correlation with nutritional intake	47% of runners reported symptoms 7 days prior to the marathon and 27% reported GI symptoms during marathon. Most frequent symptoms were flatulence (16%) and nausea (8%). No statistically significant correlation between nutritional intake and occurrence of GI symptoms were observed	[51]
N = 114 Female = 31	Fluid intake and GI problems in a 25-km race and a marathon	GI symptoms were commonly reported. In the 25-km race 25% of runners and 52% in the marathon race complained about GI symptoms, most commonly stomach ache (25%), side ache (23%), intestinal cramps (18%) and nausea (11%) Dehydration was shown to be associated with the occurrence of GI symptoms as 80% of runners who lost > 4% of their body weight during the race experienced GI symptoms	[170]
N = 707 Female = 103 Age 35.5 years	Frequency of GI symptoms during or after a marathon race	Most common upper GI symptoms were heartburn (8.8–9.5%), nausea (1.8–11.6%) and vomiting (0.3–1.8%) Heartburn typically occurred during the race, whereas nausea was more common directly after the race Lower GI symptoms were abdominal cramps (10.9–19.3%), urge to have a bowel movement (36.4–38.6%) and diarrhea (8.2–10.0%) whereas urge to have a bowel movement and diarrhea occurred more frequently directly after the race Lower GI symptoms were significantly more common in female athletes	[52]
N = 26 Female = 3 Age 37 years	GI hormone changes after a marathon	After the race, increased plasma concentrations of gastrin (2.2-fold), VIP (6.2-fold), GLP-1 (2.3-fold), secretin (3.5-fold) and motilin (1.9-fold) were measured whereas insulin concentrations were significantly decreased by 4.5-fold. The mechanism for the release of these peptides is unclear; however, reduced splanchnic blood flow or metabolic demands are the most likely causes There was no direct relationship between the increase of hormonal levels and the occurrence of GI symptoms	[173]
N = 36 Female = 36	Effects of marathon training and running on bowel function, GI blood loss and iron status in women	Marathon running compared to sedentary controls resulted in a 21% increase in gut transit time, a 27% decrease in daily stool weight and 21% lower frequency of defecation This study found no significant Hb loss after a marathon as well as no differences in iron status between the groups	[182]
N = 49 Female = 3	Fecal blood loss in response to walking or marathon running	There was no increase in fecal hemoglobin content in the walking group In marathon runners, fecal hemoglobin content increased by 0.42 mg/g feces (95% CI 0.12–0.83) The increase significantly correlated with race time, faster marathon finishers having higher amounts of occult blood as well as NSAID intake	[183]
N = 125 Female = 57 Age 38 ± 10 years	Incidence of GI blood loss after running a marathon	22.4% of marathon finishers with prior to the marathon negative Hemoccult test had positive test results after the marathon race, indicating that running the marathon was associated with a significant increase in the incidence of GI bleeding GI symptoms were reported by 24% of athletes, most common being abdominal cramps (16.8%), diarrhea (6.4%) and vomiting (0.8%)	[184]
N = 63 Female = 7 Age 40.8 years	Incidence of GI bleeding in marathon runners	Fecal blood was observed in 13% of marathon participants. Hematuria and proteinuria were present in 13% of runners and 35%, respectively. GI distress was reported by 54% of athletes, most frequent being diarrhea (42%), abdominal cramps (27%) and nausea/vomiting (20%)	[186]

*GI* gastrointestinal, *GLP-1* glucagon-like peptide-1, *Hb* hemoglobin, *HR* hazard ratio, *IQR* interquartile range, *NSAID* non-steroidal anti-inflammatory drugs, *VIP* vasoactive intestinal peptide, *95% CI* 95% confidence interval

#### Gastrointestinal Distress

High-intensity or long-distance exercise also seems to entail several negative effects on the GI system, as GI complaints are one of the most commonly reported symptoms during a marathon race [51, 170]. In studies, 20–57% of marathon runners complained about at least one symptom of GI distress (see Table 8), younger runners, as well as female athletes, are affected more frequently [52, 162, 163, 170]. Typically reported symptoms include nausea, vomiting, heartburn, loss of appetite, abdominal cramps, flatulence, bloating, urge to have a bowel movement and (bloody) diarrhea [51, 52, 163, 171]. To date, the etiology of said GI symptoms is not entirely conclusive, and different factors are being discussed. During exercise, blood volume is shifted away from the intestines towards the muscles. In this manner, splanchnic blood flow can be decreased by ~ 80% and gut mucosa becomes susceptible to ischemia [163, 172]. As a result, mucosa permeability is increased, which promotes translocation of microbiota, and endotoxins might be released, leading to diarrhea [52, 162, 163, 172]. Especially during running, there is an increase in vibration of the abdominal wall as well as bouncing of the intestines, which irritate the gut and may lead to GI symptoms [162, 163]. Several studies have observed increased levels of GI hormones (see Table 8) such as vasoactive intestinal polypeptide (VIP), gastrin, secretin, and glucagon-like peptide 1 (GLP-1) to such an extent as is otherwise only known in hormone-producing pancreatic tumors [173]. These hormone elevations may explain several GI symptoms such as loss of appetite and diarrhea, but there is currently no statistically significant correlation between GI hormonal changes and the occurrence of GI symptoms [163, 173].

Additionally, dehydration seems to be associated with GI disorders and leads to delayed gastric emptying [170, 172, 174]. A statistically significant correlation between dehydration and GI symptoms has been shown by Rehrer et al. [170]. In fact, 80% of runners who lost > 4% of their body weight complained about GI symptoms, mainly abdominal cramps [170]. GI problems can also result from relative absorption deficiency [13]. Absorption of nutrients, especially those of carbohydrates, is dependent on the density and activity of sodium-dependent glucose-1 transporters (SGLT1) in the intestines and under normal conditions limited to ~ 60 g/h. However, for an optimal marathon performance a carbohydrate intake of ~ 90 g/h is recommended, which may lead to GI distress, mainly bloating due to limited absorption [13]. A high-carbohydrate diet (70% of total dietary energy) 2 weeks prior to the marathon race results in a doubling of SGLT1 in the intestinal mucosa, which allows for better absorption of higher amounts of carbohydrates, reduces GI symptoms and potentially improves performance [13]. Additionally, carbohydrate loading (~ 10 g/kg body mass per day) 24–36 h prior to the race was shown to improve the ability to maintain a steady pace for longer and should be encouraged for races > 30 km [175]. Other studies reported dietary restrictions prior to the race to prevent GI distress as certain nutritional elements e.g., fat and protein, have been suggested to induce exercise-induced GI symptoms [176]. The most commonly omitted foods were milk products (rich in fat and protein), high protein, high-fiber, chocolate, and caffeine [176]. The most recent study on GI cell injury after running the Boston Marathon found that intestinal cell injury and GI symptoms decreased from the marathon finish to the day after the marathon and the reduction was influenced by hydration status [177].

#### Low Energy Intake

Low energy intake and relative energy deficiency in sport (REDs) occur when the energy intake is insufficient to meet the demands of training and basic physiological functions [178, 179]. This disproportion between caloric consumption and energy expenditure is common among endurance athletes such as marathon runners [179]. The prevalence of low energy intake and REDs is particularly high in female runners though comprehensive data on exact prevalence rates remain limited [179]. Generally, there is a lack of awareness regarding the energy demands of high-intensity training [179]. REDs has far-reaching effects on metabolism, cardiovascular health, and reproductive function, including chronic fatigue, weakened immune function, menstrual irregularities in women, decreased bone density, and impaired muscle recovery while also increasing injury risk and reducing athletic performance [179–181]. The overtraining syndrome shares similar symptoms to REDs so that shared pathways and an overlap syndrome between the two entities have been discussed [180]. Prevention strategies are an important tool, including implementing personalized nutrition plans and ensuring adequate recovery, and utilizing screening tools like the REDs Clinical Assessment Tool for early detection [178, 179].

#### Gastrointestinal Bleeding

Several studies observed occult GI blood loss associated with long-distance running (see Table 8) [182–186]. It is estimated that in ~ 8–30% of marathon runners occult bleeding is found in stool samples [187]. While the etiology is still unclear, ischemia due to reduced splanchnic blood flow, as well as direct mechanical trauma through bowel irritation, are the most plausible explanations [163, 184, 186]. GI bleeding is significantly associated with faster race times and less experienced runners [163, 183, 188]. In relation to GI bleeding, iron deficiency has been described, particularly in female athletes, as long-distance athletes have lower serum iron and ferritin levels as inactive controls [182, 183, 185–187]. GI blood loss in long-distance running is usually occult and transient, but there are reported cases with severe ischemic colitis, sometimes requiring surgical intervention [163, 187, 188].

#### Risks and Benefits

The GI system in relation to long-distance endurance sports enjoys great research interest. Benefits of regular physical exercise on the GI system include improved GI motility and transit times as well as reduced risk for diverticulitis, cholelithiasis and colorectal cancer. However, marathon running may lead to severe GI distress, delayed gastric emptying, as well as fecal blood loss with possible anemia.

Much remains unclear, especially regarding the causes of GI symptoms and bleeding. More research is needed to answer these questions. Athletes and coaches should be made aware of the importance of the GI system and especially incorporate effective food and fluid intake into their training program to prevent GI distress and improve absorption of nutrients.

### Hepatobiliary System

The liver is an essential organ for metabolism and plays a central role in providing energy supplies such as glucose and free fatty acids [189]. For this reason, this organ is heavily used and indispensable during long-distance events, even if active mainly in the background. Until recently, there has been limited research on the effects of marathon training and running on the liver. Nevertheless, in recent years, there has been a significant increase in research interest. Studies have shown new insights into how exercise positively affects the liver and patients suffering from liver disease [14]. However, marathon running also has negative influences on the liver. We identified 18 articles investigating both positive and negative effects of marathon running on the liver. The most relevant findings of said studies are summarized in Table 9.

**Table 9:** Overview of key findings from studies investigating effects of marathon training and running on the hepatobiliary system

Subjects	Intervention/Observation	Outcome	References
N = 1,530	Effects of endurance exercise in obese patients with fatty liver disease	Physical activity led to a marked reduction of intrahepatic fat by − 3.46% (95% CI − 5.20 to − 1.73%) as well as a reduction in fasting free fatty acids by − 74.15 µmol/L (95% CI − 118.47 to − 29.84)	[190]
		A significant reduction in insulin MD − 1.88 U/L (95% CI − 3.43 to − 0.34) could be observed	
		Liver enzymes were not significantly altered with exercise	
N = 777,662 Female = 427,714 Age 55 (IQR 51–62) years	Physical activity and the risk of hepatocellular carcinoma	Physical activity was associated with a significantly reduced risk for HCC with OR 0.65 (95% CI 0.45–0.95)	[192]
		Vigorous physical activity was associated with even higher risk reduction with OR 0.62 (95% CI 0.49–0.79)	
N = 10 Female = 2	Changes of liver enzymes after completion of a marathon and a half-marathon	AST and ALT showed significant post-race elevation with peak values at 48 h after the race	[196]
		Marathon runners showed markedly higher values of AST and ALT compared to half-marathon runners 48-h post-race	
N = 50 Female = 0 Age 51.76 ± 6.88 years	Comparison of changes in biochemical markers hepatic metabolism after three types of long-distance running	Marked increase after the marathon in ALT, AST and total bilirubin by 1.1-fold, 1.3-fold, and 1.1-fold, respectively	[195]
		Significantly higher increases in these parameters could be observed after the 100-km and 308-km race, suggesting intensity related mechanisms in elevations of hepatic metabolism	
N = 18 Female = 0 Age 36.05 ± 3.25 years	Effect of RIPC on liver and heart injury markers after a marathon	One group underwent RIPC of the legs for ten consecutive days before a marathon using occlusion cuffs inflated up to 220 mmHg while control group occlusion cuff was set to 20 mmHg. Color flow Doppler ultrasound was used to ensure full closure of arterial inflow to the legs	[201]
		Immediately after the marathon. The control group showed a significantly more pronounced increase in ALP, AST, GGT and total bilirubin levels by 1.1-fold, 1.2-fold, 1.5-fold, and 1.1-fold, respectively	
		Suggesting a decreased hepatic stress induced by RIPC before the marathon	

*ALP* alkaline phosphatase, *ALT* alanine aminotransferase, *AST* aspartate aminotransferase, *GGT* gamma-glutamyltransferase, *HCC* hepatocellular carcinoma, *OR* odds ratio, *RIPC* remote ischemic preconditioning, *95% CI* 95% confidence interval

#### Relevance of Exercise in Liver Disease

In many parts of the world, nonalcoholic fatty liver disease (NAFLD) is the most prevalent cause of liver disease and may lead to nonalcoholic steatohepatitis (NASH) or result in fibrosis and, ultimately in, end-stage liver disease [14]. Insulin resistance in adipose tissue, liver, and skeletal muscle plays a key role in the pathogenesis of NAFLD [14, 190]. Regular exercise, such as marathon training, was shown to improve both lipid and glucose metabolism, increase insulin sensitivity and to significantly reduce intrahepatic fat by 3.5%, as well as fasting free fatty acids, thus improving liver function (see Table 9) [190]. Furthermore, physical activity reduces oxidative stress in the liver and vascular endothelium and leads to generally improved cardiorespiratory fitness, which decreases inflammation and fibrosis progression in NAFLD patients [14]. Similarly, patients with a chronic hepatitis C infection undertaking a moderate exercise program showed significantly improved liver function [14]. Moderate exercise in patients with compensated cirrhosis resulted in improved maximal oxygen consumption (V̇O_2_max) values and led to a ≥ 10% decrease of hepatic venous pressure gradient (HVPG) in 42% of patients without changes in hepatic blood flow, thus improving portal hypertension and quality of life [14]. Acute exercise leads to a significant increase in portal pressure by 16–21% and a marked decrease in hepatic blood flow [191]. However, pretreatment with beta-blockers compensates for the HVPG increase caused by acute exercise, which makes physical activity safe for cirrhotic patients, and positive effects outweigh possible negative consequences in the long term [14]. A recent meta-analysis (see Table 9) investigating the risk of hepatocellular carcinoma (HCC) in physically active subjects found that greater amounts of physical activity compared to less physically active individuals had significantly lower odds of HCC with an odds ratio (OR) of 0.65 and vigorously physically active subjects had even lower odds of HCC with OR 0.63 [192]. Moreover, moderate-to-vigorous-intensity exercise (> 7 h/week) before HCC diagnosis was associated with reduced mortality (RR 0.71) compared to inactive controls [193]. Exercise seems to exert an anti-tumoral effect on HCC growth. A mouse model comparing sorafenib with sorafenib and moderate exercise in mice with HCC showed a significantly higher reduction in tumor volume in exercising mice compared to sedentary controls, highlighting the positive influence of physical activity on tumor growth and progression [194].

#### Markers of Hepatobiliary Damage

**Fig. 4:**
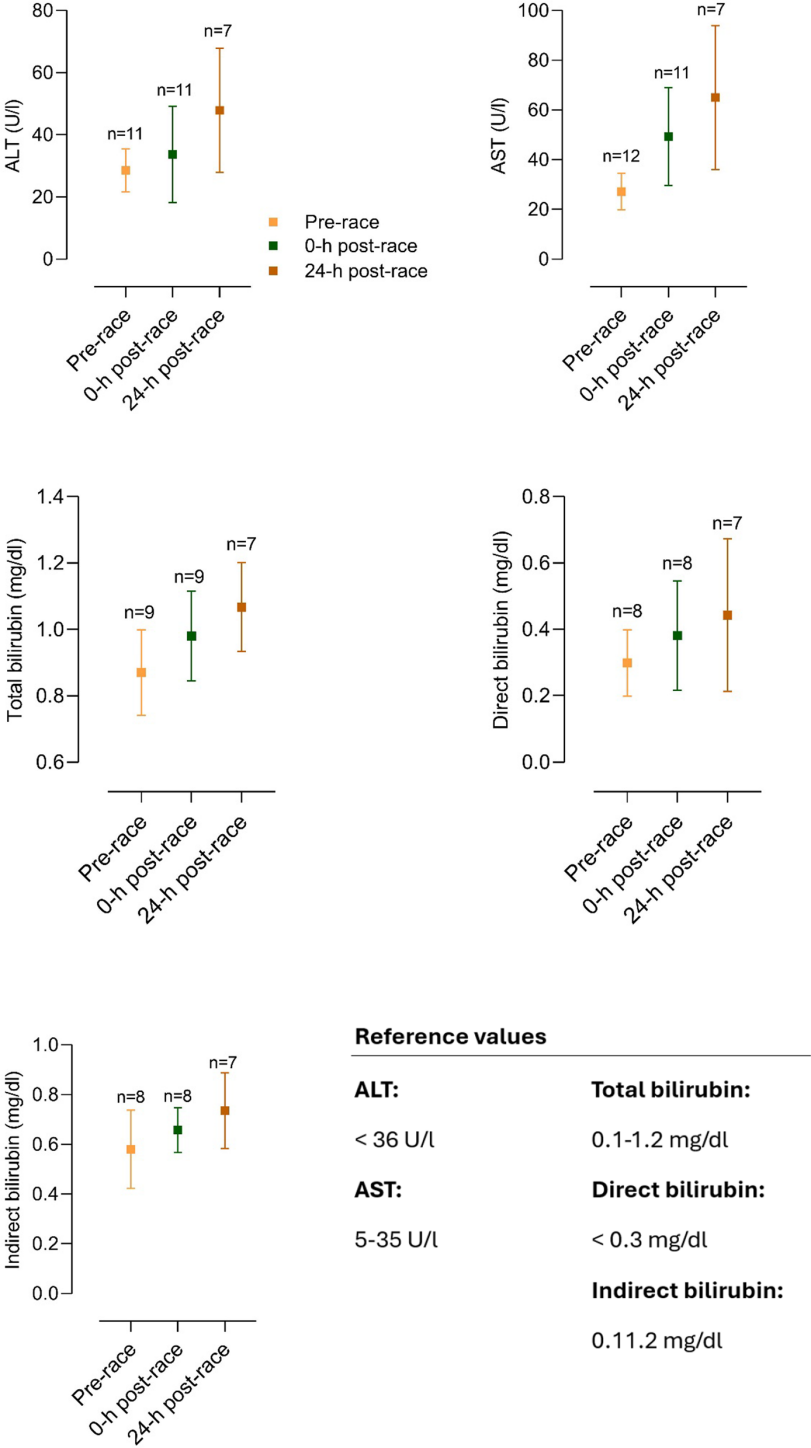
Mean changes in ALT, AST, total, direct, and indirect bilirubin in studies investigating hepatic and cholestasis biomarkers before and after a marathon [45, 59, 84, 106, 195, 196, 199, 246, 254, 316]. ALT, alanine transaminase; AST, aspartate transaminase

Negative effects of marathon running on the liver have been described, nonetheless. Several studies reported elevated liver biomarkers during or after the completion of a marathon even in adolescent runners [59, 195–199]. Significant post-race changes occurred in aspartate transaminase (AST) and alanine transaminase (ALT) with peak values at 48 h post-race (see Table 9 and Fig. 4) [196]. Running a marathon also leads to an increase in parameters of cholestasis, i.e., alkaline phosphatase (ALP), gamma-glutamyltransferase (GGT), and bilirubin which normally return to baseline levels within 24 h after the race (see Fig. 4) [197, 198]. These biomarker changes suggest exercise-induced damage to the liver, which seems to depend on the exercise intensity as elevations in liver enzymes as well as bilirubin were markedly higher after ultra-marathon events [195].

**Fig. 5:**
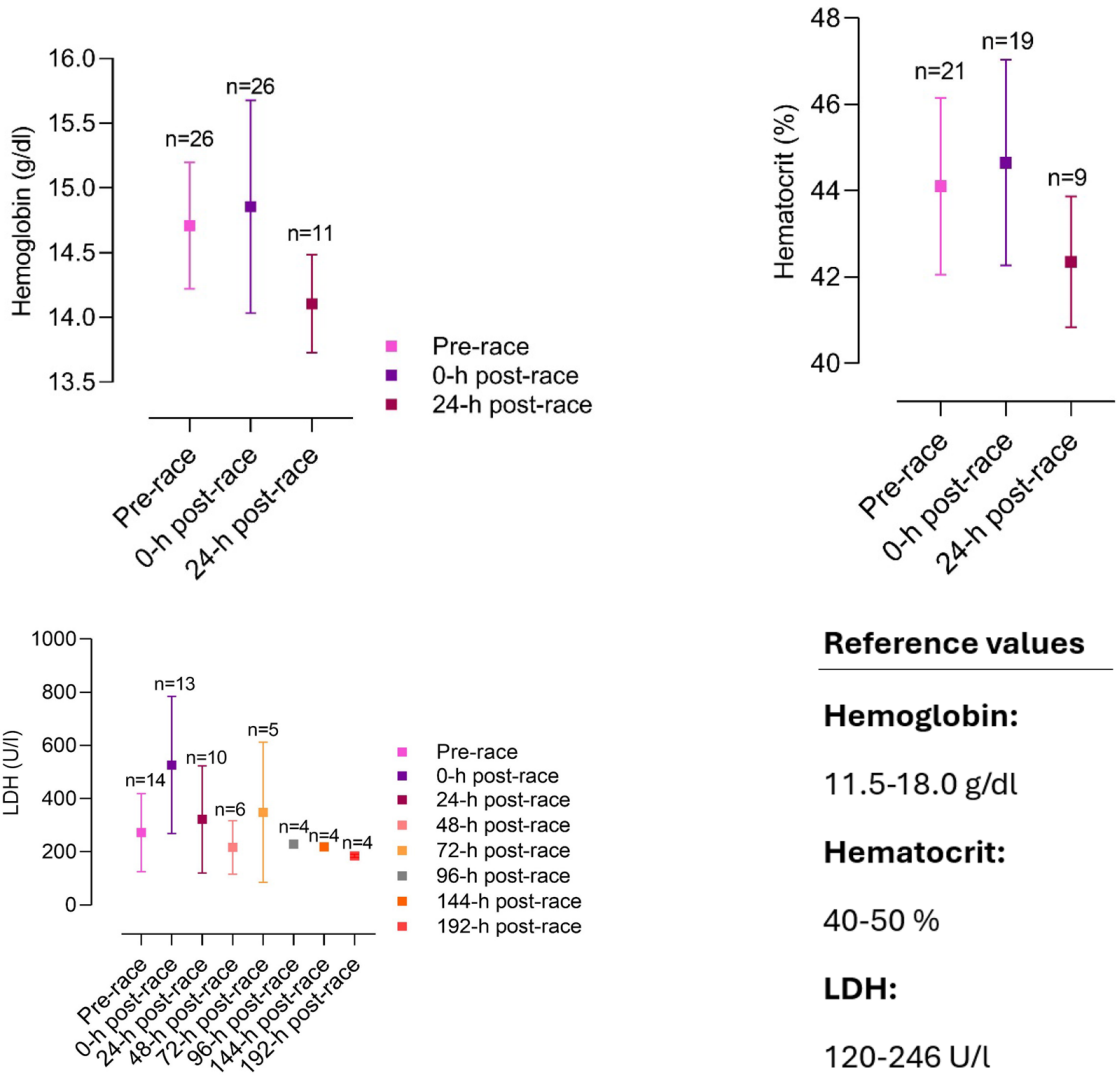
Mean changes in hemoglobin, hematocrit, and LDH in studies investigating hematological biomarkers before and after a marathon [16, 42, 47, 57, 59, 87–89, 102, 106–108, 148, 156, 195, 243, 244, 252, 254, 255, 260, 271–273, 286, 308, 311, 314, 316, 326, 333]. LDH, lactate dehydrogenase

The etiology of these biomarker elevations is not entirely clear and different possible mechanisms are currently being debated. AST and GGT are not liver-specific and also produced by muscle cells, which in turn may reflect muscle injury rather than hepatic involvement [197, 200]. The increase in bilirubin may be interpreted as a marker of transient hepatic insufficiency, but bilirubin elevations might also be explained by hemolysis (see Table 3 and Fig. 5) or increased red blood cell turnover in the muscles [201]. Elevations in liver enzymes might also be an expression of increased hepatocellular membrane permeability or altered metabolic demands as they play key roles in glucose and lipid metabolism, such as fatty acid oxidation and gluconeogenesis [196, 200]. Nevertheless, ALP and ALT are highly liver-specific parameters and underline hepatic damage to some extent, which might be due to ischemic factors or increased oxidative stress induced by free radicals generated by the high metabolic demands of muscles [195, 201]. Interestingly, remote ischemic preconditioning (RIPC) of leg muscles using cycles of short-lasting leg ischemia seems to reduce oxidative stress and decrease markers of liver and heart damage after a marathon (see Table 9), highlighting the impact of oxidative stress on liver function during the long-distance events [201].

#### Acute Liver Failure in Exercise

There are increasing numbers of reported cases of acute liver failure following a marathon [10, 202–204]. Most of these cases of acute liver failure were associated with EHS or extensive rhabdomyolysis, which in turn leads to a systemic inflammatory response with consecutive multiple organ failure [10, 202]. These patients were critically ill and often needed liver transplantation in ~ 30% of cases [202]. Some patients responded reasonably well to conservative treatment, but mortality in patients presenting with acute liver failure after a marathon must not be underestimated as it approaches nearly 33% [10, 202].

#### Risk and Benefits

Research interest in the effects of marathon training and running on liver function is quite recent. Regular physical activity was found to have a positive impact on NAFLD, lipid and glucose metabolism, decreased portal hypertension in cirrhotic patients and reduced the risk of HCC. Still, marathon running results in elevated liver enzymes and biomarkers of cholestasis and may lead to acute liver failure in the context of EHS.

Further efforts to investigate this relationship are needed. In particular, the effects of moderate exercise such as marathon training on liver transplant recipients and possible beneficial metabolic adaptations are not well understood. Furthermore, acute effects of exercise on HCC have been investigated. However, effects on long-term HCC-related outcomes are not extensively studied yet and may improve the quality of life in patients suffering from HCC.

### Musculoskeletal System

The musculoskeletal system arguably plays one of the most important roles during marathon training and running. During training and the race itself, enormous demands are placed on the musculoskeletal system, and it must be able to withstand immense stress [36]. Marathon training and running influence the musculoskeletal system to a great extent in both a positive (e.g., increased bone mineral density) and a negative way (e.g., running-related injuries) [15, 54]. The impact of endurance exercise on the function of the musculoskeletal system has been greatly researched over the past decades. In this context, we identified 66 studies examining the positive as well as harmful influences of marathon training and running on the musculoskeletal system. In Table 5, the key aspects of said studies are depicted.

#### Impact on Bone Health

Marathon training and running greatly impact the musculoskeletal system. Bone health is especially positively affected through regular moderate physical activity such as marathon training [205, 206]. Several studies showed that bone mass is increased by 2–3% by regular physical exercise [207]. This becomes particularly relevant with elderly individuals and/or postmenopausal women, in whom osteoporotic fractures and alterations are common leading to high morbidity and impairment of quality of life [205, 207, 208]. Endurance exercise probably influences the activity levels of two cytokines involved in bone metabolism, namely osteoprotegerin as well as soluble receptor activator of NF-κB ligand (sRANKL) (see Table 5) [207]. Osteoclasts are very potently activated by sRANKL, whereas osteoprotegerin competitively binds to sRANKL and blocks its function on osteoclasts, therefore inducing bone formation [207]. After a marathon race, osteoprotegerin is significantly increased in blood plasma and the activity of sRANKL significantly decreased which leads to a reduction of bone resorption and higher bone mass [207]. Bone mineral density is an accurate predictor of osteoporotic fractures [209]. The calcaneus has a relatively high metabolic activity and contains more than 90% trabecular bone, which is representative of total bone health [208, 210]. Several studies found ultrasonographic calcaneus bone stiffness to be significantly higher in marathon runners compared to sedentary controls, especially in participants older than 40 years (see Table 5) [208, 210]. This suggests a positive effect of marathon training on bone balance and health [208, 210]. There is probably a certain site-specific component in which higher stressed bones are more modified [209]. Dual-energy X-ray absorptiometry (DXA) scans of female runners showed higher bone mineral densities in legs but no changes in total body bone mineral density compared to sedentary controls [209]. Similarly, male runners showed higher lumbar spine bone mineral densities but did not display higher total body bone mineral density compared to age-matched sedentary controls [209]. Nevertheless, physical activity seems to have positive effects on bone health [207, 208, 210]. A recent MRI study investigating the effects of marathon running on the knee joint was able to demonstrate a reduction of subchondral bone marrow edema in the tibia condyles and femur (see Table 5) when comparing post-race to pre-race images [211]. Generally, the condition for this is, of course, that the energy needs of the runners are also met and that attention is paid to a sufficient supply of calcium (~ 1,000–1,500 mg/d) [212, 213].

#### Benefits on Skeletal Muscle Metabolism and Tendon Strength

The skeletal muscle and tendon apparatus is greatly stressed during marathons and undergoes various positive adaptive changes during training [36, 214–216]. In particular, the metabolic function of skeletal muscle is beneficially influenced by marathon training [214]. A study investigating first-time marathoners found a 48% increase in muscle V̇O_2_ after marathon training (see Table 5), suggesting impressive metabolic adaptations in muscles even without changes in V̇O_2_max values [214]. Marathon training has been proven to lead to an adaptation of muscle fiber types and metabolic enzymes [215, 217]. Early studies performed muscle biopsies in marathon runners and found an increased mitochondria count, more myoglobin, and a higher capillary density which in turn leads to an improved oxygen extraction from the blood and a more efficient energy metabolism such as an improved adenosine triphosphate (ATP) production via enzymatic adaptations in the oxidative phosphorylation pathway [215].

In addition, the muscles of endurance runners showed a higher count of Type I (slow-twitch) fibers compared to Type II (fast-twitch) fibers, which apparently demonstrate a more efficient utilization of aerobic pathways and seem to give a performance advantage as endurance runners with a higher Type I count had more success in endurance events [215]. Besides changes in muscle fiber types, endurance training seems to alter muscle architecture on a microscopic level [218, 219]. Compared to sprinters, endurance athletes show shorter fascicles and a larger pennation angle (see Table 5), which seem to have a favorable impact on muscular fatigue resistance and metabolic efficiency improving endurance performance [216, 218].

Marathon training seems to have several favorable effects on leg muscles and on the Achilles tendon to some extent [216]. An ultrasound study found the thickness of the medial gastrocnemius and tibialis anterior to increase significantly during marathon training (see Table 5) [216]. Similarly, the echogenicity decreased over time, suggesting muscle hypertrophy and improved tissue quality induced by endurance training [216]. In a similar fashion, the Achilles tendon thickness increased over the training period without a significant decrease in echogenicity [216]. These structural changes most likely provide a better transmission of muscular force to the ground, providing benefits during marathon running [216].

#### Importance of Exercise on Joint Health and Osteoarthritis

The influence of marathon training and running on degenerative joint diseases such as osteoarthritis is an ongoing debate [15, 220–223]. MRI studies already established that knee cartilage reacts to acute bouts of exercise and transiently decreases in thickness [224]. However, it is still not entirely clear how endurance exercise influences joint health on a long-term basis. Several studies have been conducted investigating the effect of marathon training and running on osteoarthritis in elite and recreational runners [15, 220–223]. An MRI study of recreational marathon runners found transient changes in meniscal signal alterations, whereas no harmful long-term effects of marathon training and 8 weeks after the race could be detected [221]. Even in former elite marathon athletes with a mean lifetime mileage of 142,000 km, no increased incidence of knee osteoarthritis was found compared to age-matched sedentary controls using MRI [223]. Another study comparing experienced marathon runners to age and BMI-matched sedentary controls found no increased prevalence of osteoarthritis in the lower extremity joints of endurance athletes [222]. Especially the overload of the natural adaptation mechanisms, together with malalignment of the lower extremities or pre-existing high-grade meniscal lesions, leads to the development of micro-damage, which should be seen as a risk factor for the development of degenerative joint alterations as precursors of osteoarthritis [220, 221]. Sufficient rest and adequate training should, therefore, not lead to an increased incidence of osteoarthritis [220]. Interestingly, a recent study comparing active experienced marathoners to age-matched controls found a prevalence of hip or knee osteoarthritis of 8.8% in endurance runners (see Table 5), which was significantly lower than the prevalence in the matched population of 17.9% [15]. These results suggest that marathon training is in fact favorable to joint health [15]. Although this study had its limitations, a recent in vitro study investigating the effects of endurance exercise on circulating progenitor cells of chondrocytes showed promising results that marathon training has a protective role on these cells by modulating the vitamin B6 salvage pathway, thus having a beneficial effect on the prevention of osteoarthritis [225]. After a half-marathon distance several genes important for chondrogenic lineage were upregulated [225]. Additionally, active forms of vitamin B6 complexes were upregulated post-race, probably by a physical activity-mediated stimulus for gut microbes to increase vitamin B6 production [225]. After marathon running, several pro-inflammatory cytokines are increased, such as IL-1β, which has negative effects on mesenchymal stem cells developing into chondrocytes [225]. In turn, vitamin B6 complexes were able to counteract these harmful effects of IL-1β on progenitor cells of chondrocytes, thus having beneficial effects on cartilage health [225]. In that way, physical activity may be able to prevent osteoarthritis [225].

#### Running Related-Injuries

There are many positive aspects of marathon training on the musculoskeletal system, such as a decreased prevalence of osteoarthritis. However, the musculoskeletal system also experiences several harmful consequences during running. Running-related injuries concerning the musculoskeletal system are one of the most common and performance-limiting problems of marathon training or races [54]. The incidence rate of running injuries varies widely, ranging from 18.2 to 92.4% [226]. Considering different athletes, approximately 56% of recreational runners and, in some cases, up to 90% of runners training for a marathon suffer at least one running-related injury per year [54]. Most of these injuries are due to overuse and are mostly located in the lower extremities [54, 226, 227]. About 70% of all injuries due to endurance running are located at or below the knees [54].

Accurate and correct data regarding frequencies of running related-injuries are difficult to obtain. Nonetheless, the most frequently mentioned injuries and corresponding incidences in systematic reviews are patellofemoral pain syndrome (5.5–22-7%), medial tibial stress syndrome (13.6–20.0%), Achilles tendinopathy (9.1–10.9%), plantar fasciitis (4.5–10.0%), ankle sprain (10.9–15.0%) and iliotibial band syndrome (1.8–9.1%) (see Table 5) [54, 226]. In this regard, Table 10 gives an overview of the most common running-related injuries in different anatomical locations. Comparing pre- and post-race MRI scans of the knee joint, an increase in radiological damage scores of lateral patella cartilage, semimembranosus tendon, iliotibial band as well as the prepatellar bursa can be regularly observed [211].

**Table 10:** Common running-related injuries in different anatomical locations [54, 226–230]

Hip injuries	Knee injuries	Shin and calf injuries	Foot and ankle injuries	Other injuries
Greater trochanteric pain syndrome	Iliotibial band syndrome	Gastrocnemius strain	Achilles tendinopathy	Low back pain
Pelvic and hip stress injuries	Patellar tendinopathy	Medial tibial stress syndrome	Plantar fasciitis	Costal fracture
Hamstring muscle injuries	Patellofemoral pain syndrome	Tibial stress fracture	Metatarsal stress fracture	
	Cartilage and meniscal injuries	Chronic exertional compartment syndrome	Ankle sprain	
	Infrapatellar bursitis			

Bone stress injuries are also commonly observed in marathon runners. Typically, a combination of chronic overload and certain risk factors leads to bone stress injuries [54]. The medial tibia, femoral neck as well as metatarsals are common sites in endurance runners that are affected by bone stress injuries [54, 226, 228, 229]. Although bone stress injuries are less common than ligamentous and muscular injuries, an incidence of tibial stress fracture of 9.1% has been reported (see Table 5) and, therefore, should not be neglected [226]. It is especially challenging, as the symptoms of bone stress injuries such as femoral neck stress fractures are often misinterpreted, and the diagnosis is made with up to 14 weeks of delay, which increases the risk of potential complications and the risk of never being able to repeat previous performance [228].

There are different intrinsic as well as extrinsic risk factors that have been identified for running-related injuries. Runners with a history of prior injury have an increased risk for new injuries concerning marathon running [54]. Furthermore, nutritional status plays an important role in running-related injuries, especially low body weight, low caloric intake or vitamin D deficiency, which has been shown to increase the risk for stress fractures [54, 228, 230]. Also, biomechanics and the running pattern seem to have an influence on injury incidence, but data collection is quite difficult, and results are not yet conclusive [54]. Regarding extrinsic risk factors, there are mainly flaws in training, excessive running distances, extreme training intensities as well as hasty increases in either running distance or intensity which make runners vulnerable to injury [54, 227]. A recent study investigating risk factors for injuries in first-time marathon runners (see Table 5) found that 9.5% of runners had major (e.g., bone stress injury) and 49.2% minor injuries (e.g., medial tibial stress syndrome), respectively, during the preparation for the marathon race [227]. Having half-marathon experience as well as at least one longer training run completed the month before the race resulted in a considerable risk reduction for injury during the marathon race [227]. Additionally, athletes completing training runs on ≥ 4 days of the week compared to those running < 4 days per week had a higher incidence of running-related injuries on the competition day [227]. Regarding sex, a Canadian study reported a BMI of > 26 kg/m^2^ in males and age younger than 31 years in females as protective for running injuries; conversely, running once a week and age older than 50 years were risk injury factors in females [231]. Participating in marathons was associated with a greater risk of injury in women than men in a systematic review on risk factors and sex differences; however, not enough studies investigated differences between male and female runners to obtain definite results [232].

#### Cartilage Health in Marathon Runners

Over the past few years, the question has arisen whether endurance running—especially long-distance endurance activity such as marathon running—leads to acute and/or long-term changes or even damage in cartilage of the lower extremities [233]. Particularly feared was the suspicion of whether long-distance running could contribute to the premature onset of osteoarthritis [233]. In this context, several MRI studies have been conducted, investigating to what extent marathon training and running influence different joints in the human body during marathon training as well as during the race. A recent study investigating hip joints of asymptomatic first-time marathon runners during a 4-month training program found MRI abnormalities in 90% of the participants before the start of the training program (see Table 5) [233]. The MRI examination after the marathon race showed in only 4.8% of marathon finishers new changes (both with small areas of bone marrow edema in the femoral heads) without any clinical complaints or corresponding symptoms [233]. Furthermore, the abnormalities found on pre-training MRI had no negative impact on the runner’s performance during the marathon race [233]. These results are further supported by other studies comparing pre- and post-race hip MRIs without any new detectable damage to the hip joints [234]. All in all, it seems that high-impact forces during marathon training and running are well tolerated, long-endurance running is safe for the hip joints and does not induce any acute damage or osteoarthritis in the hips [233, 234].

Similar studies investigating the knee have been conducted. The knee, especially its ligaments, cartilage, and menisci, is presumably more at risk for injury and mechanical stress during running than the hips [235]. An MRI study examining knee joint cartilage volume and thickness in healthy and asymptomatic marathon beginners during a 6-month intensive training program found significantly reduced cartilage volume and thickness at the lateral femur (see Table 5) [235]. Non-significant changes were found at the medial and lateral tibia, medial femur as well as patella [235]. Additionally, the measured values of cartilage loss were comparable with previously noted precision errors for MRI cartilage measurement and, therefore, are presumably not of clinical relevance [235]. Another MRI study comparing knee cartilage in marathon runners and sedentary controls after 30 min of running found no difference in cartilage deformation between both groups [224]. A further study investigating marathon runners found a knee joint effusion in 59% of runners prior to the race, of which 30.1% experienced an increase in pre-existing effusion after the race [236]. Although cartilage lesions were present in 18.2% of athletes, no worsening of these lesions was observable after a race [236]. These lesions are most likely explained by previous extensive stress, such as an overuse injury [236]. A recent systematic review performed on acute changes in knee cartilage and menisci reported a similar conclusion [237]. There were detectable transient changes in volume and signals of knee cartilage and menisci after intensive long-distance endurance exercise [237]. In this context, superficial and medial areas of the knee are likely to be more vulnerable to repetitive stress [237]. However, these changes are transient and testify to the high adaptive capacity of cartilage and menisci [224, 234, 235, 237].

In a similar fashion, there have been concerns about the possible negative effects of marathon training and running on the lower lumbar spine and intervertebral discs [238]. Few studies investigating this topic have been conducted to date. A recent MRI study investigated first-time marathon runners before and during their training period as well as after a marathon race (see Table 5) [238]. Before training, 61% of participants showed signs of disc degeneration mostly in the region of L4-S1, albeit without any clinical symptoms and no back pain [238]. After completion of the marathon race, no significant difference in lumbar spine appearance or differences in intervertebral disc height, width or intervertebral distance could be observed using a lumbar MRI examination [238]. These results suggest that marathon training and running are safe for first-time marathoners and does not have any negative effects on lumbar spine health [238].

#### Common Tendon Issues in Running

Tendon problems are a common problem in marathon runners [54, 226]. In particular, Achilles and patellar tendons are at risk of overuse injuries and often cause problems (see Table 10) [54, 226]. In recent years, the question has arisen whether marathon training and running in healthy asymptomatic runners lead to changes in the tendons and whether these can possibly be detected at an early stage to prevent possible consequential damage [239]. An MRI study investigating healthy first-time marathon runners during their training and the marathon race found a positive correlation between Achilles tendon thickness and body weight as well as height [239]. After the training and the race, a slight reduction in signal intensity at the insertion point of the Achilles tendon could be detected, which was interpreted as a beneficial adaptive mechanism to increase mechanical demands [239]. Furthermore, the volume of the retrocalcaneal bursa increased significantly, which is in line with the current literature and can be interpreted as an acute adaptive mechanism [239]. A similar MRI study investigating the effects of marathon running on the Achilles tendon found signal reductions in certain parts of the tendon, which might be suggestive of swelling or edema [240]. However, most of these changes returned to pre-race levels in a control MRI examination four weeks post-race [240]. Interestingly, athletes with foot pain or problems had significantly more lesions in the Achilles tendon compared to asymptomatic runners [239]. Over the course of the marathon training and the following race, adaptive changes in the Achilles tendon were observable, but these did not lead to any new Achilles tendon lesions [239, 240].

Similar studies investigating tendon changes induced by long-distance running have been conducted using ultrasound as an examination tool (see Table 5). A recent study investigating adolescent runners during their 6-month training period and the consecutive marathon race found increased volume and decreased echogenicity in extrinsic foot and ankle muscles such as the tibialis anterior muscle [216]. The thickness of the Achilles tendon increased significantly over this time period. However, no change in echogenicity of the Achilles tendon was detectable, suggesting normal adaptive remodeling of the tendon [216]. On the other hand, the echogenicity of the patellar tendon decreased over time, which might suggest tendinopathic changes [216]. Regardless, all participants remained asymptomatic over the course of the study; however, a long-term follow-up is not available and further research is needed [216]. Ultrasound studies performed on adult runners reported abnormalities in 24.1–24.6% of Achilles tendons and in 23.1–39.1% of patellar tendons prior to the race (see Table 5) [241, 242]. During a follow-up period, runners with tendon abnormalities were about three times more likely to develop pain within 12 months after the race regardless of age, sex, running experience, weekly training distance or pre-race pain [241, 242]. Positive and negative predictive values of developing pain within 12 months post-race with ultrasound abnormalities in the Achilles tendon were 34.1% and 87.2% and 22.9% and 85.0%, respectively, for abnormal findings in the patellar tendon [242]. In general, marathon training and running are safe for both Achilles and patellar tendon function and integrity [54, 239–242]. Nevertheless, these tendons are subjected to high stresses and are, therefore, also susceptible to overuse injuries and tendinopathic changes [54, 239–242].

#### Markers of Skeletal Muscle Damage

**Fig. 6:**
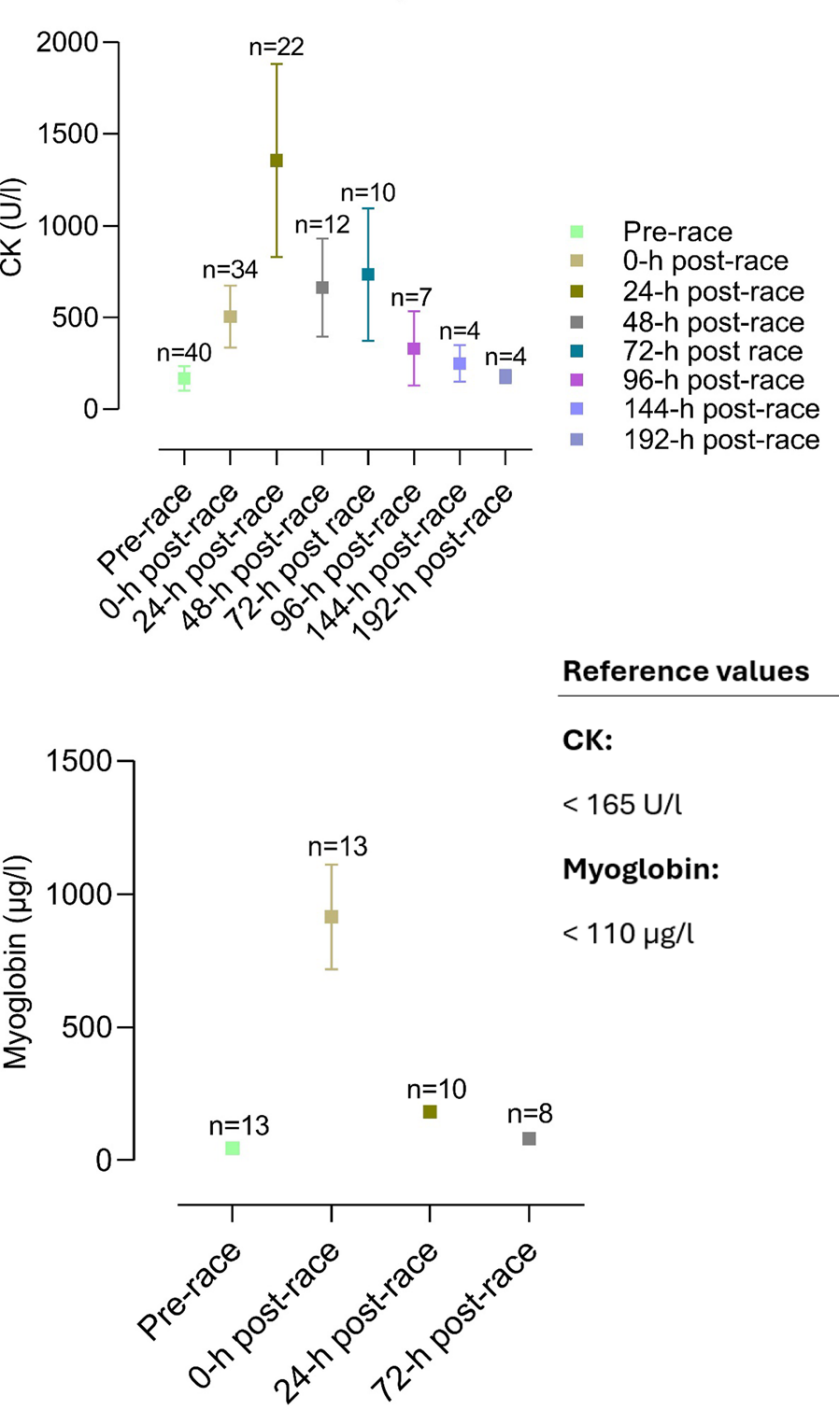
Mean CK and myoglobin changes in studies investigating biomarkers of muscle damage before and after a marathon [16, 42, 45, 46, 59, 84, 86, 88, 89, 91, 94, 102, 106, 148, 195, 196, 243–247, 251–255, 260, 311, 363]. CK, creatine kinase

In addition to injuries to bone, cartilage, ligaments and tendons, a marathon race has drastic effects on the muscles themselves. Several studies have found significant increases in CK and CK-MB in the serum of marathon runners after a race, indicating diffuse muscle damage and some rhabdomyolysis, respectively (see Table 5) [16, 45, 46, 86, 243–251]. CK values increased rapidly after the marathon and were already increased three-fold immediately after the race compared to pre-race values and reached peak values of five to tenfold compared to baseline values in the first 24 h post-race (see Fig. 6) [250, 252]. Elevated CK values are often still detectable up to two weeks post-race and then start to normalize again (see Table 5).

Similarly, elevated levels of CK-MB are also observed after marathon running (see Fig. 1), similar to the biochemical alterations during a myocardial infarction [45, 243, 244, 246, 253]. CK-MB is predominantly expressed in cardiac muscle but is also present in smaller amounts in skeletal muscle [253]. However, it can be inferred that the elevation of CK-MB may originate from peripheral sources, as muscle biopsies conducted on marathon runners have demonstrated increased CK-MB levels in skeletal muscle, while myocardial scintigraphy has revealed no cardiac abnormalities [241, 242, 247].

Additionally, increased levels of myoglobin and lactate dehydrogenase (LDH) have been observed after the completion of a marathon (see Table 5, Fig. 5, and Fig. 6), which in combination with the other described biochemical changes, indicates death of muscle cells [16, 59, 86, 244, 246, 254]. High levels of myoglobin in the circulation may become problematic as it can obstruct kidney tubules and lead to pigment nephropathy with concomitant AKI. CK and CK-MB elevations are dependent on different factors. Higher increases were observed in slower runners, suggestive of lower fitness levels [248], runners suffering from muscle cramps during the race [255], as well as different angiotensin-converting enzyme (ACE) genotypes [246]. Extensive inflammatory responses, especially elevations of IL-10 and TNFα, seem to be directly correlated with increased CK activity post-race [252]. Furthermore, prior statin intake leads to increased CK activity post-race compared to controls, suggesting an increased exercise-related muscle injury under lipid-lowering therapy [247].

Muscle biopsies confirmed these biochemical changes that indicated muscle damage. The biopsies showed specific injuries to muscle fibers, including extensive breakdown of myofibrils, loss of the sarcoplasmic reticulum, and mitochondrial abnormalities [249, 256]. Follow-up biopsies eight to 10 weeks post-race showed ongoing muscle fiber regeneration with increased satellite cell count and prominent Golgi complex as well as endoplasmic reticulum in muscle fibers, highlighting the remarkable regenerative capabilities of skeletal muscle [249, 256]. A combination of the hypoxic state in peripheral muscle tissue as well as the increased formation of free radicals with reduced antioxidative capacities is the most probable cause for muscle fiber necrosis [256]. A recent MRI study was able to detect microstructural lesions in skeletal muscle 24-h after a marathon race accompanied by concomitant CK elevation [251]. Interestingly, these changes were found in specific regions and muscle parts of upper leg muscles and were not homogeneously distributed [251]. These changes are only transient as a follow-up examination 2 weeks after the race showed a complete recovery of the upper leg muscles with similar images as in the pre-race examination [251]. This examination might especially be interesting in the future as it enables the monitoring of muscle regeneration and repair after long-distance endurance events [251].

#### Skeletal Muscle Cramps and Soreness

Exercise-associated muscle cramps are a common problem of runners during long-distance endurance events, with estimated incidences of 18% during a marathon and lifetime reaching 30–50% [255, 257]. There are several different hypotheses as to why muscle cramping occurs during long-distance endurance events such as electrolyte imbalance and dehydration [255, 257]. Usually, muscle cramps occur in a localized muscle group experiencing high stress during the activity and are therefore not well explained by systemic alterations [255, 257]. Furthermore, studies did not find a significant correlation between electrolyte imbalances and the occurrence of cramping (see Table 5) [255]. Currently, altered neuromuscular control is thought to be the most plausible cause for muscle cramping, leading to spasmodic involuntary contractions of certain muscle groups [255, 257]. Risk factors for muscle cramping experiences during a marathon include higher age and BMI, cramping in the family history as well as short or irregular stretching times [257].

Muscle soreness is a common occurrence after the race [256]. The pain usually peaks 24–48 h post-race and is best explained by muscular overuse, particularly eccentric contraction which causes higher tension in muscle fibers and leads to structural injuries [256, 258]. A recent study found an inverse correlation between muscle strength and pain sensation. Individuals with higher knee extensor strength experienced significantly less pain during the post-race period (see Table 5) and recovered faster compared to runners with lower knee extensor strength [258]. However, it is not clear whether this population may return to training faster [258]. A correlation between pre-race V̇O_2_max values and muscle soreness was not found [258].

Furthermore, a marathon race leads to muscle fatigue and influences muscular strength after the race [259]. A study investigating muscle fatigue measuring counter movement jump as well as maximal voluntary contractions of leg muscles found muscle power post marathon decreased by up to 20% and recovered only after 5 days [259].

#### Muscle Recovery

In addition to pathological changes, rapid muscle recovery is essential for athletes [252, 260]. A recent study found that the inflammatory response that occurs after a marathon is important for muscle recovery [252]. Particularly, some cytokines, especially IL-6, are important for the activation of satellite cells i.e., precursor cells of myocytes, inducing angiogenesis and myogenesis [252]. Exercise-induced muscle injury leads to a release of IL-6 and IL-6 associated with CK in marathon runners [261]. Additionally, studies found that a return to moderate running 48 h post-race could speed up neuromuscular recovery and does not lead to further muscle damage [260]. In fact, runners returning to running after 48 h post-race have significantly improved squat jump tests compared to resting runners [260]. A return to moderate physical activity 48 h after a marathon race may, therefore, be recommended to speed and enhance muscular recovery [260].

#### Risk and Benefits

The musculoskeletal system is immensely stressed during a marathon race and training. Therefore, it has enjoyed great research interest over the past years. Some relationships are not yet clear or conclusive, especially the effects of marathons on bone mineral density and osteoporosis risk, osteoarthritis risk, and long-term joint damage. In this regard, further studies should be conducted to investigate this issue in a larger patient population.

Marathon running places high demands on the musculoskeletal system. Accordingly, injuries such as Achilles tendinopathy or bone stress injuries are frequently seen in endurance athletes. This is a major problem as these injuries negatively affect performance, and the recovery path is long and often characterized by relapses. Nevertheless, marathon training also has many positive influences on the highly adaptive musculoskeletal system. In particular, energy metabolism in muscles can be positively influenced, and beneficial influences on the prevention of osteoporosis and osteoarthritis seem likely. In conclusion, despite the negative consequences, marathon training and running are mostly safe and also provides several benefits for the musculoskeletal system.

### Hematological System

Marathon running imposes a great challenge for the human body to maintain homeostatic balance [36]. In this sense, the hematological system (i.e., blood cells, blood coagulation, substrates for blood cells) is also stressed and of great importance in maintaining adequate circulation [48]. We identified 38 articles investigating the positive and negative effects of marathon training and running on the hematological system. Table 3 gives an overview of the most important findings from said studies.

**Fig. 7:**
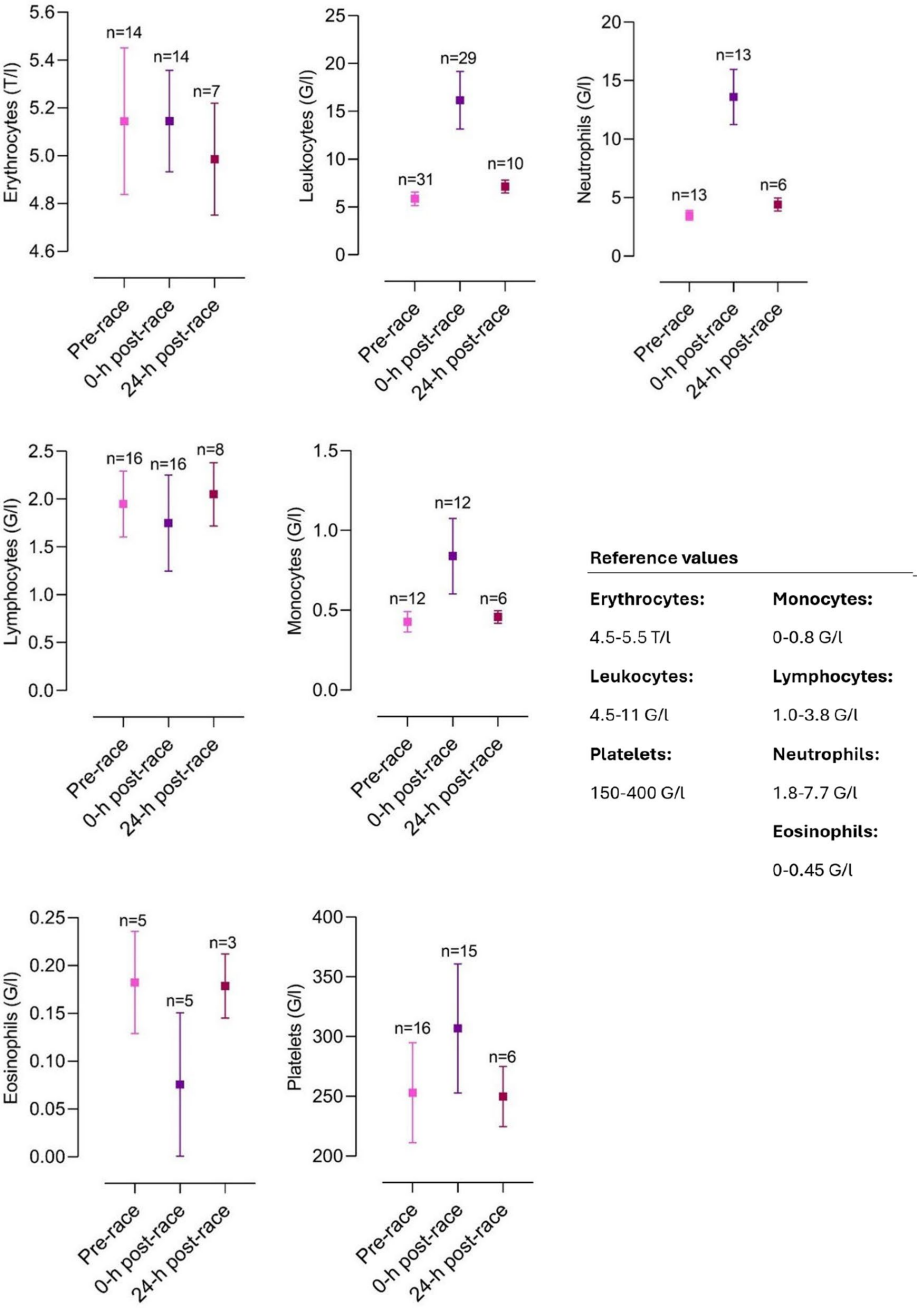
Mean changes in erythrocytes, leukocytes, platelets, and leukocyte subtypes in studies investigating total blood count before and after a marathon [16, 42, 59, 84, 94, 106, 137, 148, 196, 243–246, 254, 271–274, 286, 295, 297, 306, 308, 311, 326, 339]

**Fig. 8:**
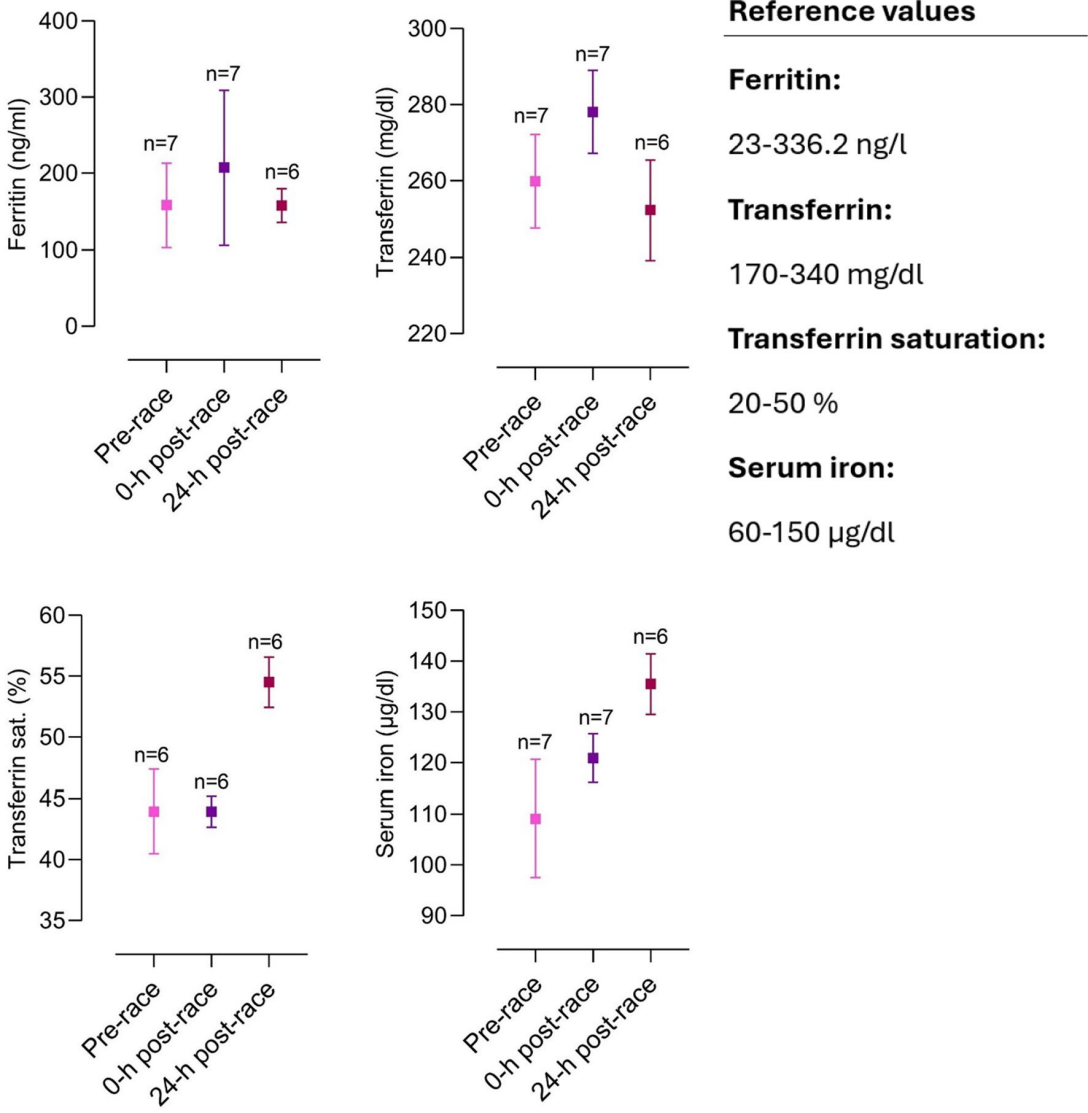
Mean changes in ferritin, transferrin, transferrin saturation, and serum iron in studies investigating the iron status before and after a marathon [16, 59, 246, 254]

**Fig. 9:**
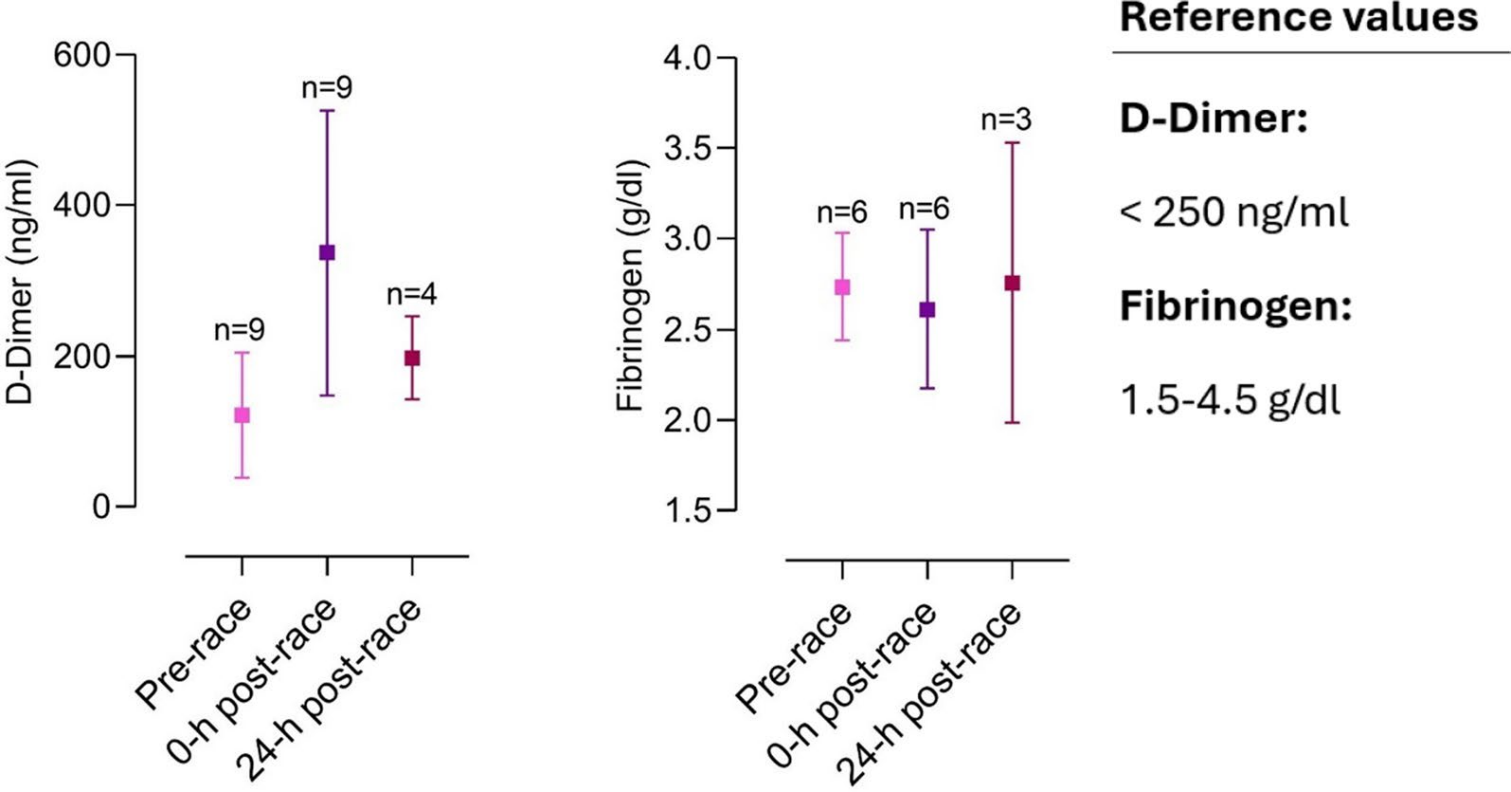
Mean changes in D-dimer and fibrinogen in studies investigating the prothrombotic state before and after a marathon [89, 244, 245, 273, 284, 287, 290, 292, 358]

A marathon race leads to several alterations in the hematological system. In this regard, studies have shown changes in total blood count (e.g., leukocytosis, see Fig. 7) [262] and in biomarkers of iron homeostasis (see Fig. 8) [16] as well as an impact of acute exercise on the coagulation cascade, fibrinolytic activity [263] and platelet function (see Fig. 9) [264]. Besides the negative effects of marathon running on the hematologic system, some beneficial changes were also observed during marathon training.

#### Exercise as a Mediator of Hematopoiesis

In this sense, recent mice studies showed that regular physical exercise influences and alters hematopoietic stem and progenitor cells due to modulation of the stem cell niche and decreased output of inflammatory leukocytes via diminished leptin signaling [265, 266]. Referring to this, the influence of leukocytes of several classes particularly macrophages, monocytes and neutrophils and chronic inflammation on the development of atherosclerosis has recently been demonstrated [265, 266]. The production rates of leukocytes directly influence disease progression [266] and blood monocyte levels have been shown to correlate with cardiovascular mortality [265]. On the other hand, moderate strenuous exercise in human studies was shown to reduce circulating inflammatory monocytes [267]. Regular physical activity thus contributes to positively limiting cardiovascular risk factors starting from the hematologic system.

Furthermore, regular endurance exercise is a potent mediator of hematopoiesis [268]. In mice, physical activity was shown to increase medullary hematopoietic stem and progenitor cell content by up to 800% and reduce marrow cavity fat by 78% [268]. These changes were promoted by an improvement in medullary niche architecture as well as an increase in skeletal muscle hematopoietic cytokine production such as IL-6, IL-8 and IL-15 [268].

#### Reduced Risk for Venous Thromboses and Pulmonary Embolisms

Venous thrombosis is a common disease with an incidence of a first thrombosis of about 1–3 per 1,000 individuals per year [269]. Usually, it manifests as a deep vein thrombosis (DVT) in the legs. However, the thrombus can detach and lead to pulmonary embolism (PE), which leads to severe consequences [269, 270]. Regular exercise reduces the risk of venous thrombosis compared to sedentary control groups (see Table 3) by about 35% [269, 270]. However, sports activities that have a high injury risk have a less pronounced effect on the reduction of venous thrombosis; therefore, sports activities with a low risk of injury should be preferred to reduce the risk of thrombosis [269].

#### Exercise-Induced Hemolysis

In contrast to the positive effects described, a marathon race can lead to several alterations of hematological biomarkers. First of all, there are signs that a marathon race may lead to a certain degree of hemolysis due to increased levels of free hemoglobin, indirect bilirubin, and LDH, as well as decreased levels of haptoglobin [16, 36, 243, 244, 254].

Nevertheless, there are quite conflicting data regarding the changes in red blood cell count, hemoglobin, and hematocrit, as well as values for mean corpuscular volume (MCV) and mean corpuscular hemoglobin (MCH) in erythrocytes. Some studies found that erythrocytes, hematocrit, and red cell indices (MCV and MCH) decreased immediately after a marathon race and returned to baseline levels within 48 h post-race [196, 254, 271, 272], whereas other studies found no changes in hematocrit or hemoglobin concentration immediately after the race or reported increased values (see Table 3) in red blood indices [16, 243, 272, 273]. These different results may be explained by different fluid consumption patterns as well as temperature and weather differences similar to those observed in EAH [49].

#### Acute Inflammatory Response

Several studies reported a significant increase in leukocytes after completion of a marathon race (see Table 3), where the increase returned to baseline levels within 2 to 7 days post-race [16, 196, 243, 244, 262, 271, 272, 274]. This leukocytosis is predominantly caused by neutrophilia, as a marathon reportedly seems to decrease total lymphocyte count (see Fig. 7) [16, 243, 271, 272, 274]. Younger marathoners showed higher levels of total white blood cells and neutrophil counts [275]. There are different hypotheses as to why exercise induces immediate leukocytosis. However, it is assumed that leukocytes are increased by an interaction of catecholamines produced during exercise that provokes leukocyte demargination and cortisol released during exercise that causes mobilization of leukocytes from the bone marrow [262]. The possible advantages of running-induced leukocytosis are not clear and may be associated with a general “fight or flight” response [262]. It does not seem to have any negative consequences on running performance or health, though [262].

#### Alterations in Iron Metabolism and Runner’s Anemia

Iron is an important component of hemoglobin, essential for oxygen transport and therefore of great importance for endurance exercise [276]. A marathon race leads to several alterations in biomarkers of iron homeostasis [16, 36]. Significantly higher levels of ferritin, serum iron, transferrin and transferrin saturation could be observed after completion of a race (see Table 3 and Fig. 8) [16, 59]. The elevation of serum ferritin can best be explained in the context of increased inflammatory and oxidative stress because it also acts as an acute-phase protein, whereas increased serum iron and transferrin saturation are best explained as a result of hemolysis [16]. These alterations remained elevated up to 5 days post-race and then returned to baseline levels, which imply that these changes are only transient [16]. More pronounced changes were observed in finishers of an ultra-marathon race, suggesting, to a certain extent, a duration or time component in changes of iron homeostasis [16].

Furthermore, iron deficiency is a common problem in marathon runners, especially in women, and may impair physical performance [16, 276, 277]. In female marathon runners, iron deficiency was present in 16–28% of athletes [276, 277] whereas < 2% of male runners were found with iron deficiency [276]. Approximately 50% of female and male runners with iron deficiency were anemic [276]. Runner’s anemia is a much-discussed topic with very contentious data [278]. Based on the data that iron deficiency is a common occurrence in endurance sports, some authors postulate that runner’s anemia is due to iron deficiency [278]. Nonetheless, only a fraction of runners with iron deficiency have anemia [276–278]. Other authors suggest changes in erythrocyte volume as the cause of anemia [36]. Marathon running, particularly through training, increases plasma volume due to a rise in total body water, elevated salt levels, as well as a higher albumin content, resulting in the dilution of hemoglobin [36, 278].

The term “pseudo-anemia” is frequently used and thought to enhance physical performance [278]. Iron deficiency should still be treated to improve running performance [276]. How this should be done, remains unclear [278]. Usually, an initial attempt at iron supplementation with a daily oral dose of 40–60 mg is recommended [279]. The efficacy of the supplementation is recommended to be monitored with blood tests 6–8 weeks after initiation of deficiency correction [279]. If oral supplementation fails to increase ferritin levels, intravenous iron supplementation may be considered [279]. Some studies found no improvement after correction of low serum ferritin or folate levels in maximal treadmill performance in female runners with iron or folate deficiency in the absence of anemia, respectively [277]. A recent study, on the other hand, found an iron overload in 15% of male and 4.7% of female runners due to the use of iron supplements [276]. This can limit performance and lead to other health problems, particularly possible side effects of iron supplementation, such as nausea, abdominal pain, and constipation [276, 278, 279]. For this reason, the use of iron supplements is recommended only for individuals with laboratory-confirmed iron deficiency [276].

#### Hypercoagulable State

In addition to changes in biomarkers of the hematological system and alterations in iron homeostasis, marathon running seems to transition the coagulation cascade into a more coagulable state [263]. There are several published cases reporting DVT [280], PE [281] and other thromboembolic events, such as retinal vein occlusion [282], in otherwise healthy marathon participants, which implicate the impact of strenuous exercise on a hypercoagulable state [283]. Several studies identified strenuous exercise as an inducer of a general prothrombotic state in the body [3, 244, 263, 273, 284–289]. This is thought to be caused by microtrauma in skeletal muscle, hemolysis, and endothelial damage, which simultaneously provokes an inflammatory response and activates both coagulation and fibrinolysis [263, 264, 290]. In this regard, some studies showed a reduction in whole blood clotting time as well as activated partial thromboplastin time (aPTT) by 7–38% after completion of a marathon [244, 263, 289].

There is evidence of increased activity of several components of the coagulation cascade such as factor VIII, von Willebrand factor, thrombin-antithrombin complex, prothrombin fragment 1 and 2, reduction in fibrinogen, as well as increased products of fibrin degradation such as D-dimers (see Table 3) [244, 263, 273, 283, 285, 287, 291]. Elevated levels of factor VIII and von Willebrand factor as well as elevated D-dimers could be observed up to 24 h after finishing a race [273, 285, 292]. Nonetheless, some participants showed imbalances where fibrinolytic activity normalized even though coagulation activity remained elevated for up to 2 days after the race [270]. This pro-thrombotic state might have severe implications, especially in runners with CAD, athletes with hereditary thrombophilia such as factor V Leiden or runners traveling by plane [3, 270, 293]. Participants traveling by plane showed a more pronounced increase in thrombin-antithrombin complex (see Table 3) as well as D-dimers after the race compared to athletes travelling less than a 2-h car trip [283, 292]. In this regard, compression socks are an effective and simple method of preventing thrombosis in individuals at risk [293]. Compression socks in participants with factor V Leiden were shown to lower overall impact on hemostasis, proven by lower thrombin-antithrombin complex (see Table 3), lower activity of tissue plasminogen activator as well as lower levels of D-dimer [293]. These changes were also observed in healthy athletes [290, 291].

In addition to changes in the blood count and hemostasis, there is evidence of significant platelet aggregation and activation as a result of vessel wall injury with consequent exposure to plated activating substances such as collagen [263]. A recent study found elevated levels of circulating endothelial- and thrombocyte-derived microparticles (see Table 3), which are cellular markers for vascular activation and damage, further supporting this evidence of thrombocyte activation followed by vessel wall injury [264]. Platelet count as well as several platelet-specific proteins such as β-thromboglobulin and platelet factor 4 were found to be elevated after marathon races [263, 264, 273, 286]. Apparently, platelet activity is greatly influenced by mechanical alteration that occurs particularly during running as significant platelet activation was mainly observed in marathons, and to a lesser extent in triathlons, but not in long-distance cycling [286].

#### Risks and Benefits

Hematological changes during marathon running and training are quite well-researched and understood. Nevertheless, there are new recent discoveries, especially the role of hematopoietic stem cells in the development of atherosclerosis and the possibility of regular physical activity to counteract this issue. However, these discoveries are mainly based upon mouse models and should be tested in human studies. Furthermore, there is still no clear consensus on optimal iron supplementation strategy in runners with iron deficiency and corresponding guidelines should be developed. Coaches and athletes should be made aware of the increased risk of thrombosis associated with air travel and marathon races. The use of compression socks in these athletes and among athletes with increased risk for thromboembolism should be encouraged.

Marathon running is associated with changes in the hematological system, especially changes in biomarkers. Nonetheless, regular physical activity such as marathon running has benefits for the hematologic system such as reduced risk of thrombosis. Conclusively, marathon training and running are normally safe and beneficial in the context of the hematological system.

### Immune System

The immune system plays a central role to protect the body from pathogens and is of great importance in restoring function. Marathon running puts immense stress on the immune system and its function [36]. Over the past decades, the role of the immune system in association with endurance exercise enjoyed much research interest. It became clear that physical activity strongly influences the immune system in various functions, both positively and negatively [17]. We identified 41 studies examining the beneficial effects as well as pathological changes of marathon training and running on the immune system. Table 6 lists the main findings and results of the said studies.

#### Biomarkers of Inflammation

In addition to the clear benefits of marathon training on the immune system, high-intensity endurance exercise also carries risks for immune function. After a marathon race, various adverse effects on the immune system may occur.

**Fig. 10:**
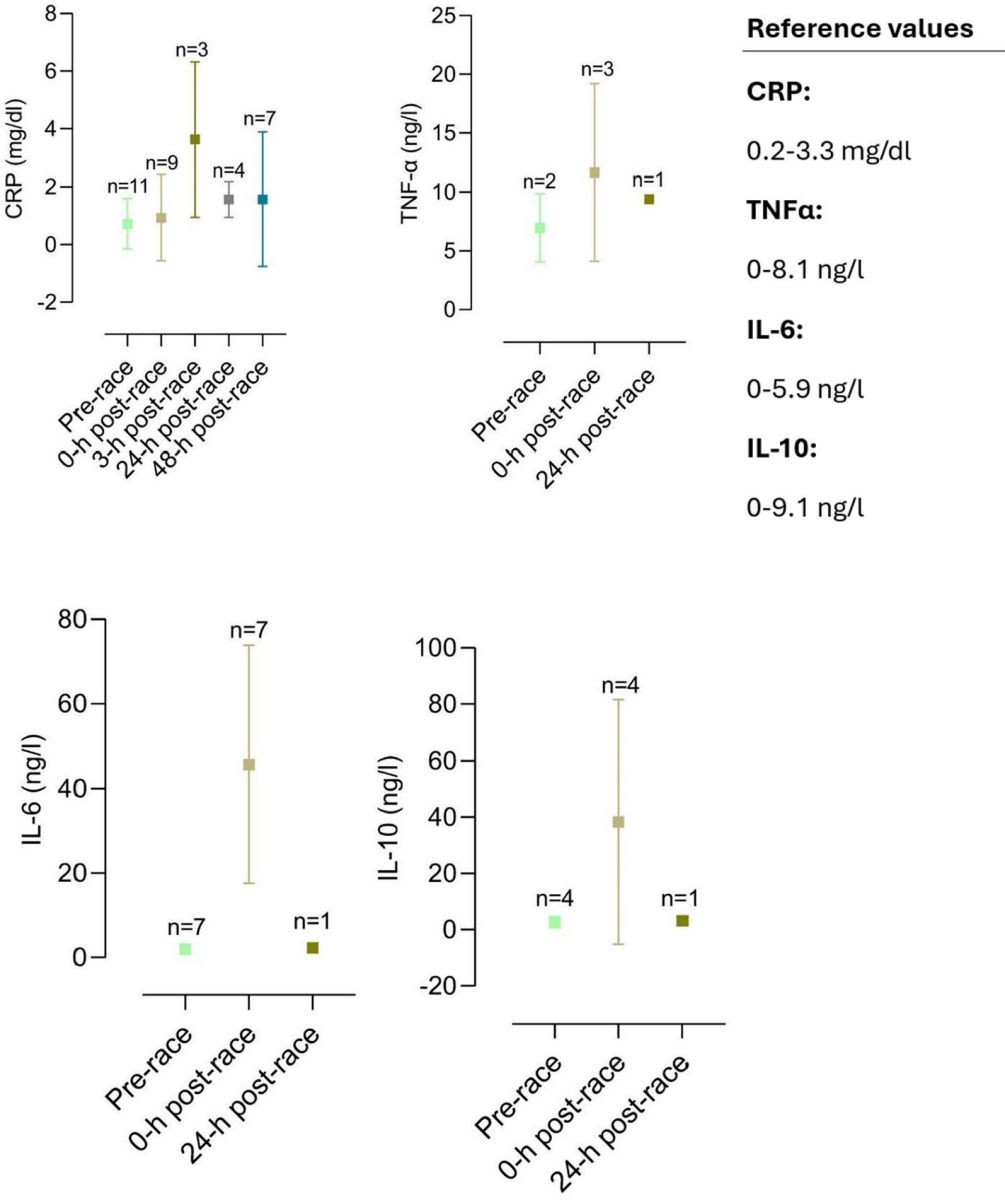
Mean changes in CRP, TNFα, IL-6, and IL-10 in studies investigating pro- and anti-inflammatory biomarkers before and after a marathon [29, 64, 86, 87, 90, 123, 133, 178, 223, 225, 226, 281, 282, 294, 304]. CRP, C-reactive protein; IL, interleukin; TNFα, tumor necrosis factor alpha

Firstly, significant changes in inflammatory biomarkers were observed. As described in the previous section, high-intensity endurance exercise leads to a significant elevation in leukocytes as well as a decrease in lymphocytes (see Table 3 and Fig. 7) [262]. Besides changes in leukocytes and lymphocytes, significant elevations in CRP [16, 59, 243, 294–296] and high-sensitive CRP (hs-CRP) [85], plasma calprotectin [297] and inflammatory cytokines such as TNFα [85, 294, 295], IL-2 [295], IL-6 [85, 137, 245, 294–296], endothelin-1 [245], and anti-inflammatory cytokines such as IL-1RA [243] and IL-10 [85, 137, 294, 296] were observed (see Table 6 and Fig. 10).

These changes in inflammatory biomarkers suggest an acute inflammatory response caused by a marathon race [16]. An increase in inflammatory cytokines could be observed immediately after a race and these usually returned to baseline levels within 4 days [85, 294, 296]. Immediately after the race, there was only a minor CRP elevation (not exceeding 6 mg/dl) and CRP began to rise after 2–7 days before returning to normal values [16, 294, 296]. The increase of CRP seems to be exercise intensity and duration dependent, as higher values can be observed in runners after an ultra-marathon compared to a marathon [16]. Interestingly, the human body seems to counteract the systemic inflammatory response immediately by secreting anti-inflammatory cytokines post-race [296]. The post-race course of anti-inflammatory cytokines is very similar to that of inflammatory cytokines, which highlights the effort of contained inflammation of the body. Anti-inflammatory cytokines returned to normal values within 4 days [85, 294, 296].

#### Acute Exercise and Risk for Upper Respiratory Tract Infections

**Fig. 11:**
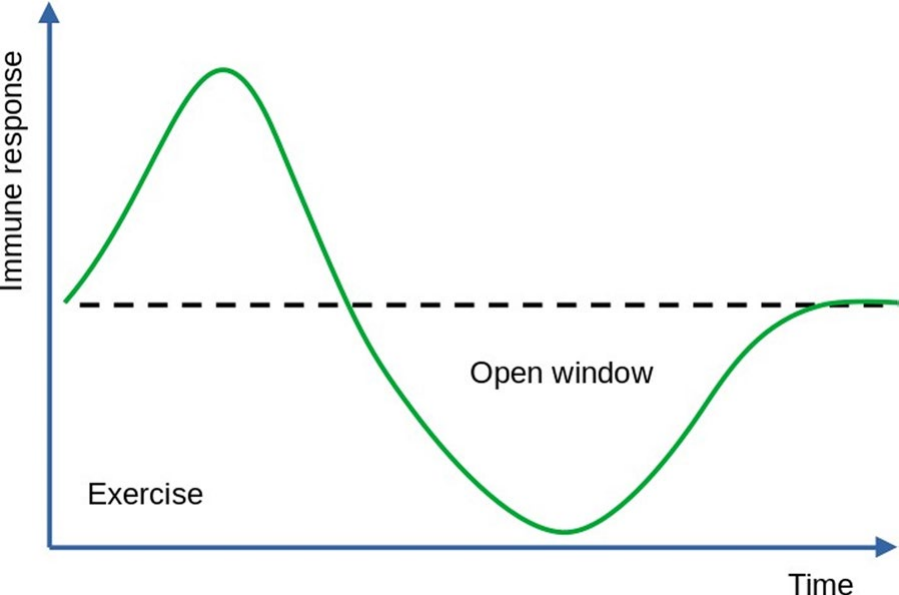
Behavior of immune function after high-intensity endurance exercise such as marathons (adapted from Åkerström and Pedersen [318], with permission)

**Fig. 12:**
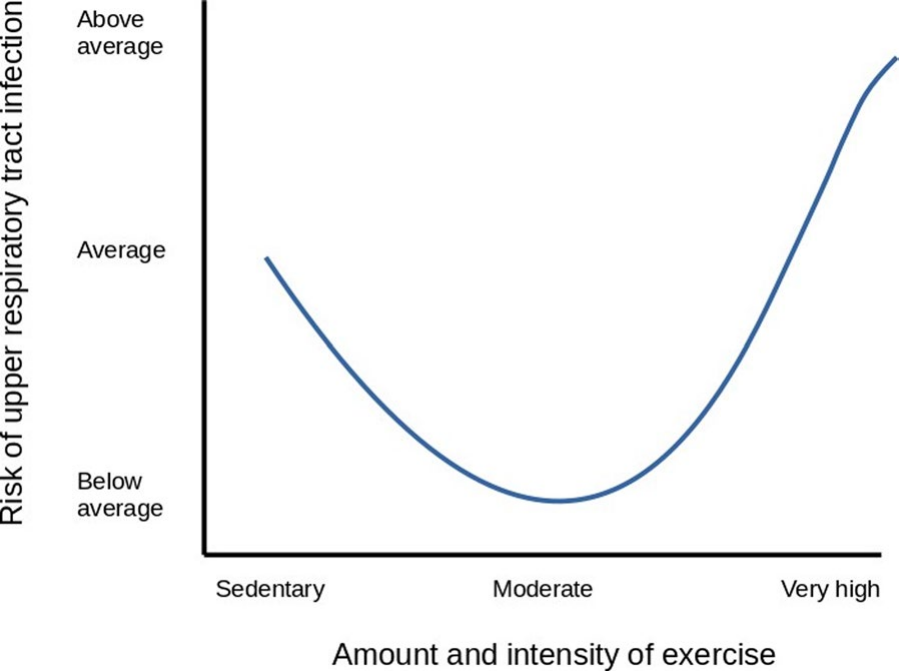
Association between exercise amount and intensity and risk of upper respiratory tract infections (adapted from Nieman [301], with permission)

Strenuous exercise seems to affect the immune system in several different ways. Various studies have found that athletes who have completed a marathon have a significantly higher risk of developing URTI compared to athletes who have not participated in a race (see Table 6) [53, 298–305]. Particularly 1–2 weeks after the race, the risk for URTIs remains elevated [53]. The peak incidence of URTI was observed in the window 3–72 h after a race, which is commonly referred to as the “open window” (see Fig. 11) [17, 306]. This observation suggests acute but transient immunosuppression due to the marathon race or intense training. Currently, the association between exercise and URTI has been established as a “J” curve (see Fig. 12) [17, 301, 303, 307]. This model suggests that moderate physical activity appears to have a beneficial effect on the immune system, with a decreased risk for URTI compared to inactivity; however with increasing duration and intensity of exercise concomitantly, the risk for URTI increases [303]. This phenomenon is supported by epidemiological data showing higher URTI incidences in athletes with either higher weekly training volumes [53] or longer race distances such as an ultra-marathon (see Table 6) [36]. In fact, several risk factors have been described that may promote URTI after a marathon race such as depression or anxiety [299], unusually intensive training periods [53, 299], overtraining syndrome [60, 300], competitive events in the winter, sleep deprivation, and low-energy diet intake [305].

There are several possible mechanisms and explanation attempts as to why a marathon race seemingly leads to a higher susceptibility to URTIs. First of all, high-intensity exercise e.g., marathon running, is associated with immunosuppression due to decreased function of immune cells i.e., neutrophils, T lymphocytes, natural killer cells (NK cells) [308]. T lymphocytes are an essential component in the recognition and control of, among other things, viral infections such as URTI [308]. As already described, a marathon race leads to a significant decrease in lymphocytes immediately after the race, which can promote an infection [17, 308]. Some studies have found a marathon-induced shift in subpopulations of T helper cells (Th cells), namely in the Th1/Th2 balance towards Th2 predominance, which lingered up to one week post-race (see Table 6) [60, 309]. Th1/Th2 balance is delicately regulated, and a decreased ratio is associated with increased susceptibility to URTI after intense physical activity such as marathon running [309]. There are other Th cell subpopulations that are affected by marathon running. Th17 cells produce IL-17, which shows strong proinflammatory effects and was even observed in connection with autoimmune diseases [137]. Regulatory T cells (Treg) are essential in inflammation control and peripheral immune tolerance [137]. In a similar fashion to the Th1/Th2 balance, the balance between Th17 and Tregs is sensitively regulated, and alterations in this ratio may lead to increased i.e., pathological, inflammation or decreased immune tolerance (see Table 6) [137]. A study investigating this balance found a decrease in CD4^+^ lymphocytes immediately after the race [137]. However, Th17 cell counts increased fourfold and Tregs decreased by 6.3-fold and remained suppressed up to 10 days post-race [137]. This observed alteration of Th17 and Treg balance indicates a shift towards inflammation and might suggest that reported URTI symptoms are caused by exercise-induced inflammation rather than an infectious etiology [137]. A different study found marathon-induced alterations in the expression of various integrins and selectins, which are important molecules that promote leucocyte-endothelial cell adhesion and enable the influx of leukocytes from the bloodstream into the corresponding tissue [306]. A marathon race led to changes in selectins and integrins so that leukocyte adherence to the endothelium was impaired and, therefore, transmigration into the tissue was hindered, leading to poorer immune function and might explain the increased susceptibility to URTI [306].

In addition to the altered function of T lymphocytes, there are data regarding a decreased NK cell cytotoxic activity after a marathon race [17]. This loss of cytotoxic activity amounts to a 62% decrease in initial function and persists for at least 6 h after high-intensity running [298, 301–303]. NK cells are a subpopulation of lymphocytes and can eliminate neoplastic and virus- or bacteria-infected cells in a non-major histocompatibility-complex mediated way [303]. Normally, NK reacts promptly to viral and bacterial infections, thus acting as a major first-line of defense against these infections [299, 301]. Therefore, the decrease in NK cytolytic activity after a marathon might contribute to the increased susceptibility to URTIs and provide a possible explanation for the “open window” hypothesis [298].

Furthermore, there is evidence that marathon running leads to a marked decrease in nasal and salivary IgA concentrations, which might promote the risk of URTI as IgA acts as the “first line of defense” against mucosal pathogens (see Table 6) [17, 303, 304, 309–311]. A recent study that administered fermented milk containing *Lactobacillus casei Shirota* for 30 days continuously pre-race showed no decreased levels in secretory IgA, suggesting a beneficial effect of this supplement [310]. Nevertheless, no statistically significant difference in the incidence of URTI was observed post-marathon, leaving the effectiveness of the intervention uncertain [310]. In a similar study examining the impact of glycan—a polysaccharide known to enhance immune function—on salivary IgA levels, a more pronounced decrease in IgA was noted in the placebo group compared to the intervention group. However, no effect on URTI incidence was reported, thereby leaving the intervention's effectiveness inconclusive [311].

**Fig. 13:**
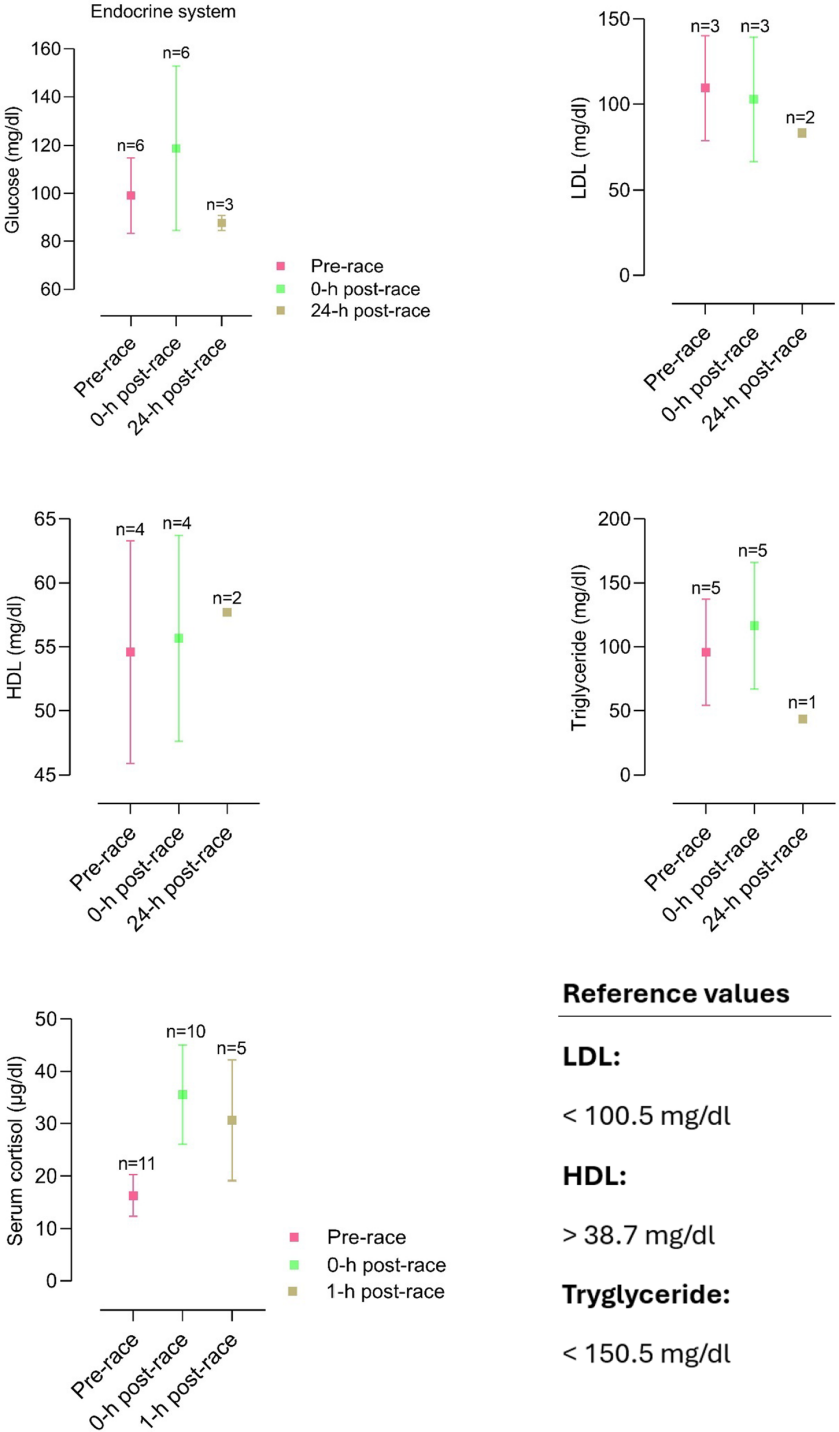
Mean changes in glucose, LDL cholesterol, HDL cholesterol, triglycerides, and serum cortisol in studies investigating endocrine biomarkers before and after a marathon [50, 62, 123, 161, 181, 280, 297, 304–306, 308, 311–314, 342, 343]. HDL, high-density lipoprotein; LDL, low-density lipoprotein

Several studies have shown that a marathon race leads to a significant elevation of cortisol levels immediately after a race (see Table 11 and Fig. 13) [57, 60, 137, 262, 312, 313]. Cortisol has well-known anti-inflammatory and immunosuppressive effects and influences Th1/Th2 balance by suppressing IL-12, an important cytokine for Th1 response, and up-regulating the Th2 response by increasing IL-4 production, thus increasing the susceptibility to infections [60, 309]. In addition to cortisol elevations, catecholamines such as epinephrine are also elevated during a marathon race [60, 262, 314]. Interestingly, they act similarly to cortisol on the immune system in the context of marathon running, suppressing Th1 cells and promoting the production of Th2 cytokines such as IL-10 [60]. In addition, there is evidence that catecholamines lower mucosal immunity, but information on this is scarce [60].

**Table 11:** Overview of key findings from studies investigating effects of marathon training and running on the endocrine system and its function

Subjects	Intervention/Observation	Outcome	References
N = 36 Female = 0 Age 43.2 ± 2.6 years	Leptin in marathon runners	A statistically significant linear correlation between leptin and marathon time could be found regardless of age and BMI	[18]
N = 40 Female = 0 Age 35.7 ± 8 years	Neuro-endocrine alterations in marathon runners	At rest, serum levels of GH were significantly higher by 1.3-fold in athletes compared to sedentary people	[325]
		After the race, serum levels of GH increased by 1.8-fold and cortisol by 3.1-fold compared to baseline values and returned to pre-race values after 72 h	
N = 6 Female = 0 Age 29.2 ± 7.4 years	Adrenal-sympathico function in highly trained marathon runners	Compared to baseline levels, glucose, free fatty acids, and glycerol were significantly increased after the race by 1.1-fold, 5.8-fold, and 5.2-fold, respectively	[314]
		Significant elevations in norepinephrine (by 1.7-fold), epinephrine (by 1.6-fold), and cortisol (by 1.6-fold) were detectable immediately after the race	
N = 9 Female = 1 Age 42.9 ± 8.5 years	Biomarker changes of pituitary-adrenal and -thyroid axis in non-elite marathon runners	After the race significant elevations of serum cortisol (by 3.7-fold), ACTH (by 5.7-fold) and vasopressin (by 2.7-fold) were observable	[108]
		No significant changes in T3, T4 or TSH	
N = 11 Female = 0 Age 56.3 (range 45–69) years	Hormonal responses of cortisol and testosterone after a marathon in non-elite athletes	One hour after the race, serum cortisol and prolactin increased significantly by 1.8-fold and 1.4-fold, respectively, whereas both total and free testosterone decreased significantly by 1.4-fold and 1.3-fold	[57]
		All parameters returned to baseline levels after a week	
		No significant changes in LH, FSH, DHEAS or androstenedione were observed	
N = 8 Female = 0 Age 35.1 ± 8.1 years	Salivary cortisol changes in non-elite athletes during a marathon race	Salivary samples were collected in 4-mile intervals during a marathon run	[312]
		Pre-race values were higher compared to other rest days	
		During the race, salivary cortisol values increased and peaked 30 min after the race with a total increase of 865%	
		Salivary cortisol levels normalized within a day	
N = 18 Female = 0 Age 25.4 ± 1.4 years	Hypothalamic gonadotropin-releasing hormone secretion in highly trained male marathon runners	Compared to healthy controls, frequency of LH pulses was reduced by 1.6-fold per eight hours as well as the amplitude of the pulses was diminished by 1.8-fold	[313]
		LH responses to increasing doses of exogenous gonadotropin-releasing hormone were decreased	
		No significant differences in FSH or testosterone levels could be detected	
N = 15 Female = 0 Age 38.3 ± 6.9 years	Neuroendocrine alterations after a marathon run	Plasma concentrations of ACTH (by 4.4-fold), cortisol (by 3.4-fold), β-endorphin (by 8.5-fold) and GH (by 2.1-fold) were significantly elevated after the marathon and reached peak concentrations 0–1 h after the race	[326]
		Levels of ACTH and cortisol returned to baseline levels after 24 h	
		GH levels normalized after 48 h	
N = 60 Female = 0 Age 35.2 ± 2.1 years	Biochemical alterations of the pituitary-thyroid axis and prolactin secretion in marathon runners	No significant changes in serum T3, T4, and TSH concentrations could be observed	[328]
		No alterations in the TSH response to TRH was detectable	
		Serum prolactin levels rose significantly after the marathon race by 1.3-fold	
N = 8 Female = 0 Age 30.4 ± 1.4 years	Alterations in gonadotropic axis in male endurance athletes	Compared to sedentary controls, mean plasma testosterone levels in endurance runners were 1.5-fold lower	[329]
		After intense and prolonged exercise, LH concentration decreased significantly by 1.6-fold in the athlete group and by 1.3-fold in the control group	
		Plasma testosterone levels increased by 1.8-fold in endurance athletes and by 1.4-fold in the control group	
N = 6 Female = 6 Age 33.2 ± 6.1 years	Alterations in gonadotropic axis and prolactin levels in female endurance athletes	Immediately after the marathon run, plasma levels of prolactin, cortisol, testosterone and DHEAS were significantly elevated by 4.0-fold, 2.1-fold, 1.9-fold and 1.5-fold, respectively, compared to baseline levels	[330]
		No significant changes in FSH or LH could be observed in this timeline	
N = 7 Female = 0 Age range 22–52 years	Effects of marathon running on the pituitary–testicular axis	Significantly elevated plasma levels of LH could be detected during the three post-run days with peak values 24 h after the race (2.4-fold)	[331]
		Prolactin and cortisol showed highest values immediately after the race, being elevated by 2.4-fold and 1.9-fold, respectively	
		Testosterone levels increased immediately after the race but fell significantly 24 h after the race by 1.4-fold and remained diminished for the following 5 post-race days	
		Noradrenaline and adrenaline levels increased immediately after the race by 2.8-fold and 3.3-fold and returned to baseline values after 24 h	
		FSH failed to show any significant changes over the course of the study	
N = 6 Female = 0 Age range 22–36 years	Hypothalamic dysfunction in overtraining syndrome	Responses of plasma cortisol, ACTH, GH, and prolactin to insulin-induced hypoglycemia in overtrained runners was lower compared their values after rest and asymptomatic runners	[61]
		Normal responses of LH, TSH and prolactin towards TRH and GnRH were observed	
		Impaired responses resolved after 4 weeks of rest suggesting a hypothalamic dysfunction in overtrained athletes	

*ACTH* adrenocorticotropic hormone, *BMI* body mass index, *DHEAS* dehydroepiandrosterone sulphate, *FSH* follicle-stimulating hormone, *GH* growth hormone, *GnRH* Gonadotropin-releasing hormone, *LH* luteinizing hormone, *T3* triiodothyronine, *T4* thyroxine, *TRH* Thyrotropin-releasing hormone, *TSH* Thyroid-stimulating hormone, *95%* CI 95% confidence interval

#### Immune-Strengthening Effect of Marathon Training

Marathon training and running affect the immune system and its function in several ways. Moderate endurance exercise especially has positive effects on the immune system. Studies have shown that regular moderate physical activity, such as marathon training, can reduce the risk of URTI, whereas intense exercise can increase this risk [299, 300, 302, 309]. In this regard, an association between the risk of URTI and exercise intensity is assumed in the form of a “J-curve” (see Fig. 12) [302]. This underlines the immune-boosting effect of regular exercise. It is assumed that both cortisol and catecholamines, with their immunosuppressive effects, are released in much lower doses during moderate exercise than e.g., after a marathon race [299]. In addition, there is evidence that the cytotoxic activity of natural killer (NK) cells is significantly enhanced, thus strengthening the first line of defense against pathogens [299, 300, 302].

Furthermore, studies have shown that regular moderate exercise, such as marathon training, can lower baseline levels of C-reactive protein (CRP) (see Table 6) [16, 243, 315]. This suggests both an immune-boosting effect and an anti-inflammatory effect of exercise [16, 315]. The reason for this is probably myokines, which are released more frequently and thus promote the release of anti-inflammatory cytokines such as IL-10 [16, 315]. This is of particular interest as high baseline CRP levels are associated with an increased risk of developing atherosclerosis [315]. In addition, the risk for cardiovascular disease or stroke is increased, whereas a low baseline CRP value is associated with a reduced risk of these diseases e.g., due to regular physical activity, among other factors [315].

In addition to lower URTI incidence and beneficial effects on baseline CRP, regular moderate endurance exercise leads to the induction of antioxidant enzymes and lower production of reactive oxygen species in mitochondria [316]. This reduces DNA damage as well as other harmful changes to cells and thus prevents cell death [316].

#### Overtraining Syndrome

Overtraining syndrome is defined as a deterioration in performance despite intense training and is associated with several symptoms, such as increased susceptibility to infections [60, 300, 305]. It is suggested that overtraining syndrome is responsible to some extent at least for transient immunosuppression [60]. This is explained by the fact that due to chronic microtrauma, an inflammatory response is constantly triggered, which is accompanied by an up-regulation of certain cytokines [60]. This leads to a disturbance of the Th1/Th2 ratio and to an increased Th2 response observed similarly to a marathon race with a decreased ability to fight off infections [60, 308, 309]. Interestingly, overtraining syndrome leads to similar biomarker changes compared to a marathon race, as elevated cortisol and catecholamine levels have been observed, which might lead to a decreased immune function [60]. Additionally, there is evidence that prostaglandin E2, an immunosuppressive molecule, is elevated after trauma such as microtrauma observed in overtraining syndrome and strongly promotes the up-regulation of Th2 cell response and decreases the activity of NK cells [17, 60].

#### Oxidative Stress

Intense, strenuous exercise such as marathon running puts immense stress on the immune system and its function [17, 317]. A study found a decreased DNA resistance to oxidative damage after a marathon race [316]. Due to the increased metabolism, especially by skeletal muscle cells or activation of phagocytes as part of an inflammatory response to microtrauma, there is an increase of reactive oxygen species, which can damage DNA strands and overload the body’s own antioxidant defense system [316]. This in turn, may lead to apoptosis of susceptible cells, especially lymphocytes, which has previously been described and might explain lymphopenia and its accompanying immunosuppression after a marathon race [137, 308, 316].

#### Role of Immunonutrition

To date, several different nutritional supplements and drugs have been investigated to positively influence the immune system during marathon training or a marathon race. Special attention was given to glutamine as it was regularly found to decrease after endurance exercise and acts as an important mediator in lymphocyte proliferation [17, 295, 318–320]. Further recognition has been given to antioxidants (e.g., vitamins C and E) [17, 318], zinc [17], bovine colostrum [318], iron [305], carbohydrates [17, 305, 318] and recently, even probiotic supplementation [308]. However, only carbohydrate intake during the race has proven itself as somewhat effective to a small extent [17, 305]. Current hypotheses assume that increased carbohydrate availability counteracts the exercise-induced immunodepression mediated via stress hormones, such as cortisol [305, 321]. However, it does not seem to have an influence on NK cell function, salivary IgA as well as incidence of URTIs [17].

#### Risks and Benefits

The immune system in the context of marathon training and running has enjoyed great research interest for decades. Numerous changes in immune function after intense endurance exercise are known, such as changes in biomarkers of the immune system, including lymphocyte count, increased susceptibility to URTI, decreased concentration of salivary IgA, and altered cytotoxic activity of NK cells. On the other hand, regular moderate endurance exercise has a protective and promoting effect on immune function, such as increased induction of antioxidant enzymes, decreased incidence of URTIs, and enhanced cytotoxic activity of NK cells.

Still, the exact mechanisms of how exercise influences immune function are only slightly understood, and possible interventions such as immunonutrition have failed so far. This calls for further research in this area to mitigate or even prevent transient immunosuppression after intense activity. Furthermore, there are are few large-scale epidemiological studies investigating the positive effects of regular endurance exercise on URTI incidence and immune function in general. Immune system and marathon training and running have been extensively researched. Nevertheless, much remains unclear. This topic is very important because many runners are affected. Therefore, it should remain the focus of current research.

In general, physical activity has a very positive effect on the immune system. However, during intensive phases of endurance exercise, there may be negative consequences for immune function. These consequences are often not very dramatic, making marathon training and running all in all safe for the immune system.

### Endocrine System

Marathon running greatly influences the balance of the tightly regulated endocrine system. Usually, the hypothalamic-pituitary axis is affected and altered by acute bouts of intensive exercise, leading to hormonal changes [36]. We identified 22 studies investigating both the positive and negative effects of marathon training and running on the endocrine system. Table 11 summarizes the key findings of said studies.

#### Beneficial Effects

Several hormonal axes are influenced by endurance exercise such as marathon training and running [36]. In particular, regular moderate exercise, such as marathon training, positively influences lipid profile in runners, i.e., lowers LDL cholesterol and triglycerides, and increases levels of HDL cholesterol, thus having a beneficial effect on the prevention of CAD (see Table 2) [3, 69]. Intense exercise, such as a marathon, leads to a reduction in body fat [36]. It can be assumed that regular endurance training reduces body fat, which in turn positively impacts running speed [322]. In addition, regular exercise is one of the basic pillars in the treatment of type 2 diabetes mellitus [18]. One possible mediator of these beneficial effects might be leptin, a hormone inducing fatty oxidation and insulin sensitivity in muscle cells [18]. Recent findings suggested a linear relationship between leptin and physical performance regardless of age and body mass (see Table 11) [18]. Furthermore, physical activity was shown to protect against recurrence and progression in breast cancer survivors [323]. Particularly in hormone-dependent tumors such as estrogen-positive breast cancer, regular physical exercise plays an essential role in prevention and risk-reduction of breast-cancer specific and all-cause mortality of said patient population as adipose tissue is the main source of estrogen in postmenopausal women [323].

#### Alterations in Stress Hormone Concentration

Nevertheless, intense bouts of exercise, such as a marathon run, can unbalance the delicately regulated endocrine system [36] and may lead to serious complications such as exercise-induced hypoglycemia in type 1 diabetes patients [324]. Several hormonal alterations have been observed after a marathon run suggestive of an acute stress reaction [57, 325]. In this regard, an acute elevation of growth hormone (GH) after a marathon run (see Table 11) has been observed in different studies [325, 326]. Presumably, GH alters metabolic pathways in such a way that is beneficial for the body to cope with the enormous strain of a marathon [325]. Concentrations of GH peaked 0 to 1h post-race and returned to baseline values within 72 h after a marathon [325]. Concomitant increases in prolactin and vasopressin have been observed, with peak values 1 h after the race and a return to baseline levels within a week [57, 108, 327].

Similarly, the hypothalamic-pituitary-adrenocortical axis seems to be immediately affected by a marathon race [57]. Several studies observed elevations in adrenocorticotropic hormone (ACTH) and cortisol after the completion of a marathon race (see Table 11) [57, 106, 108, 312, 314, 326]. Peak concentrations of cortisol were observed 1 h after a marathon and returned to baseline levels within one week after the race (see Fig. 13) [57]. Activation of the adrenocortical axis is further emphasized by increased epinephrine, norepinephrine and dopamine levels following a marathon run (see Table 11) [106, 314]. All these changes were most likely to be seen as part of a physiological stress reaction.

#### Effects of Acute Exercise on the Thyroid-Pituitary Axis

The influence of strenuous endurance exercise on the thyroid-pituitary axis is not entirely clear. Studies measuring thyroid hormones triiodothyronine (T3) and thyroxine (T4), as well as thyroid-stimulating hormone (TSH), after endurance exercise, showed contradictory results (see Table 11). Some studies reported no changes in serum levels of T3, T4 or TSH [328] whereas others showed increases in T4 following acute bouts of exercise [108]. Again other studies reported only increases in T3 after a marathon [106] whereas some reported an increase of TSH after exercise and intensity-dependent serum change of free T4 and free T3 [327].

#### Influence of Exercise on the Hypothalamic-Pituitary–Gonadal Axis

The impact of intense exercise on the hypothalamic-pituitary–gonadal axis is probably the most discussed topic regarding the endocrine system [329]. Studies showed that in marathon runners, both the mean frequency of luteinizing hormone (LH) pulses as well as its amplitude were reduced compared to controls (see Table 11) [313, 330–332]. However, a marathon run immediately leads to an increase in follicle-stimulating hormone (FSH) levels [331]. Regarding the effects of marathon training and running on testosterone levels, the literature is again divided. Most articles describe reduced levels of testosterone immediately after a marathon run (see Table 11) [18, 57, 312, 332]. Nonetheless, there is literature reporting increased levels of testosterone, especially in female athletes [313, 329–331, 333]. Despite this, said hormonal alterations, i.e., increased prolactin levels, alterations in gonadotropins, and reduced testosterone levels, have been linked to sexual dysfunction in male athletes as well as amenorrhea and other menstrual cycle abnormalities in female runners [328, 330, 334].

In addition, hypothalamic dysfunction has been described in the context of overtraining syndrome [61]. A study investigating hormonal responses in overtrained athletes found diminished responses of ACTH, GH, and prolactin to insulin-induced hypoglycemia compared to healthy controls(see Table 11) [61]. These alterations resolved within 4 weeks of rest [61].

#### Risks and Benefits

Marathon training and running interfere with the finely regulated hormonal axes of the endocrine system in both positive and negative ways. Particular beneficial effects are seen in the context of insulin sensitivity and improved lipid profile, thus favorably affecting cardiovascular health. On the other hand, negative effects especially regarding the pituitary–gonadal axis have been observed in both male and female runners.

Nevertheless, there have been ambiguous results found in the discussed studies, and consequently, the results should be interpreted with caution. In addition, most of the studies that investigated changes in the endocrine system are quite old, predominantly examined male marathon runners and differed significantly in their methodology, particularly the methods of analysis. Accordingly, it is difficult to generalize the results and further studies are needed, especially in female athletes, to investigate endocrine changes in connection with endurance sports.

In conclusion, it can be stated that most hormonal changes in marathon training and running can be interpreted as part of an exercise-induced stress reaction. However, there is evidence of disturbances in the pituitary–gonadal axis in both sexes during excessive endurance exercise, and these should be treated cautiously.

### Central Nervous System and Psychological Aspects

The effects of marathon training and running on the central nervous system have not been the main focus of sports scientists so far. Only recently, increasing interest has been paid to the topic and more research has been performed. In particular, the potential positive effects of marathon training on cognitive function would be of great clinical and practical relevance. We identified 32 studies investigating both positive and negative effects of marathon training and running on the central nervous system. The main findings of this section are illustrated in Table 12.

**Table 12:** Overview of key findings from studies investigating effects of marathon training and running on the central nervous system and its function

Subjects	Intervention/Observation	Outcome	References
N = 136 Female = 15 Age 66.0 ± 4.4 years	Cognitive function in elderly marathon runners and association with BDNF and IGF-1	The study compared elderly marathon runners to an age-matched sedentary control group	[336]
		Higher performance of the marathon runners was only observed in the Five Point Test (cognitive task) and almost significantly better in a further test. Both tests are assigned to executive cognitive abilities	
		No differences in BDNF and IGF-1 levels were observed between the study and control group	
N = 114 Female = 12 Age 66.0 ± 4.5 years	Effect of high intensity endurance training on cognitive function in elderly marathon runners	Marathon runners, long-distance cyclists and an age-matched low-physical activity control groups underwent neuropsychological testing	[337]
		No statistically significant differences were found in cognitive performances between the three different groups; nonetheless, athletes reported a significantly better quality of life compared to the low-activity group	
N = 146 Female = 31 Age 43.6 ± 10.0 years	Effects of marathon training and running on cognition and retinal vascularization	Improved performance in high-demand working memory task and improved retinal perfusion was found in the marathon group after a 6 month training period compared to sedentary controls	[19]
N = 23 Female = 0 Age 49.05 ± 5.99 years	Changes in brain volume after 38.6-km and 119.2-km mountain race	After the race, significant increase in total grey matter volume was observed in both groups	[338]
		Volume of white matter, cerebrospinal fluid and brainstem did not change	
		In the 119.2-km but not 38.6-km group volumetric increases in the thalamus, caudate, pallidus, and hippocampus was observable	
N = 51 Female = 9 Age 42.7 ± 10.2 years	Changes in serum BDNF after a marathon race	Immediately after exercise, BDNF increased by 1.1-fold compared to baseline levels	[339]
		24-h post-race BDNF levels decreased by 1.2-fold and 72-h post-race by 1.3-fold compared to baseline values	
		In a control 3 months after the race, normalized BDNF levels were shown	
N = 146 Female = 46 Age 43.6 ± 10.0 years	Influence of marathon training and running on mood and negative effect-	Compared to sedentary controls, marathon runners showed significantly fewer depressive symptoms, higher values of positive affect and happiness as well as improved general well-being	[347]
N = 261 Female = 120 Age 36.4 years	Acute effects of a marathon race on implicit and explicit memory	The study compared two groups of marathon runners. One group completed a race and the other acted as the control group	[349]
		Immediately after the race, the running group scored lower in explicit memory whereas implicit memory was improved compared to the control group	
N = 176 Female = 39 Age 47.5 (IQR 43–53) years	Changes in brain MRI after a marathon race	None of the athletes suffered a clinically symptomatic stroke during the study period	[55]
		In one subject, post-marathon MRI showed one small acute ischemic lesion in the vertebrobasilar territory (cerebellum). Nevertheless, the cerebellum was not depicted in the pre-race images, and thus there is no proof that the ischemic lesion is in fact caused by marathon running. Nevertheless, the lesion was missing in the FLAIR sequence suggesting a very recent event (within 1–24 h)	

*BDNF* brain-derived neurotrophic factor, *FLAIR* fluid-attenuated inverse recovery, *IGF-1* insulin-like growth factor 1, *MRI* magnetic resonance imaging, *95% CI* 95% confidence interval

#### Positive Effects on Cognitive Function

The influence of marathon training and running on the central nervous system is not well understood [335]. Positive effects, especially on cognitive function, have been reported, which would be of great clinical importance, especially in elderly marathoners, as dementia becomes an increasing problem in the ageing population [336, 337]. Strenuous exercise such as marathon training has been shown to be neuroprotective, to improve cognition and to beneficially influence symptom severity and onset of dementia [19, 335, 337]. In patients with mild cognitive impairment, regular endurance exercise improved global cognitive ability and memory [335]. A study comparing elderly marathon runners to a sedentary control group found significantly better performance in executive functions (see Table 12) [336]. A recent study showed a positive correlation between high-demand working memory tasks, other domains of higher-demand executive function, and marathon training [19]. Other studies described improved executive function, cognitive speed and working memory immediately after a marathon run [19]. Conversely, some studies found no effect of marathon training and running on cognitive function in elderly marathon runners (see Table 12). Still, the physically active individuals reported a significantly higher quality of life accompanied by lower depressive symptoms [337]. It is hypothesized that regular moderate exercise, such as marathon training, acts as a protective mechanism for cognitive abilities through direct beneficial effects on the cardiovascular system, improved cerebral blood flow, synaptic plasticity, increased cell proliferation, as well as neuro- and synaptogenesis in the hippocampus [19, 335–337]. In this regard, regular physical activity may slow down the progression of Alzheimer’s disease [336]. Furthermore, beneficial effects on brain white matter have been shown using MRI measurements [335]. A different study performing MRI evaluations in participants of a 38.6- and a 119.8-km mountain race demonstrated significant increases in cortical grey matter and total grey matter volume in both groups whereas the volume of the thalamus, caudate, pallidus and hippocampus significantly increased only in the ultra-marathon groups (see Table 12) [338]. These results suggest that certain brain regions are more sensitive to exercise [338]. As basal ganglia play an essential role in cognitive function, endurance exercise might, therefore, improve cognitive processes due to increased grey matter volumes in these regions [338].

Recently, the positive effects of exercise on the brain have been linked to brain-derived neurotrophic factor (BDNF), which is involved in neural plasticity, neuronal growth, synaptogenesis and neuronal survival [339]. Regular physical exercise was shown to increase the release of BDNF [339]. Nevertheless, some studies found no increase in BDNF after a marathon race [336] whereas others found an immediate increase after the race with consecutive decreases 72 h after the marathon race (see Table 12) [339].

#### Neuromuscular Modifications

Not only does cognitive function appear to be positively influenced, but regular endurance exercise also appears to have a beneficial effect on other neurodegenerative diseases such as Parkinson’s disease [340]. Engaging in regular moderate physical activity has been shown to significantly reduce the risk of developing Parkinson’s disease in a meta-analysis [341]. A case report following a 48-year-old male suffering from Parkinson’s disease in the initial stage who completed a 100-km ultra-marathon showed partial correction of abnormalities in DaTSCAN® after the race [340]. Furthermore, the patient’s medication could be reduced, and levodopa could even be discontinued without any worsening of symptoms [340]. Although neuroprotective effects of physical activity in Parkinson’s disease are still inconclusive, especially in the initial phase of the disease, regular moderate exercise should be promoted as there are hints of improved sustained striatal dopaminergic availability, production of endogenous dopamine, and improved absorption and utilization of exogenous levodopa [340]. In addition, physical activity improves balance, walking, lower limb strength, general fitness, and quality of life, along with enhanced cognitive function [340].

Quantitative research reported an increase in mental fatigue and a decrease in psychological tension and anxiety regarding the psychological effects of marathon running [342]. The questionnaire cross-sectional study based on the Illness Perception Questionnaire - Revised (IPQ-R) among ultramarathon and marathon runners indicated that recognition of the association between runners’ own decisions decision and overuse injury causation is accentuated by increased exercise loads and that runners are aware of the possibility of being harmed by a deficient inner self-criticism [343].

Another common problem associated with aging is muscle weakness and motor impairments due to reduced muscle mass, decreased strength and increased reflex latencies of lower limb muscles which predispose to an increased probability of falls [344]. A recent study investigating elderly half-marathon runners found significant central and peripheral modifications, such as an increase in the initial and average discharge rate of motor units, thus ameliorating the decline of muscle function in elderly individuals [344].

#### Pain Perception

Long-distance participants will unavoidably be confronted with a certain amount of pain during a race. Marathon runners have a proven higher pain threshold and tolerance compared to the general population [8]. Studies showed a temporary exercise-induced hypoalgesia after a marathon run [345]. This state of reduced pain sensation lasted up until one hour after the race, before the pain threshold returned to pre-race values [345]. Discussed mechanisms for this phenomenon are central pain modulatory processes and an exercise-triggered increase in endogenous opiate release [345, 346].

#### Exercise and Mental Health

Physical exercise also positively influences psychiatric conditions such as schizophrenia and depression [335, 337, 347]. Global cognition, working memory, social cognition and attention were improved in schizophrenic patients undergoing regular physical activity [335]. Additionally, moderate physical exercise such as marathon training reduces depressive symptoms (see Table 12), increases levels of positive affect and happiness and generally improves well-being [347]. Marathon runners, compared to sedentary controls, exhibit lower scores of somatic and cognitive complaints, stress, anxiety, fatigue, tension, depression, demoralization, hopelessness, and distrust [8]. Exercise should be incorporated as such into therapy for depression, as it has proven to be excellent both as a single therapy and in combination with antidepressant medication [347].

#### Neuronal Damage and Alteration in Function after Intense Exercise

Although moderate physical activity was shown to have a beneficial impact on cognitive function, extreme bouts of acute exercise, such as a marathon, seem to decrease cognitive performance during the race [19, 335]. A recent study investigating cognitive function directly after a marathon race found a slight impairment in low-demand working memory as well as reduced reaction times and accuracy [19]. In elderly marathon runners, a small impairment of long-term verbal memory was found probably in the context of exercise-induced hypothalamic-pituitary-adrenocortical dysfunction [348]. Another study found impaired explicit memory but improved implicit memory immediately after the marathon race [349]. Thus, results regarding cognitive function after long-distance endurance events such as marathon or ultra-marathon races are still inconclusive, as some studies found improvements, others found deterioration, and still others found no changes [350]. Studies assessing changes in electroencephalogram (EEG) and visually evoked potentials following a marathon race found no evidence for central nervous system dysfunction [351], whereas during marathon training, EEG studies showed a decrease in theta activity (4–6 Hz) and increase of the slow alpha component and subtheta activity (6–8 Hz) [335]. A recent MRI study investigating marathon runners found no signs of diffuse brain injury in post-marathon MRI [55]. A small asymptomatic ischemic brain lesion was found in the cerebellum in one of the marathon finishers [55]. However, as pre-race images did not depict this area, it was not certainly attributable to the marathon race [55].

#### Sleep Disorders and Changes in Autonomic Nervous System

Literature about marathon and sleep, as well as the influence of sleep pattern on marathon performance, is very limited [56]. An older study found pronounced sleep disturbance after a marathon run [352]. REM sleep was inhibited, and total sleep time was decreased immediately following the marathon, whereas marathon training did not have any impact on sleep characteristics [352]. A recent review about sleep in marathon runners found elite athletes to have poor quality and quantity of sleep due to training times, competition, stress, and anxiety, among other things [56]. An adequate amount of sleep is important for muscle recovery during training and may reduce infections or running-associated injuries [56]. Some authors attributed the sleep disruption after the marathon race to stress highlighted by elevated cortisol levels [56] and shifted balance of the ANS from a rather vagal to a sympathetic predominance [353].

This shift in ANS balance might be related to orthostatic intolerance, which is commonly observed after endurance exercise [354]. Interestingly, in a study comparing marathon finishers with orthostatic intolerance to a complaint-free group, only the non-orthostatic intolerance group showed a rise in total peripheral resistance after active standing [354]. Individuals with orthostatic intolerance showed an inappropriate activation of sinus node parasympathetic modulation before the onset of syncope [354]. The non-orthostatic intolerance group could significantly increase further sympathetic modulation of vasomotor tone during active standing starting from an already elevated baseline value post-race [354]. This suggests a sudden, centrally mediated inappropriate increase in parasympathetic tone resulting in orthostatic intolerance after the race [354].

#### Serious Neurological Emergencies

Unfortunately, there are numerous case reports of rare but serious diseases that affect the central nervous system following a marathon. As mentioned previously, EAH is a relatively common problem in marathon runners (see Table 7). In rare cases, hyponatremia-induced cerebral edema has been reported, which often leads to intensive medical treatment for those affected and might even lead to death following brainstem herniation [141]. Other authors reported strokes in two young men participating in a marathon [355], a basilar artery stroke in a 27-year-old female marathon runner [356] and a left middle cerebral artery stroke in a 14-year-old runner [357]. Fortunately, these cases are rare, but nevertheless worrying. No person suffered any permanent damage [356, 357]. Patent foramen ovale was found in the two men and thought to be the most probable cause for the stroke [355], whereas apart from oral contraception in the case of the young woman, the causes of the other cases could not be clarified [356, 357]. A combination of dehydration and the hypercoagulable state (see Table 3) was probably responsible for the strokes [356–358]. Furthermore, a case series reported an association between herniated lumbar disk and marathon running and intensive training [359]. However, recent studies evaluating lumbar spine health using MRI found no association with running and increased risk for disk herniation (see Table 5). Another dreaded complication of marathon running is exertional heat stroke [360]. This is particularly problematic in racing events that take place in subtropical or tropical areas such as Brazil or Singapore [360]. Individuals suffering from exertional heat stroke often display central nervous system dysfunction, such as confusion [360]. However, serious cases have also been reported with seizures, status epilepticus, or posterior reversible encephalopathy syndrome [360]. Whole body cooling including towels soaked in cold water, water spray, or cold water immersion are the most effective forms of treatment [361]. Prevention of EHS remains the best treatment, however. It is important that medical care is available, acclimatization of athletes is performed (i.e., gradually over multiple days, including training runs for 1–2 h), fluid and electrolyte loss are adequately restored, and athletes and coaches are appropriately educated about early symptoms of EHS [362].

#### Risk and Benefits

The influence of marathon training and running on the central nervous system has not been extensively studied so far. However, there is evidence that acute bouts of extreme endurance exercise can negatively affect central nervous system function. In this regard, cognitive impairment and even ischemic stroke have been described after a marathon race. Still, these are relatively rare events, but they should be taken seriously by athletes and coaches. On the other hand, several positive effects of regular moderate physical exercise, such as marathon training, on the central nervous system have been described. The most important of these is the neuroprotective role of regular endurance exercise, especially in patients with mild cognitive impairment, dementia or even Parkinson's disease. In addition, marathon training has a very positive influence on psychological well-being and mental health. Nevertheless, the influence of marathon training and running on the central nervous system is still not well understood. In particular, the literature on sleep and endurance exercise is scarce and there is a need for further high-quality research.

All in all, there are negative effects of marathon training and running on the central nervous system to report, but these are outweighed by the positive effects. In this sense, marathon training and running are generally safe for the central nervous system.

## Conclusion

This narrative review summarized both the positive and negative effects of marathon training and running on several different organ systems. The key findings of this review are summarized in Table 13. Endurance exercise has several proven beneficial effects on human health. For instance, every risk factor for CAD is influenced in a beneficial way, i.e., an increase in HDL cholesterol and a decrease in LDL cholesterol, a reduction in blood pressure, improved insulin sensitivity and glucose tolerance, as well as enhanced endothelial function. In addition, marathon training leads to favorable cardiac adaptations such as the so-called “athlete’s heart”, improving cardiac output. Nonetheless, extreme physical exercise also poses risks to the cardiovascular system, as suggested by increased biomarkers of cardiac injury otherwise already suggestive of myocardial infarction. Particularly, the right heart seems to be more affected by imbalanced remodeling, leading, among other things, to fibrotic remodeling with an increased risk for arrhythmias. The respiratory system is in accordance with the cardiovascular system equally stressed. While regular training improves lung function, lung volumes like FVC temporarily decrease post-race. Furthermore, marathon runners are more prone to asthma and atopic diseases. Also, kidney function is affected by marathon training and running. Patients with CKD benefit from regular moderate exercise, improving their quality of life and all-cause mortality significantly, though AKI and electrolyte imbalances such as EAH often occur after a marathon race. The GI system benefits from marathon training, reducing the risk for conditions like IBD, cholelithiasis, diverticulitis and colorectal cancer. On the other hand, the GI system is one of the most affected systems during or after a marathon race, with GI distress being frequently reported. The liver plays an important role during running by providing adequate energy metabolites. Regular moderate exercise helps lower hepatic inflammation and decreases the risk of developing HCC, although acute liver failure may occur during or after a marathon race, mostly in the context of EHS. The musculoskeletal system is immensely stressed by mechanical force and stress due to the continuous strain. However, positive effects such as decreased incidence of knee osteoarthritis and higher bone mineral density have been observed, although running-related injuries remain a common concern and pose a great risk to a runner’s performance. The hematological and immune system work intertwined and are similarly taxed, with marathon training inducing hematopoiesis, thus reducing inflammation, but also posing pro-thrombotic risks and short-term immunosuppression, increasing the risk for URTI. Furthermore, marathon training and running interfere with the delicately regulated endocrine system. Endocrine functions improve, particularly in insulin sensitivity and lipid profile, yet hormonal disruptions occur shortly after a marathon. Lastly, the central nervous system, alongside psychological traits, benefit from neuroprotective effects, improved cognition, and mental health, though sleep disturbances are common post-race, and rare events like ischemic stroke or seizures can occur.

**Table 13:** Key findings of both benefits and risks of marathon training and running on the different organ systems discussed

	Benefits	Risks
Cardiovascular system	Improved lipid profile	Elevations of biomarkers of cardiac injury and congestion
	Lower resting arterial blood pressure and heart rate	Cardiorenal syndrome
	Improved endothelial function	Major cardiac events
	Balanced remodeling with consecutive increased cardiac output	Increased incidence of AF, especially in male athletes
	Decrease in QT variability	Structural remodeling of the RA and RV
		Diastolic dysfunction of the RV
Respiratory system	Improved lung function	Reduced lung volumes and diffusion capacity after a marathon race
	Decrease in age-related decline of lung function	Respiratory muscle fatigue
		Increased prevalence of asthma, allergy, and atopy
Renal system	Slowed disease progression in CKD patients	AKI
	Better quality of life and reduced all-cause mortality in CKD patients	Signs of acute tubular necrosis and hematuria
		Fluid and electrolyte imbalances e.g., EAH
Gastrointestinal system	Improved GI motility and transit times	GI distress
	Symptom relief in IBD and IBS patients	Delayed gastric emptying
	Reduced risk for diverticulitis, cholelithiasis, colorectal cancer	Fecal blood loss with possible anemia
	Reduced cancer-specific and overall mortality in colorectal cancer patients	Rare cases of ischemic colitis
Liver	Decreased inflammation and fibrosis in NAFLD	Elevated liver enzymes and biomarkers of cholestasis
	Improved lipid, glucose metabolism and insulin sensitivity	Acute liver failure in the context of EHS
	Improved liver function in chronic hepatitis C infections	
	Decreased portal hypertension in cirrhotic patients	
	Reduced risk of HCC	
Musculoskeletal system	Increased bone mineral density	Elevated biomarkers of muscle damage
	Improved energy metabolism in skeletal muscle and muscle fiber adaptations	Exercise-associated muscle cramps
	Beneficial tendon adaptations	Muscle soreness and fatigue
	Lower prevalence of osteoarthritis in the lower extremity joints	Running-related injuries e.g., medial tibial stress syndrome, Achilles tendinopathy, bone stress injuries
		Transient joint abnormalities
		Tendon abnormalities with risk for future pain
Hematological system	Altered hematopoietic stem and progenitor cells with decreased output of inflammatory leukocytes	Significant leukocytosis, lymphopenia and changes in iron homeostasis
	Reduction in circulating inflammatory monocytes	Pro-thrombotic state with increased risk for thromboembolic events
	Mediator of hematopoiesis	Activation of platelet aggregation
	Reduced risk for venous thrombosis	
Immune system	Reduced risk of URTI after regular moderate exercise	Significant leukocytosis, lymphopenia and changes in inflammation biomarkers
	Improved cytotoxic activity of NK cells	Increased risk for URTI after intense exercise
	Lower CRP baseline levels	Imbalance in Th1/Th2 and Th17/Treg ratios
		Decreased activity of NK cells after intense exercise
		Decreased salivary IgA
		Increased oxidative stress
Endocrine system	Beneficial lipid profile i.e., lower levels of LDL cholesterol and higher levels of HDL cholesterol	Impaired function of hypothalamic-pituitary–gonadal axis
	Improved insulin sensitivity	Hypothalamic dysfunction in overtraining syndrome
Central nervous system and psychology	Neuroprotection	Cognitive impairment after extreme exercise
	Improved cognitive function	Sleep disturbance
	Increased pain threshold	Shift in ANS balance
	Reduction of depressive symptoms	Rarely, cerebral edema, ischemic stroke and seizures in context of EHS

*AF* atrial fibrillation, *AKI* acute kidney injury, *ANS* autonomous nervous system, *CKD* chronic kidney disease, *CRP* C-reactive protein, *EAH* exercise-associated hyponatremia, *EHS* exertional heat stroke, *GI* gastrointestinal, *HCC* hepatocellular carcinoma, *HDL* high-density lipoprotein, *IBD* inflammatory bowel disease, *IBS*, irritable bowel syndrome, *IgA* immunoglobin A, *LDL* low-density lipoprotein, *NAFLD* non-alcoholic fatty liver disease, *NK* natural killer cell, *QT* QT interval, *RA* right atrium, *RV* right ventricle, *Th* T helper cell, *Treg* regulatory T cell, *URTI* upper respiratory tract infection

Athletes, coaches, and event organizers should be informed about the potential risks of marathon running, including serious, life-threatening events, to ensure proper prevention measures. Key aspects such as adequate fluid consumption during the race should be implemented and fluid consumption should be included in the training.

This review, however, has some limitations due to the heterogeneity of marathon runners, ranging from adolescents to runners aged above 80 years, differing in sex, and performance levels (i.e., elite vs. recreational runners). This may impair the generalizability of certain findings as there was no individual analysis regarding age, sex, and performance level. For example, elite runners might show a less pronounced reaction of some biomarkers due to their training volume. Additionally, event locations and types of marathon races were not considered (e.g., weather, ambient temperature, climate, altitude, mountain marathon, downhill race, etc.). This further impairs generalizability as certain confounding factors might be present. The lack of quality assessment for included studies may also introduce selection bias, weakening the generalizability and reliability of the conclusions.

Despite these limitations, this review has several strengths that should be considered. A comprehensive overview of the benefits and risks of marathon training and running on various organ systems was given. To our knowledge, this is the first review to extensively weigh both positive and negative effects on multiple systems, providing valuable context for future research. The analysis of biomarker changes offers crucial insights, providing valuable context for further studies. Given the widespread popularity of marathon running this review has significant implications for athletes, coaches, event organizers, sports scientists, and sports medicine practitioners.

Many aspects remain contradictory or unclear, particularly the long-term effects of marathon running, both recreational and competitive, on cardiac and renal function, which are still poorly understood. Additionally, potential sex differences in marathon-mediated physiologic and pathophysiologic adaptations of organ systems have not yet been well studied and warrant further investigation.

In conclusion, considering the overall benefits and risks of marathon training and running, the positive effects seem to outweigh the negative impacts on the different organ systems. Most adverse effects appear after intense exercise, such as the marathon race itself, and typically resolve within a few days. Regular moderate endurance exercise, such as marathon training, consistently improves both physical and mental health. In this sense, marathon training in particular is overall safe for human health and should be further encouraged. Nevertheless, current and aspiring marathon runners should engage in open, transparent discussions with their coach and sports medicine professionals to fully consider the benefits and risks of marathon training and racing. Through shared decision-making, they can weigh potential advantages against risks in alignment with individual preferences and goals, ensuring that training choices are tailored to personal values and desired outcomes.

## Data Availability

Not applicable.

## Abbreviations

ACE: Angiotensin-converting enzyme
ACTH: Adrenocorticotropic hormone
ADH: Antidiuretic hormone
AF: Atrial fibrillation
AKI: Acute kidney injury
AKIN: Acute kidney injury network
ALP: Alkaline phosphatase
ALT: Alanine transaminase
ANP: Atrial natriuretic peptide
ANS: Autonomic nervous system
aPTT: Activated partial thromboplastin time
AST: Aspartate transaminase
ATP: Adenosine triphosphate
BDNF: Brain-derived neurotrophic factor
BMI: Body mass index
CAD: Coronary artery disease
CK: Creatine kinase
CKD: Chronic kidney disease
CK-MB: Creatine kinase myocardial band
CRP: C-reactive protein
DL_CO_: Diffusing capacity of the lung for carbon monoxide
DVT: Deep vein thrombosis
DXA: Dual-energy X-ray absorptiometry
EAH: Exercise-associated hyponatremia
ECG: Electrocardiogram
EEG: Electroencephalogram
EHS: Exertional heat stroke
FEV1: Forced expiratory volume in 1 s
FRS: Framingham risk score
FSH: Follicle-stimulating hormone
FVC: Forced vital capacity
eGFR: Estimateed glomerular filtration rate
GGT: Gamma-glutamyltransferase
GH: Growth hormone
GI: Gastrointestinal
GLP-1: Glucagon-like peptide 1
HCC: Hepatocellular carcinoma
HDL: High-density lipoprotein
HRV: Heart rate variability
hs-cTnT: High-sensitive cardiac troponin T
HVPG: Hepatic venous pressure gradient
IBD: Inflammatory bowel disease
IBS: Irritable bowel syndrome
ICD: Implantable cardioverter-defibrillator
Ig: Immunoglobulin
IL: Interleukin
KIM-1: Kidney injury molecule-1
LA: Left atrium
LDH: Lactate dehydrogenase
LDL: Low-density lipoprotein
LH: Luteinizing hormone
LV: Left ventricle
LVEF: Left ventricular ejection fraction
MCH: Mean corpuscular hemoglobin
MCV: Mean corpuscular volume
MEP: Maximum expiratory mouth pressure
MET: Metabolic equivalent of task
MIP: Maximum inspiratory mouth pressure
MRI: Magnetic resonance imaging
NAFLD: Nonalcoholic fatty liver disease
NASH: Nonalcoholic steatohepatitis
NGAL: Neutrophil gelatinase-associated lipocalin
NK cells: Natural killer cells
NSAID: Non-steroidal anti-inflammatory drugs
NT-proBNP: N-terminal prohormone of brain natriuretic peptide
OR: Odds ratio
PE: Pulmonary embolism
PEF: Peak expiratory flow
PIF: Peak inspiratory flow
PM: Particulate matter
RA: Right atrium
RAAS: Renin–angiotensin–aldosterone system
REDs: Relative energy deficiency in sport
RIPC: Remote ischemic preconditioning
RV: Residual volume
RV: Right ventricle
RVEF: Right ventricular ejection fraction
SCD: Sudden cardiac death
SGLT1: Sodium-dependent glucose-1 transporter
SIADH: Syndrome of inappropriate antidiuretic hormone secretion
sRANKL: Soluble receptor activator of NF-κB ligand
T3: Triiodothyronine
T4: Thyroxine
Th cells: T helper cells
TLC: Total lung capacity
TNFα: Tumor necrosis factor alpha
Treg.: Regulatory T cell
TSH: Thyroid-stimulating hormone
URTI: Upper respiratory tract infection
V̇_E_: Minute ventilation: VIP: vasoactive intestinal polypeptide
V̇O_2_max: Maximal oxygen consumption
